# Utilizing perhalopyridine-based alkynes as suitable precursors for the synthesis of novel poly(1,2,3-triazolyl)-substituted perhalopyridines†

**DOI:** 10.1039/d4ra05861e

**Published:** 2024-09-27

**Authors:** Fereshteh Khorasani, Reza Ranjbar-Karimi, Kazem Mohammadiannejad

**Affiliations:** a Department of Chemistry, Vali-e-Asr University of Rafsanjan Rafsanjan 77176 Islamic Republic of Iran karimi_r110@yahoo.com +98-343-131-2429 +98 391 320 2162; b NMR Laboratory, Faculty of Science, Vali-e-Asr University of Rafsanjan Rafsanjan 77176 Islamic Republic of Iran

## Abstract

A novel series of poly(1,2,3-triazolyl)-substituted perhalopyridines 5a–f were successfully synthesized from the click reaction of the terminal alkynes (drived from the nucleophilic substitution reactions of PFP 1a and PCP 1b with excess amounts of propargyl alcohol) with aryl azides 4a–c under ultrasonic irradiation. Likewise, the sonication of reaction mixtures containing pyridyl cores 3, alkyl bromides 6a,b, and NaN_3_ under one-pot conditions afforded their respective aliphatic 1,2,3-triazoles 7a–d in yields ranging from 71% to 83%. We next developed an effective method for the regioselective preparation of 2,3,4,5-tetrachloro-6-(prop-2-yn-1-yloxy)pyridine 3c through S_N_Ar reaction of PCP with propargyl alcohol without the utilization of any catalyst. It was then used to fabricate several ((1,2,3-triazol-4-yl)methoxy)-3,4,5,6-tetrachloropyridines 8a–c under the reaction conditions. Finally, the Pd(PPh_3_)_4_-catalyzed SMC reaction of tris-triazoles 5b,e with arylboronic acids 9a–c offered a practical method for the synthesis of biaryl-embedded poly(1,2,3-triazoles) 10a–f in good yields.

## Abstract 2

A novel series of poly(1,2,3-triazolyl)-substituted perhalopyridines were synthesized under ultrasonic irradiation. We also developed an effective method for the preparation of ((1,2,3-triazol-4-yl)methoxy)-3,4,5,6-tetrachloropyridines.

## Graphical abstract

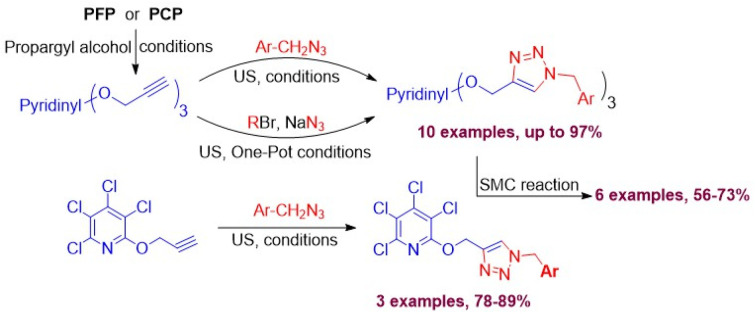

## 1. Introduction

Organofluorines (OFs) exhibit a wide range of applications in liquid crystals,^1^ agrochemicals,^2^ electro-optic devices,^1*a*,3^ and ^19^F-magnetic resonance imaging (MRI).^4,5*a*^ Proteins and peptides tagged with the fluorine-18 isotope are also applied as radiotracers in positron emission tomography (PET).^5^ On the other hand, Organochlorines (OCs) find extensive use as building blocks for chemical research and industrial uses.^6–10^ Among these, perhalogenated pyridines offer a high degree of structural diversity and have proven to be useful for searching new materials and therapeutic leads. Using perhalopyridines instead of pyridine itself is a suitable alternative method for the synthesis of substituted-perhalopyridine derivatives. In this regard, the nucleophilic aromatic substitution reactions (S_N_Ar) of pentafluoropyridne (PFP) and pentachloropyridine (PCP) with different species including oxygen,^11^ nitrogen,^12^ sulfur,^12*e*,13^ and carbon-centered^12*e*–14^ nucleophiles have been explored. The site-selectivity of reactions is overally determined by several factors including the positioning of halogen atoms on the pyridine core, solvent, the reaction conditions, as well as the strength and nature of nucleophile and base. In general, the replacement of halogen atoms on the pyridine cores takes place in a selective and step-by-step manner.^12*e*,15^ While substitution reaction of PFP with stoichiometric amount of nucleophiles under mildly basic conditions occurs solely at the C-4 position, it can be substituted sequentially at two positions (C-2, C-6) using strong nucleophiles under harshly basic conditions or elevated temperatures. On the basis, Iacono *et al.* succeeded to synthesize 2,3,5,6-tetrafluoro-[*N*-(3-aminopropyl)-ε-caprolactam]-4-pyridine *via* the site-selective reaction of PFP with 1,8-diazabicyclo[5.4.0]undec-7-ene with PFP.^16^ The controlled regio-selective S_N_Ar reaction of PFP with 4-ethynylphenol and phenol has also been led to the formation of 2,6-bis(4-ethynylphenoxy)-3,5-difluoro-4-phenoxypyridine, as a unique polymer precursor suitable for thermal polymerization.^17^ Likewise, fluoropyridyl silicone-based oils and network elastomers have been synthesized through hydrosilylation of the derived monomers through regio-controlled functionalization of PFP with alcohols possessing terminal alkenes.^18^ Alongside these, semifluorinated trifunctional-ene monomers, which play a crucial role in thiol–ene thermoset materials, are synthesized *via* the site-selective S_N_Ar reactions of PFP with 4-penten-1-ol or eugenol.^19^ PFP has also been assessed as an activator in the synthesis of acyl fluoride from carboxylic acids.^20^ Recently, there has been research conducted on the reaction of pentachloropyridine. *o*-Perhalopyridines have been utilized as amination agents under photocatalyst conditions.^21^ Among these research, 2′,3′,5′,6′-tetrachloro-4-(dimethylamino)-[1,4′-bipyridin]-1-ium treated with oxygen and nitrogen nucleophile.^11*e*,22^ Our team has previously evaluated the site reactivity of PCP with indoles^12*c*^ and *N*-substituted sulfonamides^12*d*^ as potential bioactive candidates. Another report is presented on the intramolecular cyclization of 4-phenylsulfonyl-2,3,5,6-tetrachloropyridine, which results in the rapid formation of a range of novel benzothienopyridine-*S*,*S*-dioxide frameworks under gentle reaction conditions.^23^ Ehlers *et al.*^24^ investigated the site-selective reaction of pentachloropyridine and aryl boronic acid for the synthesis of mono- and disubstituted pyridine rings. The cross-coupling reaction between cyclobutanone *O*-perchloropyridin-4-yl oxime and nitrostyrene presents an environmentally sustainable approach for the production of cyanoalkylated alkenes.^25^

**Scheme 1:**
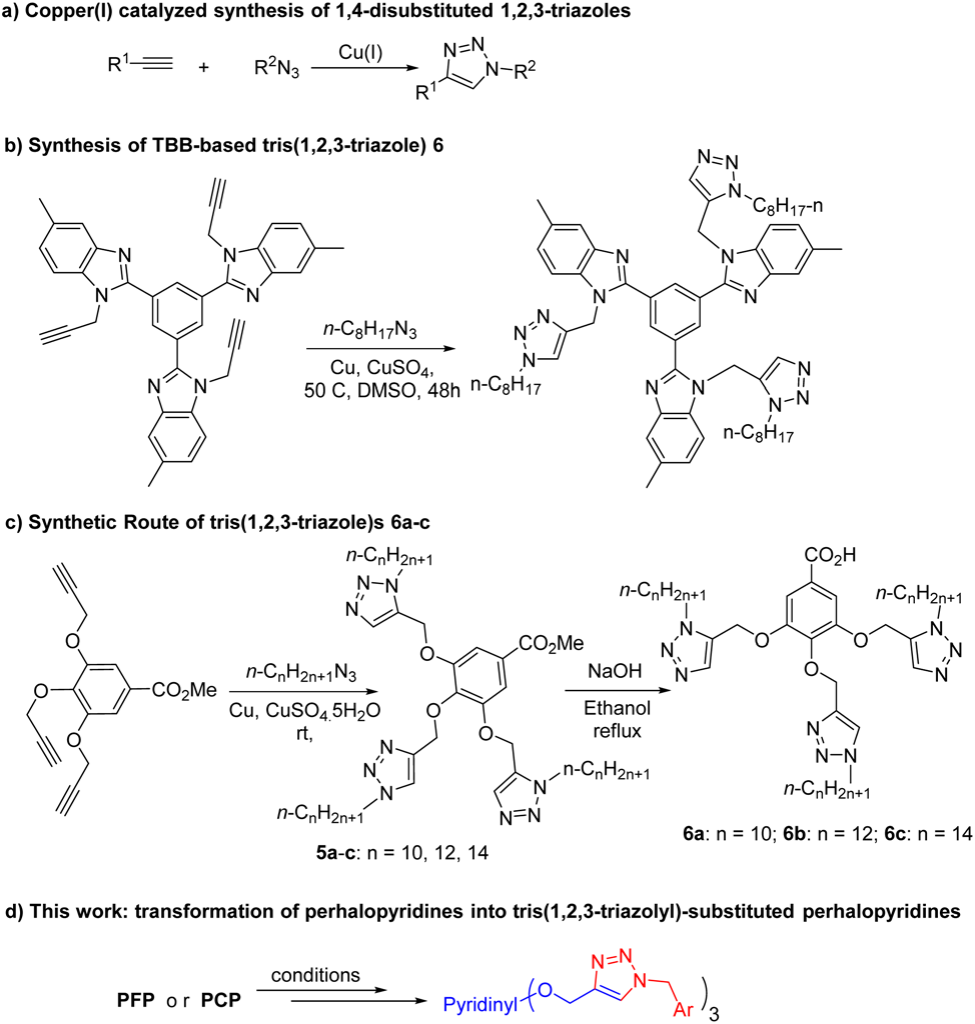
An overview.

1,2,3-Triazoles are receiving increasing interest of both academia and industry for their applications in the synthesis of dyes,^26^ photographic materials,^27^ agrochemicals,^28^ photo-stabilizers,^29^ and as linkers for binding two biologically potent scaffolds.^30^ Although 1,2,3-triazole motifs are not normally found in nature, versatile biological activities have been specified for them.^31–37^ Copper(i)-catalyzed 1,3-dipolar cycloaddition (CuAAC) reaction of organic azides with terminal alkynes has become an efficient and rapid method for the regioselective synthesis of 1,4-disubmitted 1,2,3-triazoles (Scheme 1a).^30*d*,38^ On the other hand, the introduction of polytriazole moieties into chemical structures has been proved to be a beneficial strategy in the synthesis of macromolecular 1,2,3-triazole-based functional materials and drug design.^39^ For instance, a number of synthesized bistriazoles have been considered as potent antibacterial, antifungal and plasmin inhibitors.^39*a*,40^ Xiong *et al.* have succeeded to synthesize a 1,3,5-tri(1*H*-benzo[*d*]imidazole-2-yl)benzene(TBB)-based tris(1,2,3)triazole as a selective and highly sensitive fluorescent chemosensor for the detection of picric acid (Scheme 1b).^41^ Gallic acid-based tris(1,2,3-triazole)s have also been made by a click reaction to form their respective supramolecular columnar liquid crystals (Scheme 1c).^42^ likewise, tris-(benzyltriazolylmethyl)amines have been offered as powerful stabilizing ligand for copper(i).^39*g*^ Moreover, various state-of-the-art click chemistry strategies have been developed for the formation of poly(1,2,3-triazole)-based materials with interesting application in molecular recognition, chemical sensing, drug chemistry, biochemistry, and conducting materials.^39*c*–*e*,43^ Despite all these achievement and progress, there are a few reports on the synthesis of non-polymeric poly(1,2,3-triazole) analogues. Furthermore, the incorporation of perhalopyridine units in 1,2,3-triazole scaffolds opens new avenues for seeking new ploytriazoles with sought after functions in materials science and medicinal chemistry. On the basis and in continuation of our ongoing research on perhalopyridines,^11*a*,*b*,44^ we were interested to report our findings on the synthesis of a novel series of tris(1,2,3-triazolyl)-substituted perhalopyridines (Scheme 1d).

## 2. Results and discussion

### 2.1. Synthesis poly(1,2,3-triazolyl)-substituted perhalopyridine derivatives

**Scheme 2:**
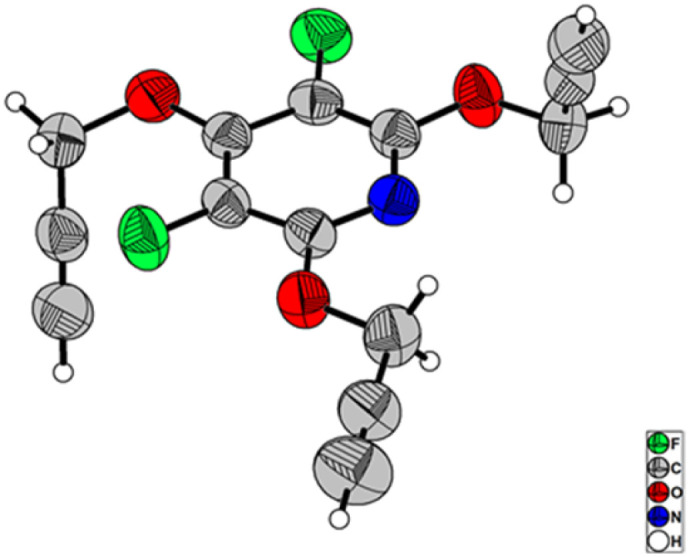
Crystal structure of 3a.

Our initially efforts were directed towards the synthesis of perhalopyridine-based alkyne precursors. As depicted in Table 1, 3,5-difluoro-2,4,6-tris(prop-2-yn-1-yloxy)pyridine 3a and 3,5-dichloro-2,4,6-tris(prop-2-yn-1-yloxy)pyridine 3b were synthesized through the nucleophilic substitution reactions of PFP 1a and PCP 1b with propargyl alcohol 2, respectively. We first aimed to find the best reaction conditions for the preparation of precursor 3a. In this respect, the reaction of 1a and propargyl alcohol was carried out under different conditions and the results are shown in Table 1. Stirring a mixture of PFP (1 mmol), 2 (3 mmol), and K_2_CO_3_ (3.9 mmol) in various solvents including CH_3_CN, DMF, DMSO, and THF at room temperature for 48 hours, led to the producing of 3a in yield ranging from 28% to 60% (entries 1–4). Hence, performing the reaction in DMF solvent using higher molar ratios of reactants was investigated to improve the efficiency of the reaction. While, the treatment of PFP (1 mmol) with 2 (6 mmol) and K_2_CO_3_ (7.8 mmol) in DMF at r.t for 48 hours afforded product 3a in the yield of 81% (entry 5), a similar efficacy was also observed when PFP and 2 were used in a 1 : 9 molar ratio (entry 6). However, we succeeded to isolate product 3a in a yield of 93% by lengthening the reaction time to 72 hours from the r.t reaction of PFP (1 mmol) with 2 (6 mmol) and K_2_CO_3_ (7.8 mmol) in DMF (entry 7). In continuation, the influence of several factors such as solvent, reactants' molar ratio, and reaction temperature was studied on the reaction of PCP and 2 in the presence of K_2_CO_3_ as base (Table 1, entries 8–15). Given the corresponding optimal conditions (Table 1, entry 15), a mixture of PCP (1 mmol), 2 (9 mmol), and K_2_CO_3_ (11.7 mmol) in DMF was heated at 60 °C for 24 hours and product 3b was obtained in yield of 83%. The validation of chemical structures for 3a and 3b was verified through IR and NMR, analyses. The IR spectrum of 3a and 3b showed an absorbance bond for the corresponding terminal acetylenic group, respectively in 2127, and 2123 cm^−1^. The NMR spectrum of these cores was in good agreement with the assigned structures. Appearing a single resonance at *δ* = −164.37 ppm of the ^19^F-NMR spectrum of 3a indicates the displacement of the propargyl alcohol in three positions of the PFP ring. The ^1^H-NMR spectra of 3a and 3b exhibit a high degree of resemblance, except for certain variations observed in their chemical shifts. For example, the ^1^H-NMR spectrum of 3b shows two triplet peaks at *δ* = 3.67, and *δ* = 3.56, with a relative integration ratio of 1 : 2, which are due to the terminal acetylenic hydrogens. Furthermore, the presence of two doublet peaks at *δ* = 4.95, and *δ* = 5.07 ppm are attributed to methylenic hydrogens attached to oxygens. The ^13^C-NMR spectrum of compound 3b showed 9 distinct signals in agreement with the proposed target molecule. Two appeared signals at *δ* = 55.54 and *δ* = 61.29 ppm are related to the methylenic groups, and three signals in the range of 78.05–80.32 ppm were reported due to the presence of the acetylenic carbons. The structure elucidation of the product 3a was also done by single-crystal X-ray analysis (Scheme 2).

**Table 1:** Screening on the reaction of perhalopyridine 1 with propargyl alcohol 2

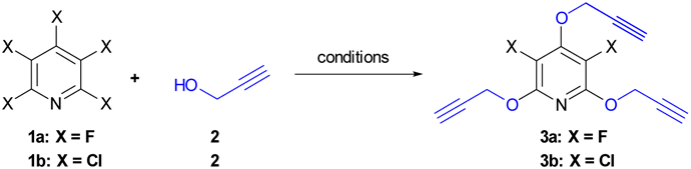							
Entry	1 (mmol)	2 (mmol)	K_2_CO_3_ (mmol)	Solvent	Time (h)	*T* (°C)	Yield^a^ (%)
1	1a (1)	3	3.9	CH_3_CN	48	r.t	50
2	1a (1)	3	3.9	DMF	48	r.t	60
3	1a (1)	3	3.9	DMSO	48	r.t	56
4	1a (1)	3	3.9	THF	48	r.t	28
5	1a (1)	6	7.8	DMF	48	r.t	81
6	1a (1)	9	11.7	DMF	48	r.t	89
**7**	**1a (1)**	**6**	**7.8**	**DMF**	**72**	**r.t.**	**93**
8	1b (1)	3	3.9	CH_3_CN	72	r.t	23
9	1b (1)	3	3.9	DMF	72	r.t	28
10	1b (1)	3	3.9	DMSO	72	r.t	25
11	1b (1)	3	3.9	THF	72	r.t	12
12	1b (1)	3	3.9	DMF	24	60	50
13	1b (1)	3	3.9	DMF	24	80	35
14	1b (1)	6	7.8	DMF	24	60	80
**15**	**1b (1)**	**9**	**11.7**	**DMF**	**24**	**60**	**83**

a Isolated yield.

**Scheme 3:**
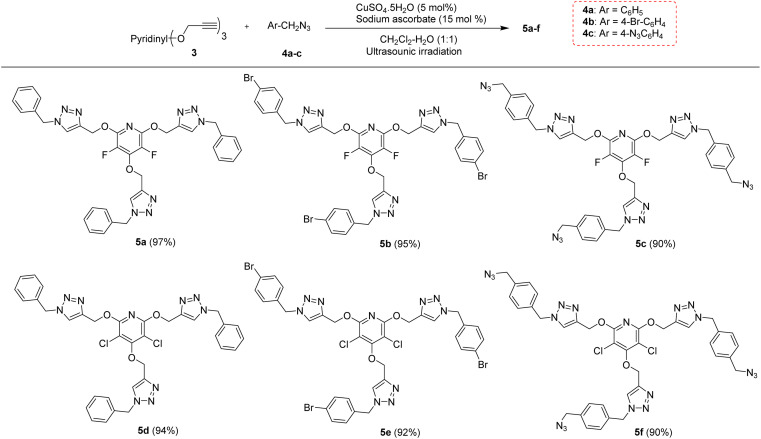
The chemical structures of synthesized tris(1,2,3-triazolyl)-substituted perhalopyridines 5a–f.

The chemical community is interested in preparing poly-triazoles for various applications.^39–43^ On the other hand, incorporation of halogen atom(s) into organic compounds and polymers have been introduced as a powerful method to change or improve the chemical, physical and/or biological activity of a desired molecule. On the basis, it was objected to produce a novel series of poly(1,2,3-triazolyl)-substituted perhalopyridines. First, the required aryl azides 4a–c for producing triazolyl scaffolds were prepared using methods described in the literature.^45^ Then, the reaction between 3a and benzyl azide 4a for the clickable synthesis of compound 5a was chosen as a model and the effect of parameters such as solvent, copper catalysts, and reaction conditions were investigated on the efficiency of the reaction. The results are summarized in Table 2. The reaction of precursor 3a (3.5 mmol) with benzyl azide 4a (11.5 mmol) in the presence of CuSO_4_·5H_2_O (5 mol%) and NaAsc (15 mol%) in CH_3_CN at room temperature afforded a trace amount of 2,4,6-tris((1-benzyl-1*H*-1,2,3-triazol-4-yl)methoxy)-3,5 difluoropyridine 5a after 24 hours. While performing this reaction in DMF solvent led to the production of 5a in 23% yield, utilizing DMF/H_2_O (1 : 1) provided an improvement in the yield of reaction (entries 1 and 2). Accordingly, carrying out the model reaction in several aqueous medium involving THF/H_2_O (1 : 1), *t*-BuOH/H_2_O (1 : 1), and CH_2_Cl_2_/H_2_O (1 : 1) was done and the product 5a was obtained in yields ranging from 43% to 90% (entries 4–7). On this basis, CH_2_Cl_2_/H_2_O (1 : 1) was considered as the best solvent of choice (entry 6). Our next experiments showed that the replacement of CuSO_4_·5H_2_O with other copper catalysts including CuI and Cu(OAc)_2_ reduces the yield of product 5a. Furthermore, the importance of copper catalyst for the construction of triazole scaffolds was demonstrated when no reaction progress was observed in the treatment of 3a with 4a in the absence of copper catalysts (entry 8). As reported in the literature, the necessity of Cu(ii) for this reaction is due to its insitu reduction to Cu(i) required for the Copper-Catalyzed Azide–Alkyne Cycloaddition (CuAAC) reactions. Furthermore, the model reaction was carried out under ultrasonic irradiation, and the sonication procedure was identified to be a powerful alternative to the conventional method. In general, the product 5a was obtained in higher yields and shorter reaction times (entries 1–10). When we increased the amount of copper catalyst, the reaction yield did not change (entry 7). Accordingly, we proceeded to investigate the preparation of tris(1,2,3-triazolyl)-substituted perhalopyridines *via* the reaction of precursors 3 (3.5 mmol) with aryl azides 4a–c (11.5 mmol) in the presence of CuSO_4_·5H_2_O (5 mol%) and NaAsc (15 mol%) in CH_2_Cl_2_/H_2_O (1 : 1) under ultrasonic irradiation. Lukily, treatment of 3a with 1-(azidomethyl)-4-bromobenzene 4b and 1,4-bis(azidomethyl)benzene 4c under optimal conditions afforded tris(1,2,3-triazolyl)-substituted perfluoropyridines 5b and 5c in 95% and 90% yields, respectively. In a similar way, tris(1,2,3-triazolyl)-substituted perchloropyridines 5d–f were successfully obtained through the reaction of precursor 3b and aryl azides 4a–c in yields ranging from 90–94% (Scheme 3). The newly synthesized compounds (5a–f) were characterized based on their infrared spectrum as well as ^1^H, ^13^C, and ^19^F-NMR spectra. For example, the structure of tris-triazolyl 5a was demonstrated by the absence of acetylenic bond in the FT-IR spectrum. The ^1^H-NMR spectrum of 5a shows a single sharp peak at *δ* = 8.28 ppm due to the triazolyl-CH groups, and three singlets at *δ* = 5.40 (s, 2H, CH_2_–N) and *δ* = 5.44 (s, 4H, CH_2_–N), *δ* = 5.59 (s, 6H, CH_2_O) which are related to the three nonequivalent series of methylene bridges. Similarly, the tris-1,2,3-triazole system exhibits a consistent arrangement of aromatic patterns in all instances (refer to the experimental section for more details).

**Table 2:** Optimization of the conditions for the reaction of 3a and 4a to form 1,2,3-triazole 5a^a^

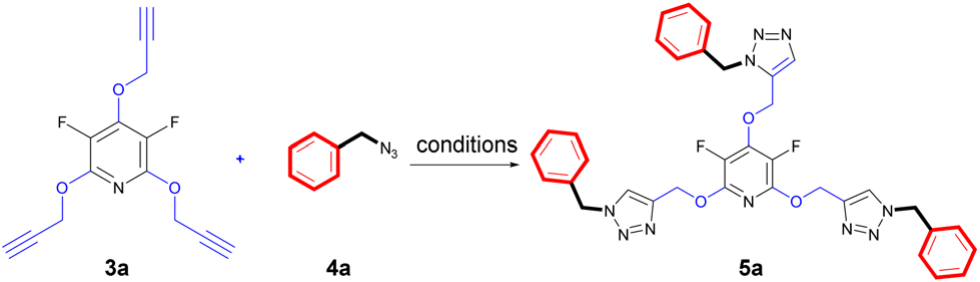				
Entry	Catalyst (mol%)	Solvent	Ambient conditions	US conditions
			Time (h)/yield^b^ (%)	Time (h)/yield^b^ (%)
1	CuSO_4_·5H_2_O (5)	CH_3_CN	24/trace	8/trace
2	CuSO_4_·5H_2_O (5)	DMF	24/23	5/23
3	CuSO_4_·5H_2_O (5)	DMF/H_2_O (1 : 1)	24/36	5/38
4	CuSO_4_·5H_2_O (5)	THF/H_2_O (1 : 1)	24/43	8/48
5	CuSO_4_·5H_2_O (5)	^*t*^BuOH/H_2_O (1 : 1)	24/80	2/88
**6**	**CuSO**_**4**_ **·5H**_**2**_ **O (5)**	**CH**_**2**_ **Cl**_**2**_ **/H**_**2**_ **O (1 : 1)**	**24/90**	**2/97**
7	CuSO_4_·5H_2_O (9)	CH_2_Cl_2_/H_2_O (1 : 1)	24/90	2/97
8	—	CH_2_Cl_2_/H_2_O (1 : 1)	48/n.r.^c^	8/n.r.
9	CuI (5)	CH_2_Cl_2_/H_2_O (1 : 1)	24/23	2/23
10	Cu(OAc)_2_.H_2_O (5)	CH_2_Cl_2_/H_2_O (1 : 1)	24/23	24/23

a All experiments were run using the reaction of precursor 3a (3.5 mmol) with benzyl azide 4a (11.5 mmol) in the presence of NaAsc (15 mol%).

b Isolated yield.

c n.r. = no reaction.

**Scheme 4:**
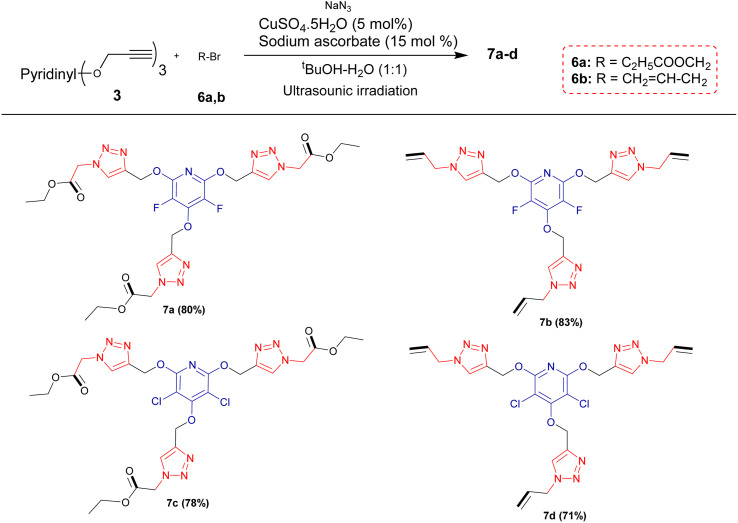
Synthesis of aliphatic tris(1,2,3-triazoles) bearing perhalopyridine moieties.

Encouraged by the above results, it was hypothesized that this methodology could be expanded to generate tris(1,2,3-triazoles) containing perhalopyridine moieties using aliphatic triazides 6. To achieve aliphatic triazoles 7, the reaction of precursor 3a and ethyl 2-azidoacetate 6a was studied to find the optimal reaction conditions. It was found tris(triazole) 7a does not form a satisfactory yield applying the optimal conditions used for the synthesis of tris(1,2,3-triazoles) 5a–f. So, we decided to improve the yield of product 7a through the one-pot reaction of 3a with a small excess amount of sodium azide and aliphatic bromide in the presence of a copper(ii) catalyst with 5 mol% loading under sonication, and the results are shown in Table 3. The corresponding tris(triazole) 7a was obtained in the highest yield *via* the one-pot click reaction 3a (1 mmol), sodium azide (3.6 mmol), ethyl 2-bromoacetate 6a (3 mmol), CuSO_4_·5H_2_O (5 mol%) and sodium ascorbate (15 mol%) in ^*t*^BuOH/H_2_O (1 : 3) under ultrasonic irradiation for 8 hours at 60 °C (Table 3, entry 5). Under these conditions, precursor 3a was also reacted with 3-bromoprop-1-ene 6b for 8 hours and the corresponding aliphatic triazole 7b was isolated in 83% yield (Scheme 3). Similarly, pyridyl core 3b was submitted to the reaction with ethyl 2-bromoacetate 6a and bromoprop-1-ene 6b, and their respective tris(triazoles) 7c and 7d were obtained in yields of 78% and 71%, respectively (Scheme 4). This study highlighted the challenges and outcomes of synthesizing tris(triazoles) with perfluoropyridine moieties, emphasizing the importance of reaction conditions and reactant selection for achieving the desired products, efficiently.

**Table 3:** Investigation of the optimal conditions for the reaction of 3a with ethyl 2-bromoacetate 6a

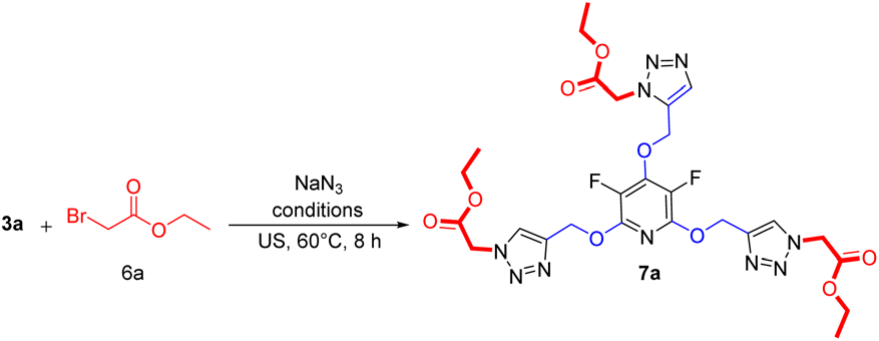			
Entry	Solvent (1 : 3)	Catalyst^a^	Yield^b^ (%)
1	CH_2_Cl_2_/H_2_O (1 : 3)	CuSO_4_·5H_2_O/NaAsc	70
2	CH_2_Cl_2_/H_2_O (1 : 3)	Cu(OAc)_2_·H_2_O/NaAsc	68
3	CH_2_Cl_2_/H_2_O (1 : 3)	CuSO_4_·5H_2_O/NaAsc	73
4	^*t*^BuOH/H_2_O (1 : 3)	Cu(OAc)_2_·H_2_O/NaAsc/1,10-Phen.H_2_O	78
**5**	^***t***^ **BuOH/H**_**2**_ **O (1 : 3)**	**CuSO**_**4**_ **·5H**_**2**_ **O/NaAsc**	**80**

a CuSO_4_·5H_2_O and Cu(OAc)_2_.H_2_O at 5 mol%, along with NaAsc 15 mol%.

b Isolated yield.

### 2.2. Synthesis of 2-((1-benzyl-1*H*-1,2,3-triazol-4-yl)methoxy)-3,4,5,6-tetrachloropyridine derivatives

**Scheme 5:**
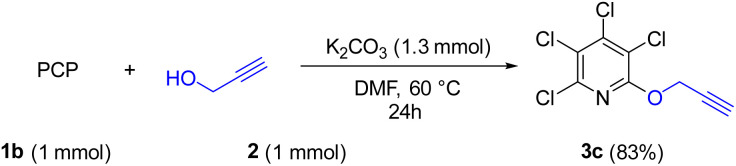
Site-selective synthesis of 2,3,4,5-tetrachloro-6-(prop-2-yn-1-yloxy)pyridine 3c.

**Scheme 6:**
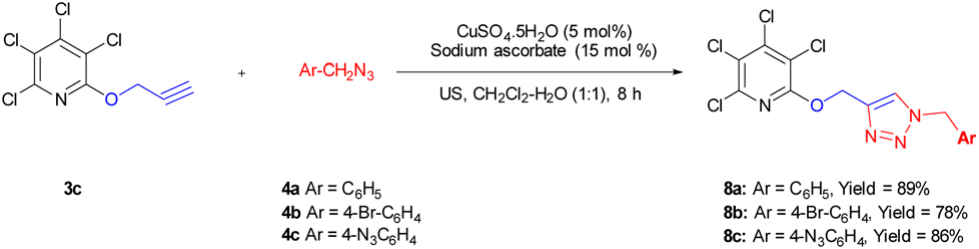
Synthesis of ((1,2,3-triazol-4-yl)methoxy)-3,4,5,6-tetrachloropyridine 8a–c derivatives. Reaction conditions: 3c (1 mmol) was, then, aryl azides 4a–c (1.2 mmol), CuSO_4_·5H_2_O (5 mol%), NaAsc (15 mol%) in CH_2_Cl_2_/H_2_O (1 : 1) under ultrasonic irradiation at 60 °C for 8 hours.

**Scheme 7:**
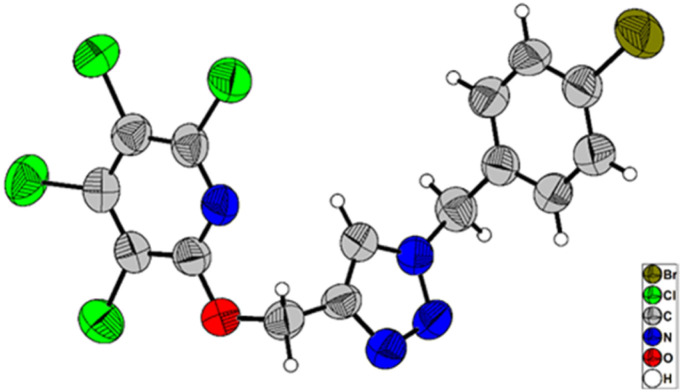
X-ray crystal structure of 8b.

Due to the significance of site-selective nucleophilic substitution reactions involving pentachloropyridine,^44^ particularly position 4 as the primary preference against nucleophilic attacks, herein, we describe, for the first time, the direct execution of nucleophilic substitution at the C-2 position of pentachloropyridine without the utilization of any catalyst. In this respect, we were interested in synthesizing 2,3,4,5-tetrachloro-6-(prop-2-yn-1-yloxy)pyridine 3c through regioselective nucleophilic substitution reaction of pentachloropyridine with propargyl alcohol under those conditions reported in section 2.1 but using PCP and propargyl alcohol in a 1 : 1 molar ratio. Accordingly, the reaction of PCP (1 mmol) with 2 (1 mmol) in the presence of K_2_CO_3_ (1.3 mmol) in DMF at 60 °C for 24 hours afforded product 3c in 83% yield (Scheme 5). The derived product 3c (1 mmol) was, then, submitted to the reactions with aryl azides 4a–c (1.2 mmol) in the presence of CuSO_4_·5H_2_O (5 mol%) and NaAsc (15 mol%) in CH_2_Cl_2_/H_2_O (1 : 1) under ultrasonic irradiation at 60 °C for 8 hours and their respective ((1,2,3-triazol-4-yl)methoxy)-3,4,5,6-tetrachloropyridines 8a–c were synthesized in yields of 78–89% (Scheme 6). Furthermore, the structure of 8b was confirmed by X-ray crystallographic data (Scheme 7). The structure elucidation of 3a was first performed by infrared and NMR spectroscopy. In the FT-IR spectrum of product 3c, the absorption band of the acetylenic group is observed at 2132 cm^−1^. The ^1^H-NMR spectrum of 3c shows two distinct peaks at *δ* = 3.65, and *δ* = 5.05 ppm which are attributed to the acetylenic and methylenic hydrogens, respectively. In the ^13^C-NMR, five signals appearing in the region *δ* = 117.04–155.94 ppm are related to the fourth type carbons of the pyridine ring, and three distinct signals in the *δ* = 79.19, *δ* = 78.36, and *δ* = 56.46 ppm can be attributed to the presence of acetylenic and methylenic groups, respectively.

### 2.3. Synthesis of biaryl-embedded perhalopyridine-based poly(1,2,3-triazoles)

**Scheme 8:**
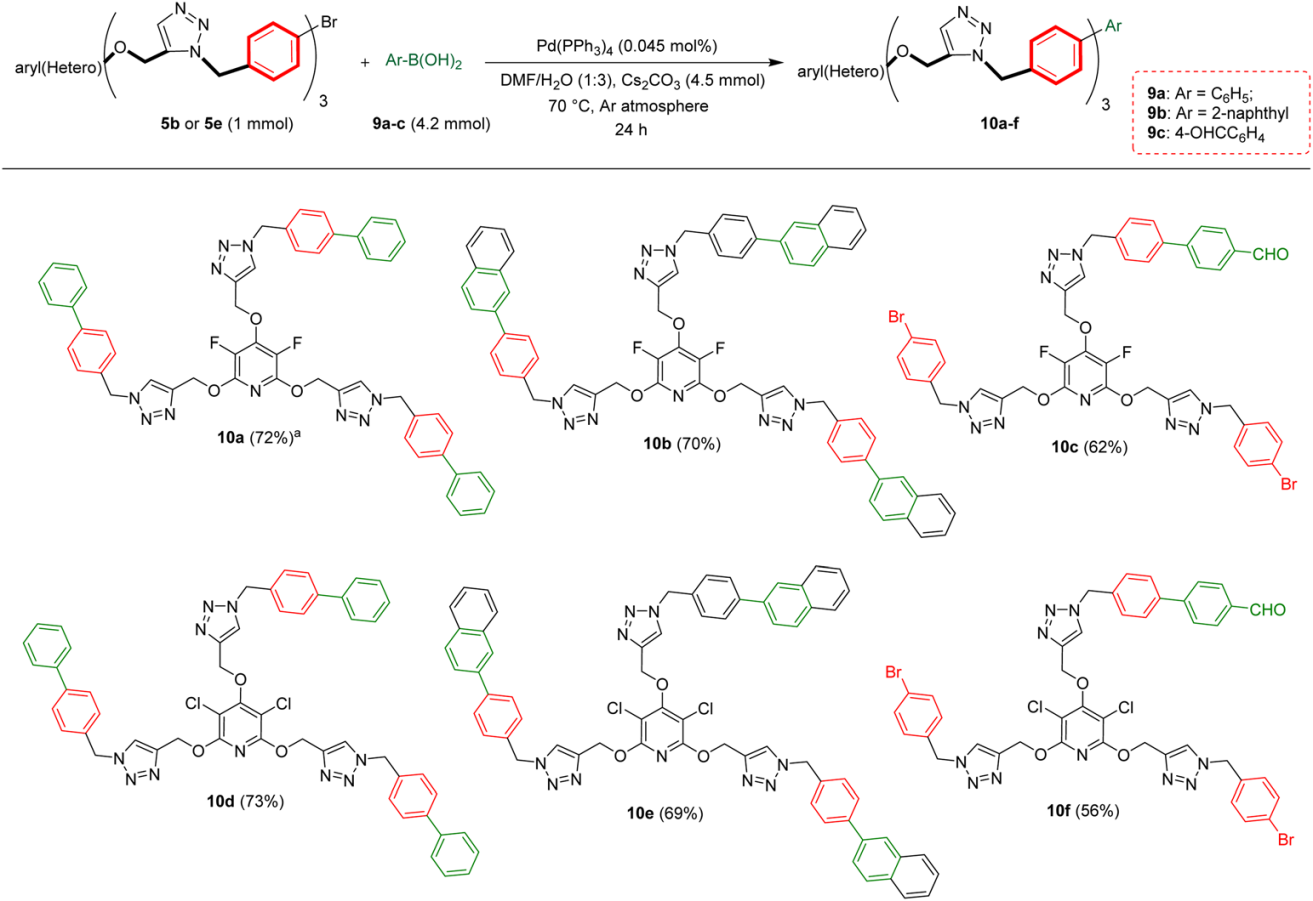
Pd(PPh_3_)_4_ catalyzed Suzuki–Miyaura coupling reaction of aryl bromides 5b and 5e with arylboronic acids 9a–c. ^*a*^Isolated yield.

Biaryls are an integral part of various natural products^46^ and biologically active compounds.^47^ They are also fundamental building blocks^46*d*^ in organic synthesis and material chemistry. Biaryl scaffolds are traditionally formed *via* the transition-metal catalyzed Suzuki–Miyaura coupling (SMC) reactions of aryl boronic acids with aryl halides.^48^ After the successful synthesis of tris(1,2,3-triazoles) 5b or 5e, we aimed to synthesis their respective biaryl-embedded 1,2,3-triazoles. Therefore, the Suzuki–Miyaura cross-coupling (SMC) reaction of tris-triazole 5b and phenylboronic acid 9a in the presence of Pd(PPh_3_)_4_ was chosen as a model, and the effect of different variants including solvent, base, molar ratios of substrates and catalyst loading was explored under thermal conditions. The results are listed in Table 4. In the best conditions, the coupling product 10a was obtained in yield of 72% through stirring a mixture of 5a (1 mmol), phenylboronic acid 9a (3.8 mmol), Cs_2_CO_3_ (4.5 mmol), and [Pd(PPh_3_)_4_] (0.045 mol%) in DMF/H_2_O (1 : 3) at 70 °C for 24 hours. Under these conditions, tris(1,2,3-triazolyl)-substituted perfluoropyridine 5b was also reacted with 2-naphthylboronic acid 9b to achieve the corresponding biaryl-embedded perfluoropyridine-based poly(1,2,3-triazoles) 10b in 70% yield. However, in the treatment of 5b with 4-formylphenylboronic acid 9c under optimized conditions, the coupling reaction took place at only one position, and product 10c gained in yield of 62% (Scheme 7). In a similar way, the reaction of tris(1,2,3-triazolyl)-substituted perchloropyridine 5e with arylboronic acids 9a–c afforded their respective biaryl-embedded perchloropyridine-based poly(1,2,3-triazoles) 10d and 10e in good yields (Scheme 8). As similar to 5b, aryl bromide 5e was coupled with 4-formylphenylboronic acid 9c at only one position under optimal conditions, and the corresponding product 10f was isolated in a yield of 56%. The structures of coupling products were confirmed using FT-IR and NMR analyses.

**Table 4:** Screening of the reaction conditions for the SMC reaction of compound 5b with phenylboronic acid 9a^a^

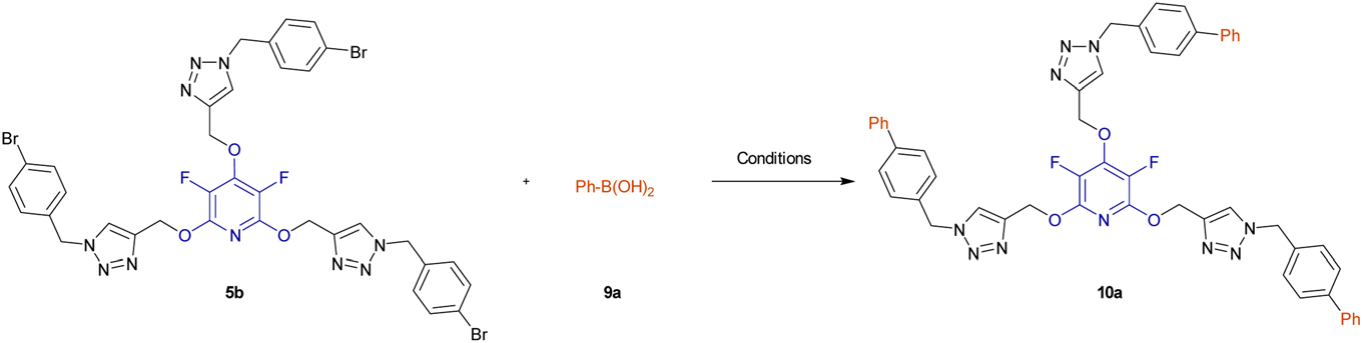						
Entry	Base	5b : 9a: base (mmol)	Solvent (1 : 3)	Pd(PPh_3_)_4_ (mol%)	Time (h)	Yield^b^ (%)
1	Et_3_N	1 : 3.8 : 4.5	DMF/H_2_O	0.018	24	38
2	Et_3_N	1 : 3.8 : 4.5	THF/H_2_O	0.018	24	25
3	K_3_PO_4_	1 : 3.8 : 4.5	DMF/H_2_O	0.018	24	51
4	K_2_CO_3_	1 : 3.8 : 4.5	DMF/H_2_O	0.018	24	53
5	K_2_CO_3_	1 : 4.2 : 4.5	DMF/H_2_O	0.045	24	65
**6**	**Cs**_**2**_ **CO**_**3**_	**1 : 3.8 : 4.5**	**DMF/H**_**2**_ **O**	**0.045**	**24**	**72**
7	Cs_2_CO_3_	1 : 4.2 : 4.5	DMF/H_2_O	—	24	—

a All the reactions were run in 4 mL of solvent at 70 °C.

b Isolated yield.

## 3. Experimental details

### 3.1. General information

All chemicals purchased from Merck chemical company and used without further purification unless specified. The advancement of the reactions was tracked through TLC analysis on polyester sheets coated with silica gel-60 and fluorescent indicator (F-252) obtained from Merck. Melting points were conducted on a Stuart SMP2 apparatus and left uncorrected. IR spectra were recorded on a Nicolet-Impact 400D spectrophotometer using KBr pellets. ^1^H-, ^13^C-, and ^19^F-NMR spectra were recorded on a Bruker Ultrashield-400 NMR spectrometer using DMSO-d_6_ as a solvent, while those for ^19^F were reported in ppm relative to CFCl_3_ as the standard. The ultrasonic device used was an UP 400 S instrument from Dr Hielscher GmbH. An S3 immersion horn emitting 24 kHz ultrasound at intensity levels tunable to maximum sonic power density of 460 W cm^2^ was used. Sonication was carried out at 100% (maximum amplitude 210 lm). A 3 mm long sonotrode (maximum immerse depth of 90 mm) was immersed directly into the reaction mixture.

### 3.2. Synthesis of 3,5-difluoro-2,4,6-tris(prop-2-yn-1-yloxy)pyridine 3a

In a 25 mL round-bottomed flask, a mixture of propargyl alcohol (6 mmol), K_2_CO_3_ (7.8 mmol), and DMF (5 mL) was stirred at room temperature for 30 minutes. Next, PFP (1 mmol) was added and the resulting mixture was stirred at room temperature for 72 hours. Then, the reaction mixture was poured into 10 mL water and extracted with ethyl acetate (2 × 30 mL). The organic phase was dried over MgSO_4,_ and the solvent evaporated. The pure product 3a was obtained in 93% yield after washing with hexane.

Light yellow solid. Yield 93%. MP 118–120 °C; IR (KBr) *ν̃* 3283, 2943, 2127, 1632 cm^−1^. ^1^H NMR (500 MHz, DMSO-d_6_) *δ* 5.08 (d, *J* = 2.4 Hz, 2H, CH_2_–O), 5.02 (d, 4H, *J* = 2.4 Hz, CH_2_–O), 3.72 (t, 1H, *J* = 4.9 Hz, acetylene-H), 3.54 (t, *J* = 4.9 Hz, 2H, acetylene-H) ppm. ^13^C-NMR (126 MHz, DMSO-d_6_) *δ* 144.16 (dm, ^2^*J*_CF_ = 10.90 Hz, C2,6-py), 143.45 (d, ^2^*J*_CF_ = 10.40 Hz, C4-py), 134.24 (d, ^1^*J*_CF_ = 248.9 Hz, C3,5-py), 80.53 (acetylene-CH), 79.20 (acetylene-C), 78.42 (acetylene-CH), 78.13 (acetylene-C), 61.55 (CH_2_–O), 54.81 (CH_2_–O) ppm. ^19^F NMR (470 MHz, DMSO-d_6_) *δ* −164.37 (2F, F3,5-py).

### 3.3. Synthesis of 3,5-dichloro-2,4,6-tris-prop-2-ynyloxy-pyridine 3b

In a 25 mL round-bottomed flask, a mixture of propargyl alcohol (9 mmol), K_2_CO_3_ (11.7 mmol), and DMF (5 mL) was stirred at room temperature for 30 minutes. Next, PCP (1 mmol) was added and the resulting mixture was stirred at 60 °C for 24 hours. Then, the reaction mixture was poured into 10 mL water and extracted with ethyl acetate (2 × 30 mL). The organic phase was dried over MgSO_4,_ and the solvent evaporated. The pure product 3b was obtained in 83% yield after washing with hexane.

Light brown solid. Yield 83%. MP 121–124 °C. IR (KBr) *ν̃* 3277, 2513, 2123, 1796 cm^−1^. ^1^H NMR (500 MHz, DMSO-d_6_) *δ* 5.07 (d, 4H, *J* = 2.4 Hz, CH_2_–O), 4.95 (d, 2H, *J* = 2.4 Hz, CH_2_–O), 3.67 (t, 1H, *J* = 5 Hz, acetylene-H), 3.56 (t, 2H, *J* = 5 Hz, acetylene-H) ppm. ^13^C NMR (126 MHz, DMSO-d_6_) *δ* 160.30, 155.04, 104.19, 80.32 (acetylene-CH), 79.07 (acetylene-C), 78.53 (acetylene-CH), 78.05 (acetylene-C), 61.29 (CH_2_–O), 55.54 (CH_2_–O) ppm.

### 3.4. General procedure for the synthesis of aryl azides 4a–c (ref. 45)

Aryl azides 4a–c were prepared according to the reported method in the literature.^41^ To a stirred solution of NaN_3_ (5.5 mmol) in DMSO (5.0 mL) was added benzyl bromide (5.0 mmol). The resulting mixture was stirred at 80 °C overnight. The reaction mixture was then cooled to room temperature and diluted with water (15 mL), it was extracted with diethyl ether (3 × 10 mL) and washed with brine, dried over MgSO_4,_ and concentrated under a vacuum to give the products 4a–c as colorless liquids in quantitative yields. They were used directly without further purification.

### 3.5. General procedure for the synthesis of tris(1,2,3-triazoles) 5a–f under ultrasonic irradiation

Precursors 3a,b (3.5 mmol) was dissolved in CH_2_Cl_2_/H_2_O (5 mL, 1 : 1), aryl azides 4a–c (11.5 mmol), CuSO_4_·5H_2_O (5 mol%) and NaAsc (15 mol%) were added and the resulting mixture was sonicated at 60 °C for 8 hours. The completion of the reaction was controlled by TLC until the consumption of precursors 3. Then, H_2_O (5 mL) was added to the mixture, and the precipitate was collected by filtration, washed thoroughly with H_2_O, and CH_2_Cl_2_, and dried under vacuum. The crude was purified by column chromatography using CH_2_Cl_2_/MeOH (95/5) as eluent to obtain products 5a–f.

#### 2,4,6-Tris((1-benzyl-1*H*-1,2,3-triazol-4-yl)methoxy)-3,5-difluoropyridine 5a

White solid. Yield 97%. MP 165–168 °C. IR (KBr) *ν̃* 3726, 3630, 3448, 3137, 3087, 3032, 2956 cm^−1^. ^1^H NMR (500 MHz, DMSO-d_6_): *δ* 8.29 (s, 3H, tetrazole-H), 7.26–7.40 (m, 15H, Ar–H), 5.59 (s, 6H, CH_2_O), 5.44 (s, 4H, CH_2_–N), 5.40 (s, 2H, CH_2_–N). ^13^C NMR (126 MHz, DMSO-d_6_): *δ* 144.90 (dm, ^2^*J*_CF_ = 10.01 Hz, C2,6-py), 143.94 (d, ^2^*J*_CF_ = 8.67 Hz, C4-py), 143.03 (triazole-C), 142.38 (triazole-C), 136.42 (Ar–C), 134.0 (d, ^1^*J*_CF_ = 247.5 Hz, C3,5-py), 129.21 (Ar–CH), 129.18 (Ar–CH), 128.61 (Ar–CH), 128.56 (Ar–CH), 128.46 (Ar–CH), 128.17 (Ar–CH), 125.9 (alkene-CH), 125.4 (alkene-CH), 66.67 (CH_2_–O), 60.03 (CH_2_–O), 53.32 (CH_2_–N), 53.26 (CH_2_–N) ppm. ^19^F NMR (470 MHz, DMSO-d_6_) *δ* −164.61 (2F, F3,5-py).

#### 2,4,6-Tris((1-(4-bromobenzyl)-1*H*-1,2,3-triazol-4-yl)methoxy)-3,5-difluoropyridine 5b

White solid. Yield 95%. MP 184–186 °C; IR (KBr) *ν̃* 3725, 3431, 1626, 1491 cm^−1^. ^1^H NMR (500 MHz, DMSO-d_6_) *δ* 8–30 (s, 3H, tetrazole-H), 7.55 (d, *J* = 8.3 Hz, 6H, Ar–H), 7.27 (d, *J* = 8.4 Hz, 4H), 7.20 (d, *J* = 8.5 Hz, 2H), 5.58 (s, 6, CH_2_O), 5.46 (s, 4H, CH_2_–N), 5.40 (s, 2H, CH_2_–N) ppm. ^13^C NMR (125 MHz, DMSO-d_6_): 144.91 (m, C2,6-py), 144.01 (m, C4-py), 143.08, 142.43, 135.78, 135.01 (d, ^1^*J*_CF_ = 247.5 Hz, C3,5-py), 133.03, 132.12, 130.66, 130.49, 125.84 (alkene-CH), 125.45, 121.90, 121.87 (alkene-CH), 66.65 (CH_2_–O), 60.01 (CH_2_–O), 52.58 (CH_2_–N), 52.55 (CH_2_–N) ppm. ^19^F NMR (470 MHz, DMSO-d_6_) *δ* −164.62 (2F, F3,5-py).

#### 2,4,6-Tris((1-(4-(azidomethyl)benzyl)-1*H*-1,2,3-triazol-4-yl)methoxy)-3,5-difluoropyridine 5c

White solid. Yield 90%. MP 200 °C (dec); IR (KBr) *ν̃* 3726, 3630, 3448, 3137, 2927, 2099, 1626 cm^−1^. ^1^H NMR (500 MHz, DMSO-d_6_) *δ* 8–26 (s, 3H, tetrazole-H), 7.33–7.35 (m, 12H, Ar–H), 5.60 (s, 6, CH_2_O), 5.45 (s, 4H, CH_2_–N), 5.40 (s, 2H, CH_2_–N), 4.46 (s, 2H, CH_2_–N_3_), 4.41 (s, 4H, CH_2_–N_3_) ppm. ^13^C NMR (126 MHz, DMSO-d_6_) 162.71, 143.87 (m), 142.99, 142.37 (m), 142.33, 136.28, 136.10, 135.99 134.18 (d, ^1^*J*_CF_ = 203.75 Hz, C3,5-py), 129.17, 128.73, 128.50, 125.39 (alkene-CH), 66.61 (CH_2_–O), 60.01 (CH_2_–O), 53.72 (CH_2_–N), 52.92 (CH_2_–N), 36.19, 31.20 ppm. ^19^F NMR (470 MHz, DMSO-d_6_) *δ* −164.62 (2F, F3,5-py).

#### 2,4,6-Tris((1-benzyl-1*H*-1,2,3-triazol-4-yl)methoxy)-3,5-dichloropyridine 5d

White solid. Yield 94%. MP 175–177 °C; IR (KBr) *ν̃* 3726, 3449, 3132, 3066, 3032, 2953, 1576 cm^−1^. ^1^H NMR (500 MHz, DMSO-d_6_) *δ* 8.33–8.35 (3H, tetrazole-H), 7.27–7.36 (m, 15H, Ar–H), 5.60 (6, CH_2_O), 5.50 (4H, CH_2_–N), 5.22 (2H, CH_2_–N) ppm. ^13^C-NMR (126 MHz, DMSO-d_6_) *δ* 160.79, 155.75, 142.89, 136.45, 136.40,129.20 (Ar–CH), 129.15 (Ar–CH), 128.87 (Ar–CH), 128.60 (Ar–CH), 128.52 (Ar–CH), 128.43 (Ar–CH), 128.15 (alkene-CH), 125.99, 125.42, 103.67 (alkene–CH), 66.57 (CH_2_–O), 60.77 (CH_2_–O), 53.31 (CH_2_–N), 53.23 (CH_2_–N) ppm.

#### 2,4,6-Tris((1-(4-bromobenzyl)-1*H*-1,2,3-triazol-4-yl)methoxy)-3,5-dichloropyridine 5e

Light brown solid. Yield 92%. MP 200–203 °C; IR (KBr) *ν̃* 3841, 3725, 3448, 3061, 2953 cm^−1^. ^1^H NMR (500 MHz, DMSO-d_6_): *δ* 8–30 (s, 3H, tetrazole-H), 7.51–7.55 (m, 6H, Ar–H), 7.19–7.29 (m, 6H, Ar–H), 5.58 (s, 6, CH_2_O), 5.51 (s, 4H, CH_2_–N), 5.21 (s, 2H, CH_2_–N) ppm. ^13^C-NMR (126 MHz, DMSO-d_6_) *δ* 155.74, 142.93, 135.76, 132.12 (Ar–CH), 132.07 (Ar–CH), 130.68 (Ar–CH), 130.46 (Ar–CH), 126.01 (Ar–CH), 125.45 (Ar–CH), 121.90 (alkene-CH), 103.69 (alkene-CH), 66.56 (CH_2_–O), 60.75 (CH_2_–O), 52.58 (CH_2_–N), 52.54 (CH_2_–N) ppm.

#### 2,4,6-Tris((1-(4-(azidomethyl)benzyl)-1*H*-1,2,3-triazol-4-yl)methoxy)-3,5-dichloropyridine 5f

Light brown solid. Yield 90%. MP 250 °C (dec); IR (KBr*) ν̃* 3726, 3424, 3136, 2949, 2098, 1561. ^1^H NMR (500 MHz, DMSO-d_6_) *δ* 8.33 (s, 3H, tetrazole-H), 7.22–7.43 (m, 12H, Ar–H), 5.61–5.48 (m, 12H, CH_2_O, CH_2_–N), 5.22 (s, 2H, CH_2_N_3_), 4.41 (s, 4H, CH_2_N_3_) ppm. ^13^C-NMR (126 MHz, DMSO-d_6_) *δ* 135.76, 132.16, 132.11 (Ar–CH), 131.89, 130.60 (Ar–CH), 130.48, 129.07 (Ar–CH), 128.33 (Ar–CH), 127.80 (Ar–CH), 127.55, 125.37, 125.36 (Ar–CH), 121.87 (alkene-CH), 115.68 (alkene-CH), 67.90 (CH_2_–O), 60.03 (CH_2_–O), 52.56 (CH_2_–N), 52.55 (CH_2_–N), 38.58, 30.27 ppm.

### 3.6. General procedure for the synthesis of tris(1,2,3-triazoles) 7a–d under ultrasonic irradiation

To a mixture of precursors 3a,b (1 mmol), aliphatic bromides 6a,b (3 mmol), and NaN_3_ (3.6 mmol) in ^*t*^BuOH/H_2_O (8 mL, 1 : 3 v/v), was added CuSO_4_·5H_2_O (5 mol%) and AscNa (15 mol%). The mixture was then sonicated at 60 °C for the appropriate time. Finally, the crude product was purified by column chromatography using CH_2_Cl_2_/MeOH (95 : 5) as eluent to obtain the pure products 7a–d.

#### Triethyl 2,2′,2′′-((((3,5-difluoropyridine-2,4,6-triyl)tris(oxy))tris(methylene))tris(1*H*-1,2,3-triazole-4,1-diyl))triacetate 7a

White solid. Yield 80%. MP 153–155 °C; IR (KBr) *ν̃* 3726, 3146, 2964, 2100, 1743, 1626 cm^−1^. ^1^H-NMR (500 MHz, DMSO-d_6_): *δ* 8.26 (s, 3H, tetrazole-H), 5.52 (s, 4H, CH_2_O), 5.47 (s, 2H, CH_2_–O), 5.39 (s, 6H, CH_2_–N), 4.14–4.19 (q, 6H, *J* = 7.9 Hz, CH_2_), 1.20 (t, 9H, *J* = 7.6 Hz, CH_3_) ppm. ^13^C NMR (126 MHz, DMSO-d_6_) *δ* 167.56, 144.90 (dm, ^2^*J*_CF_ = 11.25 Hz, C2,6-py), 144.05, 143.96, 142.76, 142.12, 133.96 (d, ^1^*J*_CF_ = 245 Hz, C3,5-py), 126.99, 126.68, 66.57, 61.93, 60.00, 50.86, 14.36 ppm. ^19^F NMR (470 MHz, DMSO-d_6_) *δ* −164.83 (2F, F3,5-py).

#### 2,4,6-Tris((1-allyl-1*H*-1,2,3-triazol-4-yl)methoxy)-3,5-difluoropyridine 7b

Light brown liquid. Yield 83%. IR (KBr) *ν̃* 3726, 3375, 2926, 2139, 1657, 1625. ^1^H NMR (500 MHz, DMSO-d_6_) *δ* 8.21 (s, 3H, tetrazole-H), 5.95–6.09 (m, 3H, CH), 5.51 (s, 4H, CH_2_O), 5.44 (s, 2H, CH_2_–O), 5.16–5.25 (ddt, 6H, CH=), 5.01–5.05 (m, 6H, CH_2_–N) ppm. ^13^C-NMR (126 MHz, DMSO-d_6_) *δ* 144.94 (dm, ^2^*J*_CF_ = 10 Hz, C2,6-py), 144.53, 144.02 (m), 143.18, 142.89, 142.24, 134.21 (d, ^1^*J*_CF_ = 208.5 Hz, C3,5-py), 125.50, 125.11, 119.25, 66.64 (CH_2_–O), 60.01 (CH_2_–O), 55.20 (CH_2_–N), 52.18 (CH_2_–N) ppm. ^19^F NMR (470 MHz, DMSO-d_6_) *δ* −164.80 (2F, F3,5-py).

#### Triethyl 2,2′,2′′-((((3,5-dichloropyridine-2,4,6-triyl)tris(oxy))tris(methylene))tris(1H-1,2,3-triazole-4,1-diyl))triacetate 7c

White solid. Yield 78%. MP 168–170 °C; IR (KBr) *ν̃* 2923, 2855, 1638, 1420 cm^−1^. ^1^H-NMR (500 MHz, DMSO-d_6_): *δ* 8.33 (s, 3H, tetrazole-H), 5.62 (s, 4H, CH_2_–O), 5.45 (s, 6H, CH_2_–N), 5.30 (s, 2H, CH_2_–O), 4.14–4.24 (q, 6H, *J* = 7.8 Hz, CH_2_), 1.23 (t, 9H, *J* = 6.1 Hz CH_3_) ppm. ^13^C NMR (126 MHz, DMSO-d_6_) *δ* 167.69, 167.57, 160.94, 155.79, 142.66, 142.13, 127.24, 126.84, 103.69, 66.63, 61.99, 60.78, 50.90, 14.41 ppm.

#### 2,4,6-Tris((1-allyl-1*H*-1,2,3-triazol-4-yl)methoxy)-3,5-difluoropyridine 7d

Light brown liquid. Yield 71%. IR (KBr) *ν̃* 3652, 3418, 2923, 1720, 1578. ^1^H NMR (500 MHz, DMSO-d_6_) *δ* 8.21–8.27 (s, 3H, tetrazole-H), 5.98–6.09 (m, 3H, CH), 5.55 (s, 4H, CH_2_O), 5.50 (s, 2H, CH_2_–O), 5.20–5.26 (ddt, 6H, CH=), 5.00–5.04 (m, 6H, CH_2_–N) ppm. ^13^C-NMR (126 MHz, DMSO-d_6_): *δ* 155.79, 142.73, 142.71, 133.13, 125.74, 125.21 (Ar–C), 124.46, 119.31, 118.94, 103.73, 66.70 (CH_2_–O), 60.80 (CH_2_–O), 52.17 (CH_2_–N), 52.06 (CH_2_–N) ppm.

### 3.7. Synthesis of 2,3,4,5-tetrachloro-6-(prop-2-yn-1-yloxy)pyridine 3c

In a 25 mL round-bottomed flask, a mixture of propargyl alcohol (1 mmol), K_2_CO_3_ (1.3 mmol), and DMF (2 mL) was stirred at room temperature for 30 minutes. Next, PCP (1 mmol) was added and the resulting mixture was stirred at 60 °C for 24 hours. Then, the reaction mixture was poured into 10 mL water and extracted with ethyl acetate (2 × 30 mL). The organic phase was dried over MgSO_4,_ and the solvent evaporated. The pure product 3c was obtained in 83% yield after washing with hexane.

Brown solid. Yield 83%. IR (KBr*) ν̃* 3394, 3143, 2919, 2132, 1627. ^1^H NMR (500 MHz, DMSO-d_6_) *δ* 5.05 (d, 2H, CH_2_–O), 3.65 (t, 1H, acetylene-H) ppm. ^13^C-NMR (125 MHz, DMSO-d_6_) *δ* 155.94, 143.79, 143.54, 117.04, 79.19 (acetylene-C), 78.36 (acetylene-CH), 56.46 (CH_2_–O) ppm.

### 3.8. Synthesis of ((1,2,3-triazol-4-yl)methoxy)-3,4,5,6-tetrachloropyridine 8a–c derivatives

Precursors 3c (1 mmol) were dissolved in CH_2_Cl_2_/H_2_O (5 mL, 1 : 1). Aryl azides 4a–c (1.2 mmol), CuSO_4_·5H_2_O (5 mol%), and NaAsc (15 mol%) were added and the resulting mixture was sonicated at 60 °C for 8 hours. The completion of the reaction was controlled by TLC until the consumption of precursors 3c. Then, H_2_O (5 mL) was added to the mixture, and the precipitate was collected by filtration, washed thoroughly with H_2_O, and CH_2_Cl_2_, and dried under vacuum. The crude was purified by column chromatography using EtOAc/Hexane (50/20) as eluent to obtain products 8a–c.

#### 2-((1-Benzyl-1*H*-1,2,3-triazol-4-yl)methoxy)-3,4,5,6-tetrachloropyridine 8a

White solid. Yield 89%. MP 135–137 °C; IR (KBr) *ν̃* 3416, 2924, 2853, 1618, 1497 cm^−1^. ^1^H-NMR (500 MHz, DMSO-d_6_) *δ* 8.37 (s, 1H, tetrazole-H), 7.28–7.36 (m, 5H, Ar-CH), 5.63 (s, 2H, CH_2_–O), 5.33 (s, 2H, CH_2_–N) ppm. ^13^C-NMR (126 MHz, DMSO-d_6_) *δ* 144.43, 143.35, 135.81, 133.21, 132.11, 131.28, 130.62, 125.31, 121.88, 59.68 (CH_2_–O), 52.56 (CH_2_–N) ppm.

#### 2-((1-(4-Bromobenzyl)-1*H*-1,2,3-triazol-4-yl)methoxy)-3,4,5,6-tetrachloropyridine 8b

White crystal. Yield 78%. MP 146–147 °C; IR (KBr) *ν̃* 3726, 3423, 2929, 2513, 1796, 1721, 1639 cm^−1^. ^1^H-NMR (500 MHz, CDCl_3_) *δ* 7.67 (s, 1H, tetrazole-H), 7.52–7.55 (d, 2H, Ar–H), 7.18–7.20 (d, 2H, Ar–CH), 5.56 (s, 2H, CH_2_–N), 5.52 (s, 2H, CH_2_–O) ppm. ^13^C-NMR (125 MHz, CDCl_3_) *δ* 156.20, 144.09, 143.75, 142.97, 133.38, 132.35, 129.75, 123.95, 123.07, 122.40, 117.30, 61.43 (CH_2_–O), 53.56 (CH_2_–N) ppm.

#### 2-((1-(4-Bromobenzyl)-1*H*-1,2,3-triazol-4-yl)methoxy)-3,4,5,6-tetrachloropyridine 8c

Light brown solid. Yield 86%. MP 159–162 °C; IR (KBr) *ν̃* 3726, 3416, 2924, 2853, 1629, 1497 cm^−1^. ^1^H-NMR (500 MHz, DMSO-d_6_) *δ* 8.36 (1H, tetrazole-H), 6.90–7.64 (m, 4H, Ar–H), 5.60–5.70 (m, 2H, CH_2_–O), 5.31–5.45 (m, 2H, CH_2_–N), 3.55 (s, 2H, CH_2_–N_3_), 5.28–5.48 (m, 2H, CH_2_–N) ppm. ^13^C-NMR (125 MHz, DMSO-d_6_): *δ* 143.57, 141.96, 136.25, 132.17, 132.04, 129.29, 129.16, 128.87, 125.87, 61.83 (CH_2_–O), 53.62 (CH_2_–N) ppm.

### 3.9. General procedure for SMC reactions of triazolyl bromides 5b,e with arylboronic acids 9a–c under thermal condition

A mixture of triazolyl bromides 5a,e (1 mmol), phenylboronic acid 9a (3.8 mmol), Cs_2_CO_3_ (4.5 mmol), and [Pd(PPh_3_)_4_] (0.045 mol%) in DMF/H_2_O (1 : 3) was stirred at 70 °C under argon atmosphere for 24 hours. The catalyst was separated by filtration and the crude product was purified by column chromatography on silica gel using CH_2_Cl_2_–MeOH (9 : 1) as eluent to obtain the pure coupling products 10a–f.

#### 2,4,6-Tris((1-([1,1′-biphenyl]-4-ylmethyl)-1*H*-1,2,3-triazol-4-yl)methoxy)-3,5-difluoropyridine 10a

White solid. Yield 72%. MP 203–207 °C; IR (KBr) *ν̃* 3062, 2957, 2929, 1723, 1626 cm^−1^. ^1^H-NMR (500 MHz, DMSO-d_6_) *δ* 8.17 (s, 3H, tetrazole-H), 7.58–7.66 (m, 12H, Ar–H), 7.33–7.43 (m, 10H), 7.19–7.27 (m, 4H), 5.55–5.64 (m, 6H, CH_2_O), 5.40–5.48 (m, 4H, CH_2_–N), 5.25 (s, 2H, CH_2_–N) ppm. ^13^C NMR (126 MHz, DMSO-d_6_) *δ* 167.40, 144.00 (m), 143.05, 142.43, 142.36 (m), 140.50, 140.00, 135.77, 135.51, 134.04 (d, ^1^*J*_CF_ = 247.5 Hz, C3,5-py) 132.20, 132.11 (d, ^2^*J*_CF_ = 8.67 Hz, C4-py), 131.98, 130.65, 130.57 (Ar–C), 130.49, 129.35 (Ar–CH), 129.08 (Ar–CH), 129.03 (Ar–CH), 128.97 (Ar–CH), 128.89 (Ar–CH), 128.02 (Ar–CH), 127.84, 127.49, 127.12 (alkene-CH), 125.81, 125.42, 124.80 (alkene-CH), 67.89 (CH_2_–O), 60.03 (CH_2_–O), 53.00 (CH_2_–N), 52.54 (CH_2_–N) ppm. ^19^F NMR (470 MHz, DMSO-d_6_) *δ* −164.65 (2F, F3,5-py).

#### 3,5-Difluoro-2,4,6-tris((1-(4-(naphthalen-2-yl)benzyl)-1*H*-1,2,3-triazol-4-yl)methoxy)pyridine 10b

White solid. Yield 70%. MP 263–265 °C; IR (KBr) *ν̃* 3424, 3052, 2952, 1626, 1593, 1491 cm^−1^. ^1^H-NMR (500 MHz, DMSO-d_6_) *δ* 8.37 (s, 3H, tetrazole-H), 8.29–8.30 (d, *J* = 6 Hz, 2H), 8.00–8.05 (m, 9H), 7.95 (d, *J* = 7.8 Hz, 2H), 7.52–7.66 (m, 12H, Ar–H), 7.25 (d, *J* = 8.1 Hz, 4H), 7.20 (d, *J* = 8.7 Hz, 2H), 5.57–5.58 (s, 6, CH_2_O), 5.45 (s, 4H, CH_2_–N), 5.40 (s, 2H, CH_2_–N) ppm. ^13^C NMR (126 MHz, DMSO-d_6_) *δ* 143.46 (m), 143.03, 142.40 (m), 136.74 (d, ^1^*J*_CF_ = 245.0 Hz, C3,5-py), 135.63, 133.86, 132.77 (triazole-C), 132.12 (triazole-C), 132.10, 130.65 (Ar–C), 130.49, 128.98 (Ar–CH), 128.67 (Ar–CH), 127.95 (Ar–CH), 126.90, 126.63, 126.03 (alkene-CH), 125.82, 125.70, 125.42 (alkene-CH), 121.86, 66.62 (CH_2_–O), 60.01 (CH_2_–O), 52.57 (CH_2_–N), 52.54 (CH_2_–N) ppm. ^19^F NMR (470 MHz, DMSO-d_6_) *δ* −164.65 (2F, F3,5-py).

#### 4′-((4-(((2,6-Bis((1-(4-bromobenzyl)-1*H*-1,2,3-triazol-4-yl)methoxy)-3,5-difluoropyridin-4-yl)oxy)methyl)-1*H*-1,2,3-triazol-1-yl)methyl)-[1,1′-biphenyl]-4-carbaldehyde 10c

White solid. Yield 62%. MP 285–288 °C; IR (KBr) *ν̃* 3725, 3424, 3136, 3061, 2958, 28.60, 1724. ^1^H-NMR (500 MHz, DMSO-d_6_) *δ* 10.07 (s, 1H, CHO), 8.21–8.34 (s, 3H, tetrazole-H), 8.03 (d, 2H, *J* = 8.4 Hz, Ar), 7.99 (d, 2H, *J* = 8.2 Hz), 7.84–7.87 (d, *J* = 8.2 Hz, 2H), 7.52–7.55 (m, 6H, Ar–H), 7.25–7.28 (m, 4H), 7.20 (d, 2H, *J* = 8.4 Hz), 5.58 (s, 6, CH_2_O), 5.45 (s, 4H, CH_2_–N), 5.40 (s, 2H, CH_2_–N). ^13^C-NMR (126 MHz, DMSO-d_6_) *δ* 193.14, 144.88 (dm, ^2^*J*_CF_ = 10.01 Hz, C2,6-py), 144.06 (m), 143.96, 135.74, 134 (d, ^1^*J*_CF_ = 242.5 Hz, C3,5-py), 132.18, 132.10, 131.96 (triazole-C), 130.65 (triazole-C), 130.60 (Ar–C), 130.52, 129.10 (Ar–CH), 128.33 (Ar–CH), 127.96 (Ar-CH), 127.80, 127.57, 125.43 (alkene-CH), 121.90, 115.67 (alkene-CH), 67.90 (CH_2_–O), 60.01 (CH_2_–O), 58.91 (CH_2_–N), 52.59 (CH_2_–N) ppm. ^19^F NMR (470 MHz, DMSO-d_6_) *δ* −164.61 (2F, F3,5-py).

#### 2,4,6-Tris((1-([1,1′-biphenyl]-4-ylmethyl)-1*H*-1,2,3-triazol-4-yl)methoxy)-3,5-dichloropyridine 10d

White solid. Yield 73%. MP 232–234 °C; IR (KBr) *ν̃* 3060, 2952, 1578, 1561, 1489 cm^−1^. ^1^H-NMR (500 MHz, DMSO-d_6_) *δ* 8.24 (s, 3H, tetrazole-H), 7.60–7.66 (m, 5H, Ar), 7.53–7.56 (m, 5H, Ar), 7.42–7.47 (m, 5H, Ar–H), 7.334–7.40 (m, 6H, Ar), 7.24–7.29 (m, 4H, Ar), 7.20–7.23 (d, 2H, *J* = 8.2 Hz), 5.55–5.63 (m, 6, CH_2_O), 5.45–5.54 (m, 4H, CH_2_–N), 5.37–5.44 (m, 2H, CH_2_–N) ppm. ^13^C NMR (126 MHz, DMSO-d_6_) *δ* 146.05, 142.95, 135.88, 135.77, 134.94, 132.13, 132.08, 130.68 (triazole-C), 130.63 (triazole-C), 130.47 (Ar–C), 129.35, 129.06 (Ar–CH), 128.81 (Ar–CH), 128.02 (Ar–CH), 127.84 (Ar–CH), 127.50 (Ar–CH), 127.12 (Ar–CH), 126.26, 125.99, 125.42 (alkene-CH), 125.12, 121.90, 121.83 (alkene-CH), 66.59 (CH_2_–O), 60.79 (CH_2_–O), 52.58 (CH_2_–N), 52.49 (CH_2_–N) ppm.

#### 3,5-Dichloro-2,4,6-tris((1-(4-(naphthalen-2-yl)benzyl)-1*H*-1,2,3-triazol-4-yl)methoxy)pyridine 10e

White solid. Yield 69%. MP 274–276 °C; IR (KBr) *ν̃* 3449, 3052, 1580, 1490, 1412 cm^−1^. ^1^H-NMR (500 MHz, DMSO-d_6_) *δ* 8.37 (s, 3H, tetrazole-H), 8.29 (d, *J* = 6 Hz, 3H), 8.00–8.05 (m, 8H), 7.95 (d, *J* = 7.8 Hz, 4H), 7.53–7.56 (m, 12H, Ar–H), 7.26 (d, *J* = 8.1 Hz, 4H), 7.20 (d, *J* = 8.7 Hz,2H), 5.59 (s, 6, CH_2_O), 5.51 (s, 4H, CH_2_–N), 5.22 (s, 2H, CH_2_–N) ppm. ^13^C NMR (126 MHz, DMSO-d_6_) *δ* 143.91, 143.21, 141.99, 137.72, 135.76, 137.73, 133.86,132.77, 132.74, 132.13 (triazole-C), 132.08 (triazole-C), 130.68 (Ar–C),128.99, 128.67, 127.96 (Ar–CH), 126.91 (Ar–CH), 126.64 (Ar–CH), 126.30, 126.04, 125.71 (alkene-CH), 125.44, 121.90 (alkene-CH), 66.97 (CH_2_–O), 60.74 (CH_2_–O), 52.59 (CH_2_–N), 52.44 (CH_2_–N) ppm.

#### 4′-((4-(((2,6-Bis((1-(4-bromobenzyl)-1*H*-1,2,3-triazol-4-yl)methoxy)-3,5-dichloropyridin-4-yl)oxy)methyl)-1*H*-1,2,3-triazol-1-yl)methyl)-[1,1′-biphenyl]-4-carbaldehyde 10f

White solid. Yield 56%. MP 280 °C (dec); IR (KBr) *ν̃* 3726, 3448, 3138, 2951, 2841, 1694. ^1^H NMR (500 MHz, DMSO-d_6_). *δ* 10.07 (s, 3H, CHO), 8.30 (s, 3H, tetrazole-H), 8.03 (d, 2H, *J* = 8.4 Hz), 7.99 (d, 2H, *J* = 8.2 Hz), 7.49–7.57 (m, 6H), 7.24–7.31 (m, 4H), 7.21 (d, 2H, *J* = 8.5 Hz), 5.56–5.62 (m, 6, CH_2_O), 5.480–5.54 (m, 4H, CH_2_–N), 5.24 (s, 2H, CH_2_–N) ppm. ^13^C NMR (126 MHz, DMSO-d_6_): *δ* 193.19, 155.74, 144.83, 142.93, 142.35, 136.25, 135.78, 132.13, 132.08, 130.69 (triazole-C), 130.62 (Ar–C), 130.47, 128.35 (Ar-CH), 127.81 (Ar–CH), 125.47, 121.92, 103.69, 67.56 (CH_2_–O), 60.76 (CH_2_–O), 52.58 (CH_2_–N), 52.51 (CH_2_–N) ppm.

## 4. Conclusions

In summary, perhalopyridine-based alkyne precursors 3a and 3b were prepared through the S_N_Ar reaction of PFP and PCP with excess amounts of propargyl alcohol. We have accordingly developed the click reaction of the derived alkynes with aryl azides 4a–c under ultrasonic irradiation as an effective method for synthesizing poly(1,2,3-triazolyl)-substituted perhalopyridines 5a–f. However, aliphatic 1,2,3-triazole analogues 7a–d were formed *via* the sonication reaction of pyridyl cores 3, alkyl bromides 6a,b, and NaN_3_ under one-pot conditions. Utilizing 2,3,4,5-tetrachloro-6-(prop-2-yn-1-yloxy)pyridine 3c, drived from the regioselective S_N_Ar reaction of PCP with propargyl alcohol, we also succeeded to furnish several ((1,2,3-triazol-4-yl)methoxy)-3,4,5,6-tetrachloropyridines 8a–c under the reaction conditions. Finally, biaryl-embedded perfluoropyridine-based poly(1,2,3-triazoles) 10a–f were produced in good yields *via* the SMC reaction of tris-triazole 5b,e with arylboronic acids 9a–c under Pd(PPh_3_)_4_ catalysis.

## Author contributions

Fereshteh Khorasani: conceptualization, investigation, methodology, data analysis, writing – review & editing; Reza Ranjbar-Karimi and Kazem Mohammadiannejad: investigation, writing – review & editing; Reza Ranjbar-Karimi: supervision.

## Conflicts of interest

There are no conflicts to declare.

## Supplementary Material

RA-014-D4RA05861E-s001

RA-014-D4RA05861E-s002
