# Supplementary material for: Utilizing perhalopyridine-based alkynes as suitable precursors for the synthesis of novel poly(1,2,3-triazolyl)-substituted perhalopyridines
Source: RSC Adv. 2024 Sep 27;14(42):30873–85. doi: 10.1039/d4ra05861e (PMC11427873; doi:10.1039/d4ra05861e)
Supplement: RA-014-D4RA05861E-s001 [file RA-014-D4RA05861E-s001.pdf]

Supplementary Information (SI) for RSC Advances.
This journal is © The Royal Society of Chemistry 2024

Electronic Supplementary Information

For

Utilizing perhalopyridine-based alkynes as suitable precursors for the synthesis of novel poly(1,2,3-triazolyl)-substituted perhalopyridines

Fereshteh Khorasani[a], Reza Ranjbar-Karimi*[a] and Kazem Mohammadiannejad [b]

[a]Department of Chemistry, Vali-e-Asr University of Rafsanjan, Rafsanjan 77176, Islamic Republic of Iran.

[b]NMR Laboratory, Faculty of Science, Vali-e-Asr University of Rafsanjan, Rafsanjan 77176, Islamic Republic of Iran.

*Corresponding author, Fax: +98-343-131-2429; Tel: +98-343-131-2429, E-mail: r.ranjbarkarimi@vru.ac.ir (R. Rnjbar-Karimi).

1. Crystal structure of **3a.**

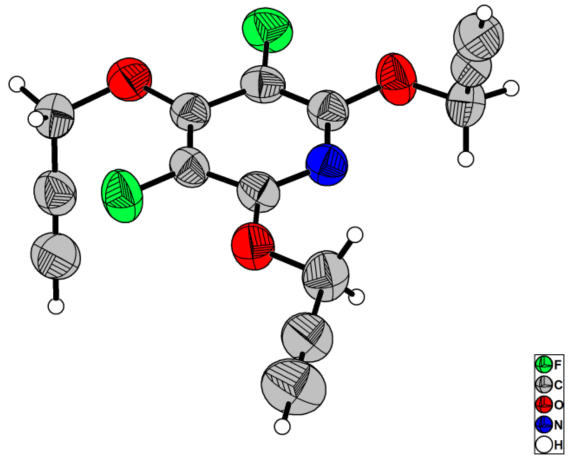

2. Crystal structure of **8b.**

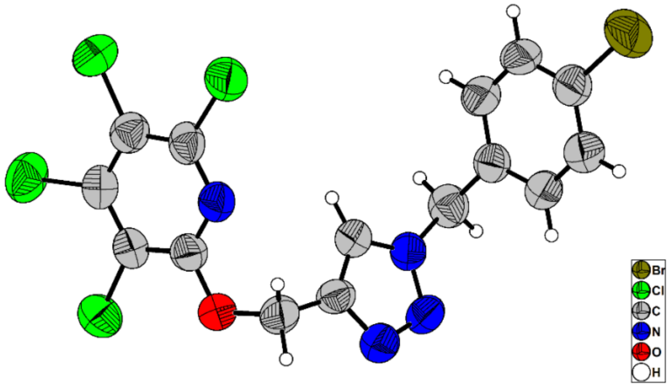

3. NMR spectra *of the synthesized compounds **3a-c***

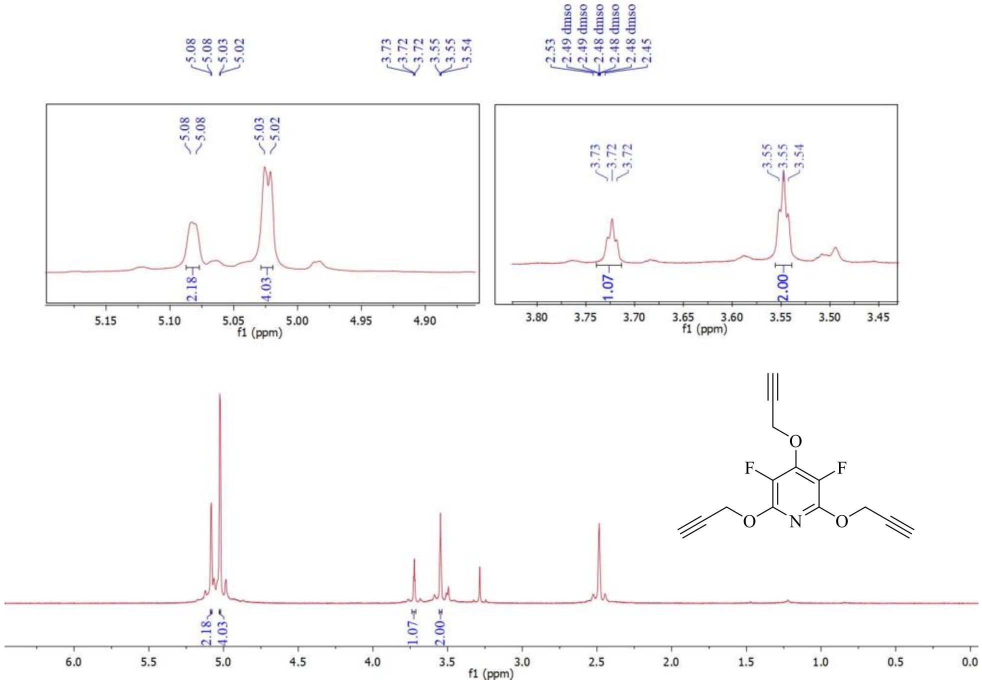

^{1}H NMR spectrum of 3,5-difluoro-2,4,6-tris(prop-2-yn-1-yloxy)pyridine **3a**

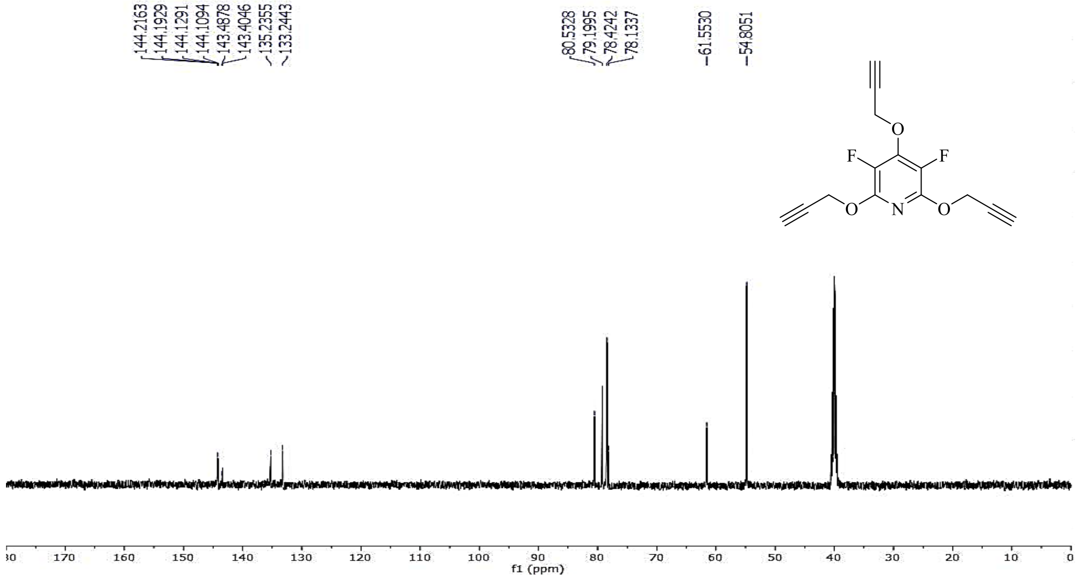

^{13}C NMR spectrum of 3,5-difluoro-2,4,6-tris(prop-2-yn-1-yloxy)pyridine **3a**

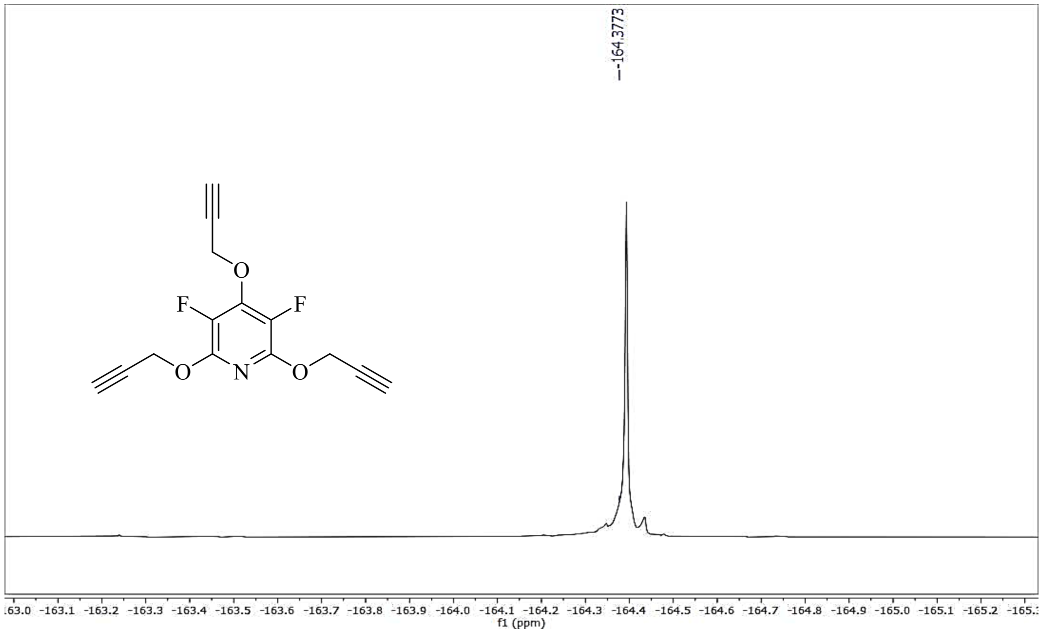

^{19}F NMR spectrum of 3,5-difluoro-2,4,6-tris(prop-2-yn-1-yloxy)pyridine **3a**

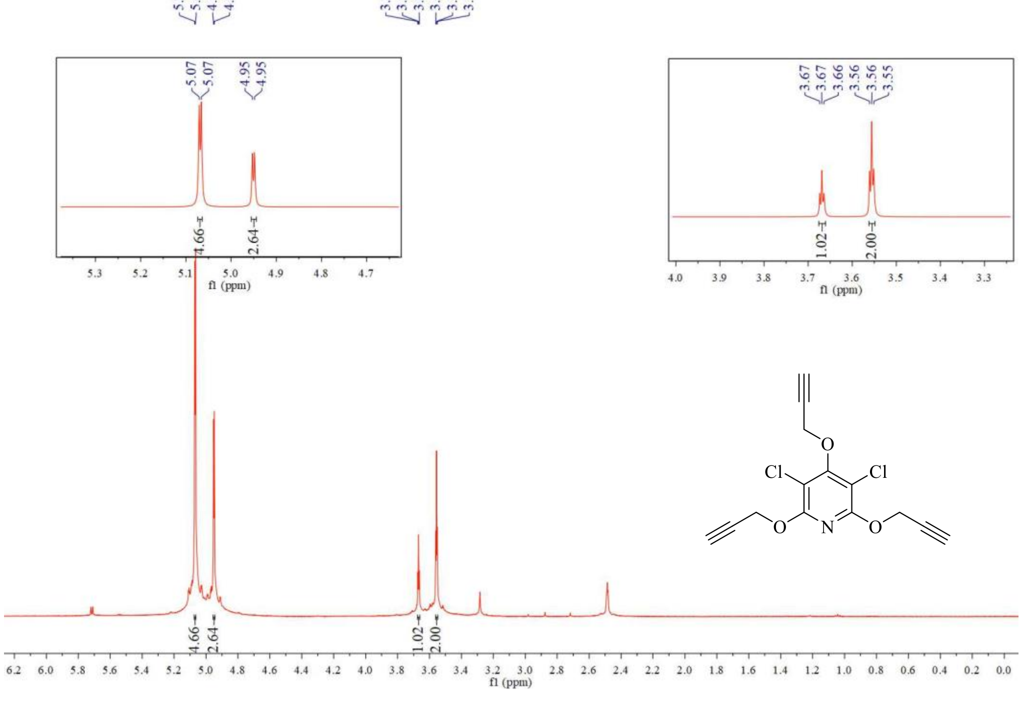

^{1}H NMR spectrum of 3,5-dichloro-2,4,6-tris(prop-2-yn-1-yloxy)pyridine **3b**

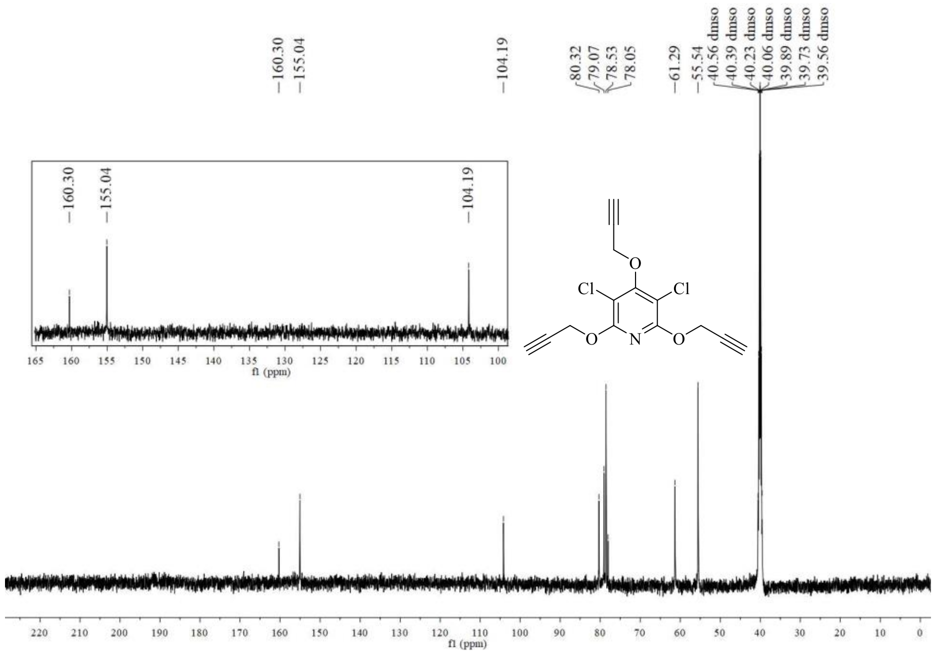

^{13}C NMR spectrum of 3,5-dichloro-2,4,6-tris(prop-2-yn-1-yloxy)pyridine **3b**

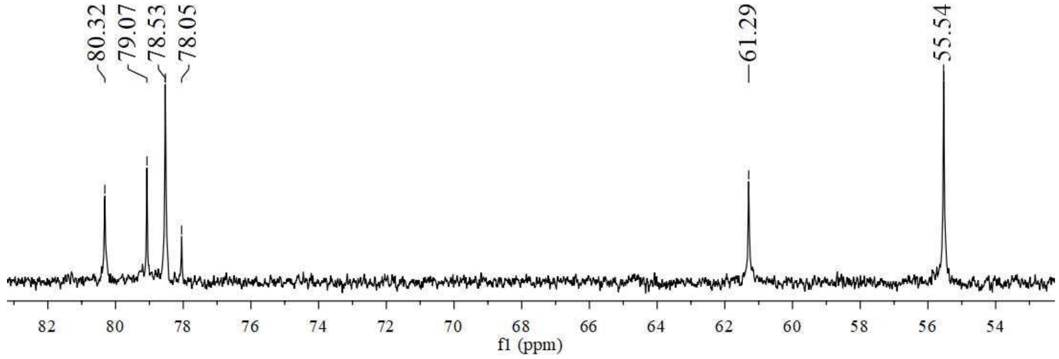

Expanded ^{13}C NMR spectrum of 3,5-dichloro-2,4,6-tris(prop-2-yn-1-yloxy)pyridine **3b**

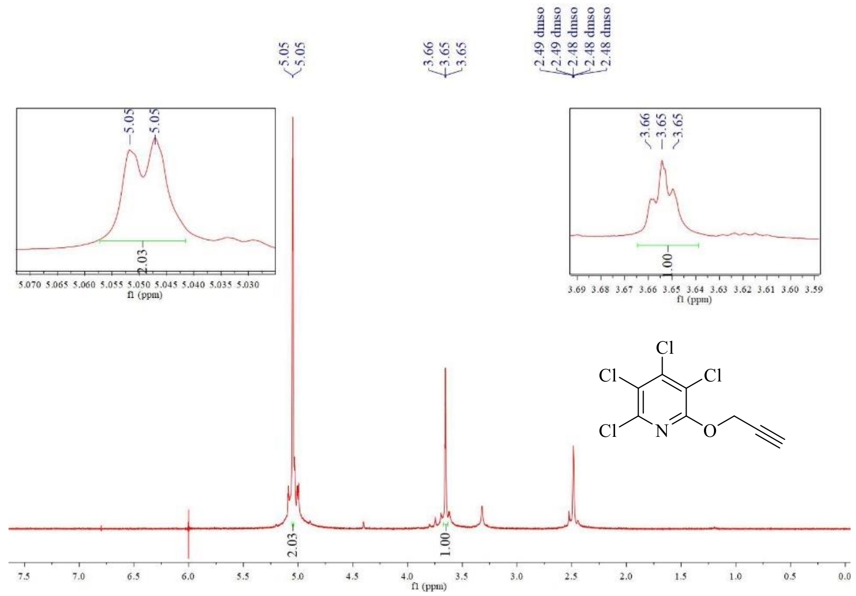

^{1}H NMR spectrum of 2,3,4,5-tetrachloro-6-(prop-2-yn-1-yloxy)pyridine **3c**

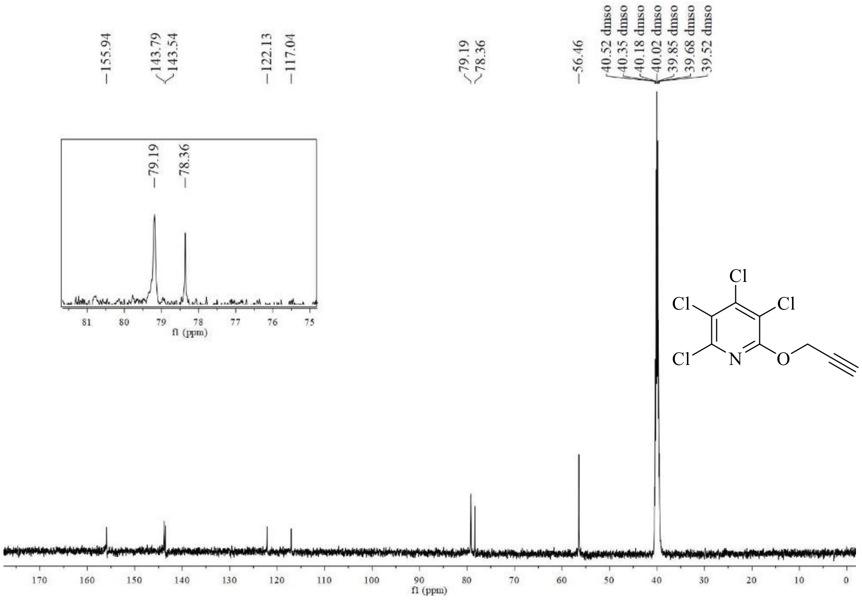

^{13}C NMR spectrum of 2,3,4,5-tetrachloro-6-(prop-2-yn-1-yloxy)pyridine **3c**

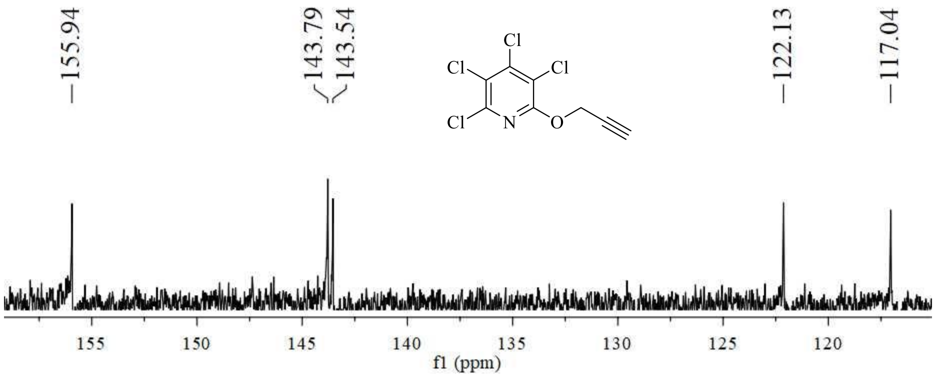

Expanded ^{13}C NMR spectrum of 2,3,4,5-tetrachloro-6-(prop-2-yn-1-yloxy)pyridine **3c**

6. NMR spectra of synthesized tris(1,2,3-triazoles) 5a-f and 7a-d

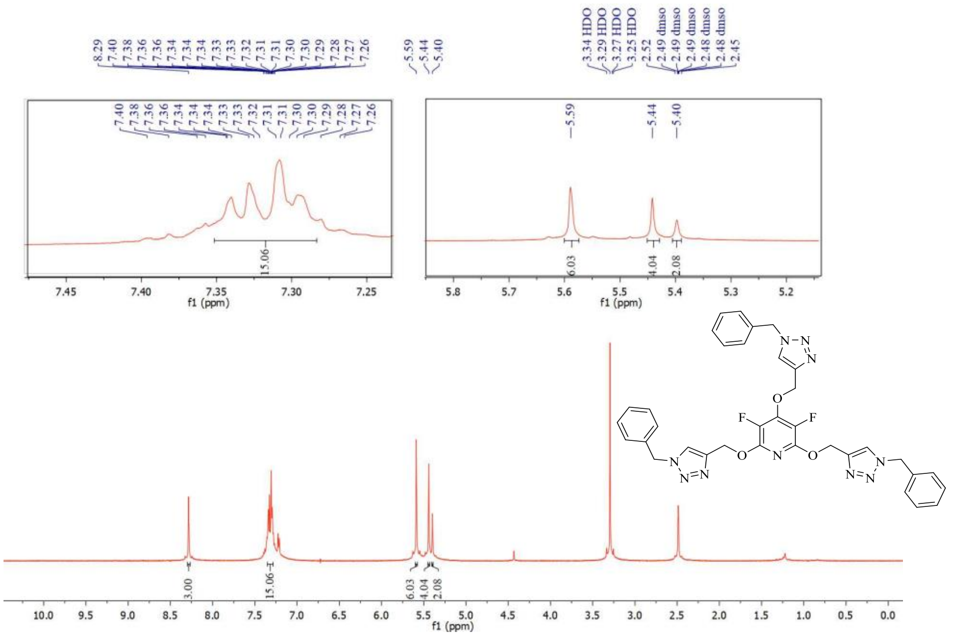

^{1}H NMR spectrum of 2,4,6-tris((1-benzyl-1H-1,2,3-triazol-4-yl)methoxy)-3,5-difluoropyridine **5a**

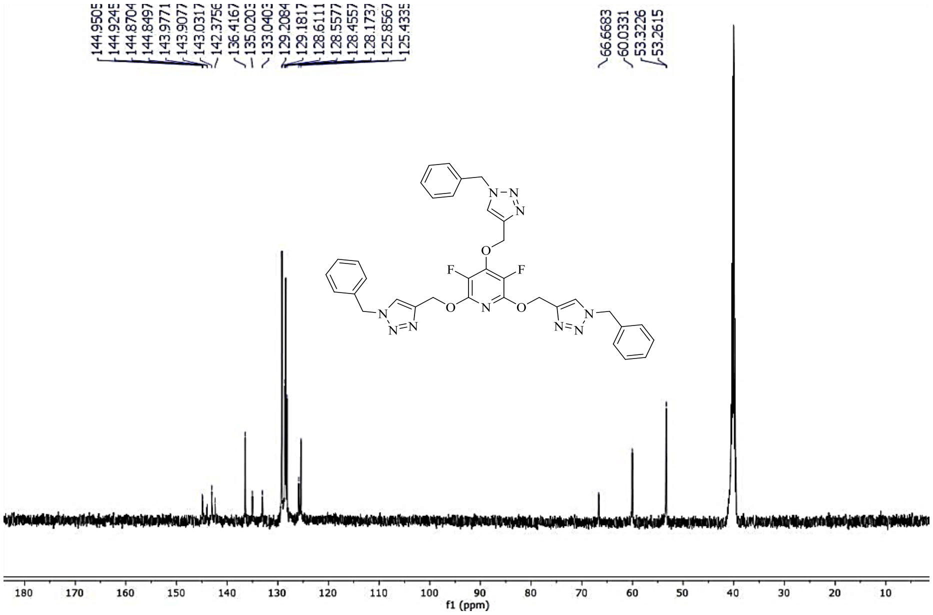

^{13}C NMR spectrum of 2,4,6-tris((1-benzyl-1H-1,2,3-triazol-4-yl)methoxy)-3,5-difluoropyridine **5a**

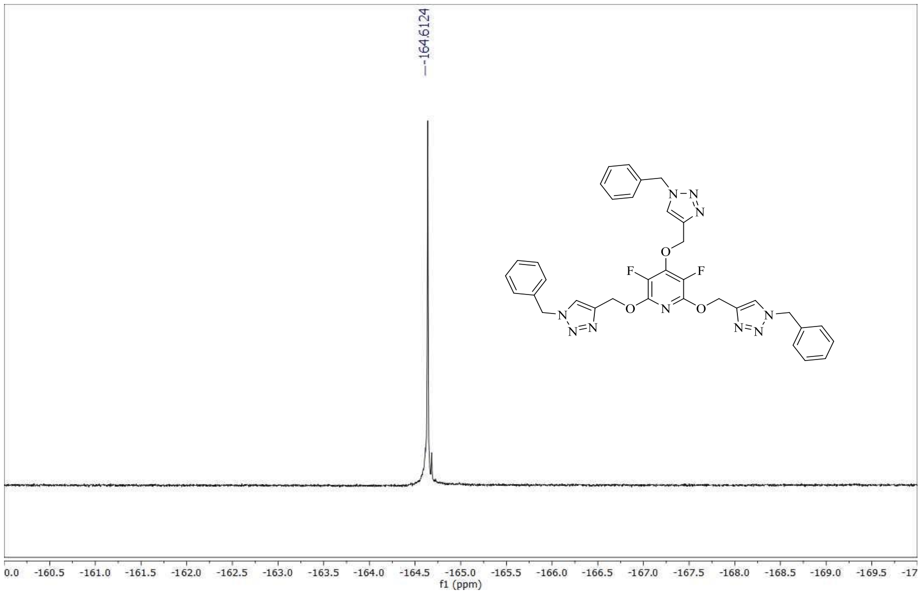

^{19}F NMR spectrum of 2,4,6-tris((1-benzyl-1H-1,2,3-triazol-4-yl)methoxy)-3,5-difluoropyridine **5a**

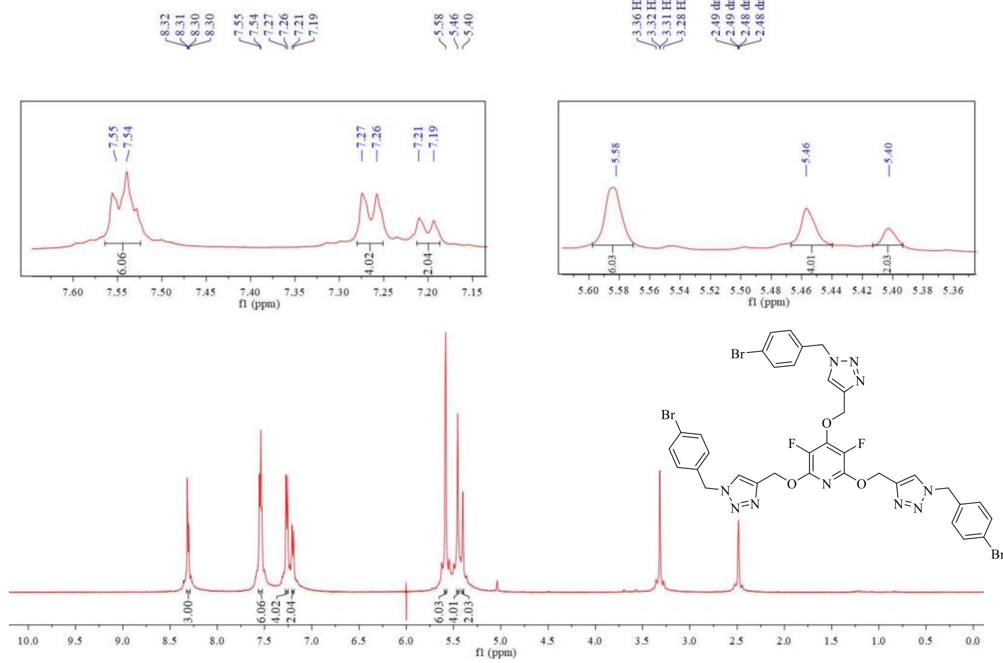

^{1}H NMR spectrum of 2,4,6-tris((1-(4-bromobenzyl)-1H-1,2,3-triazol-4-yl)methoxy)-3,5-difluoropyridine **5b**

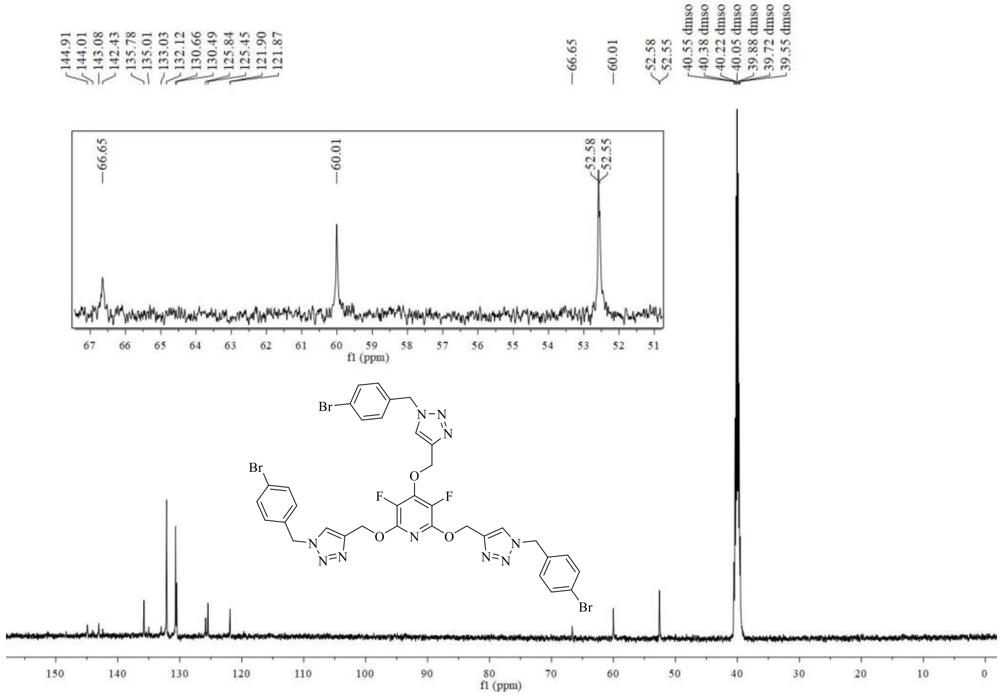

^{13}C NMR spectrum of 2,4,6-tris((1-(4-bromobenzyl)-1H-1,2,3-triazol-4-yl)methoxy)-3,5-difluoropyridine **5b**

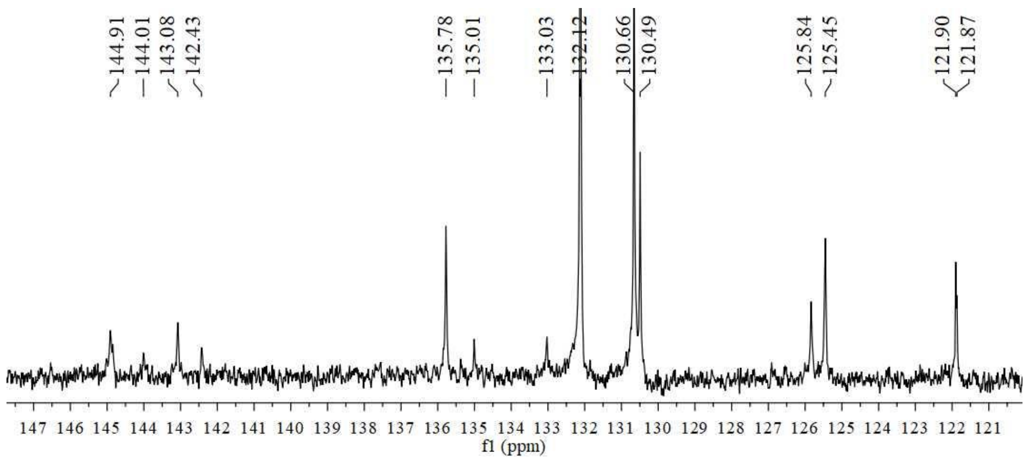

Expanded ^{13}C NMR spectrum of 2,4,6-tris((1-(4-bromobenzyl)-1H-1,2,3-triazol-4-yl)methoxy)-3,5-difluoropyridine **5b**

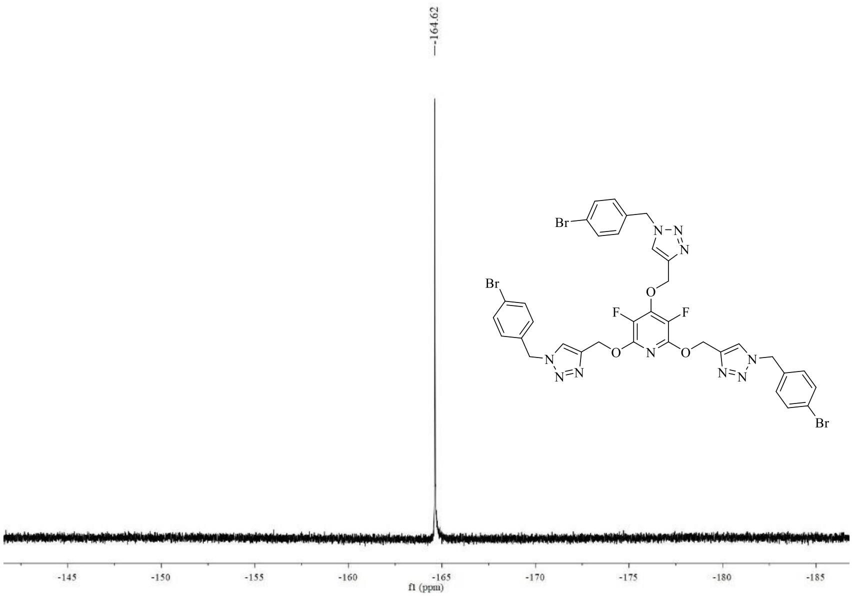

^{19}F NMR spectrum of 2,4,6-tris((1-(4-bromobenzyl)-1H-1,2,3-triazol-4-yl)methoxy)-3,5-difluoropyridine **5b**

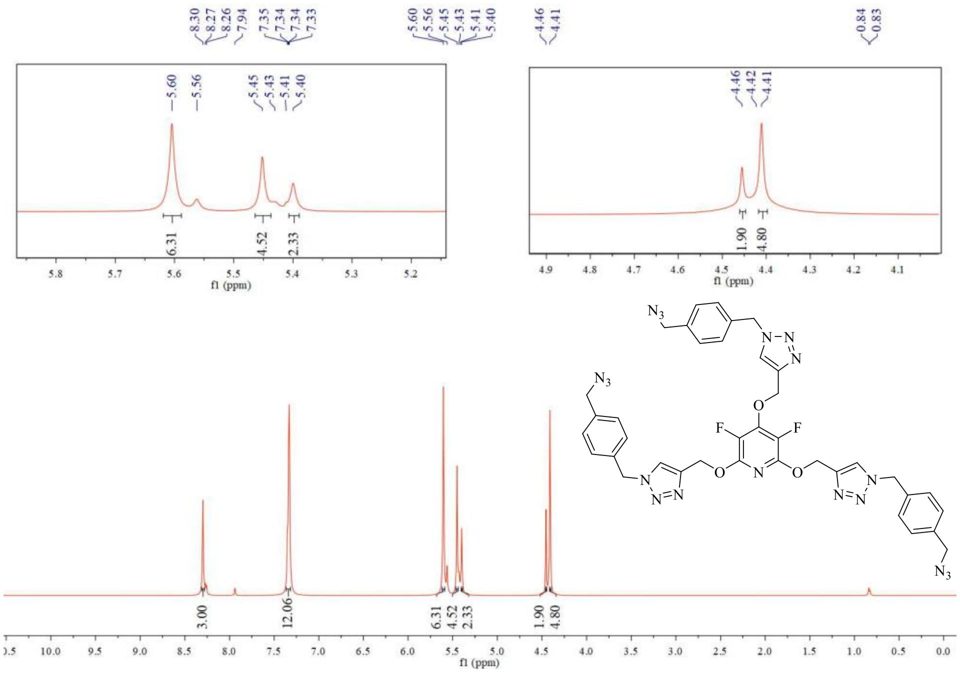

^{1}H NMR spectrum of 2,4,6-tris((1-(4-(azidomethyl)benzyl)-1H-1,2,3-triazol-4-yl)methoxy)-3,5-difluoropyridine **5c**

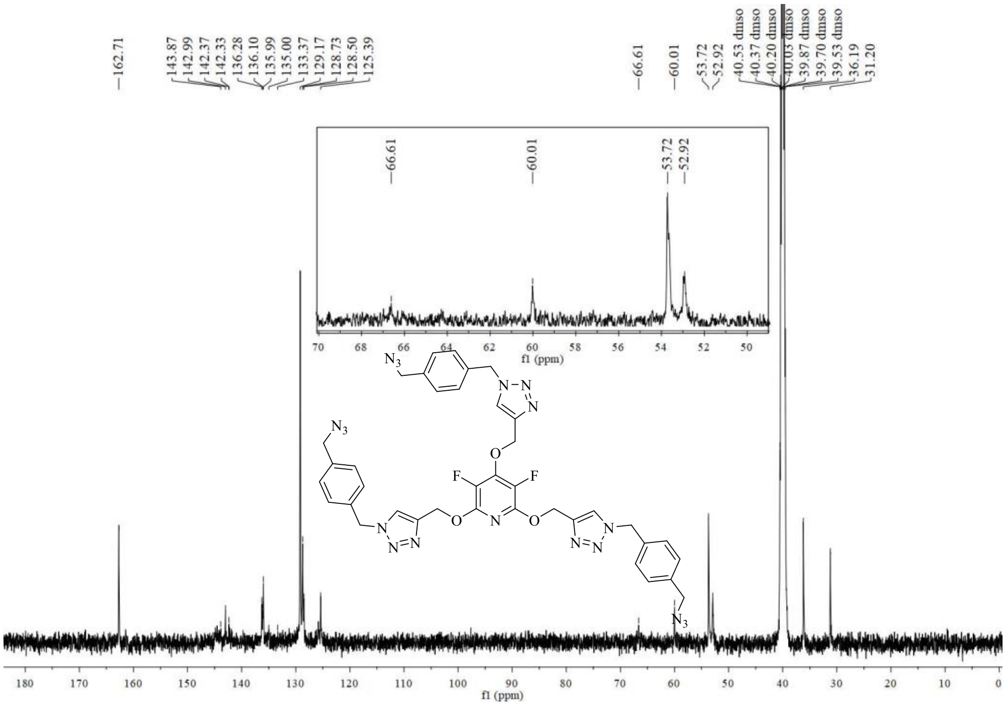

^{13}C NMR spectrum of 2,4,6-tris((1-(4-(azidomethyl)benzyl)-1H-1,2,3-triazol-4-yl)methoxy)-3,5-difluoropyridine **5c**

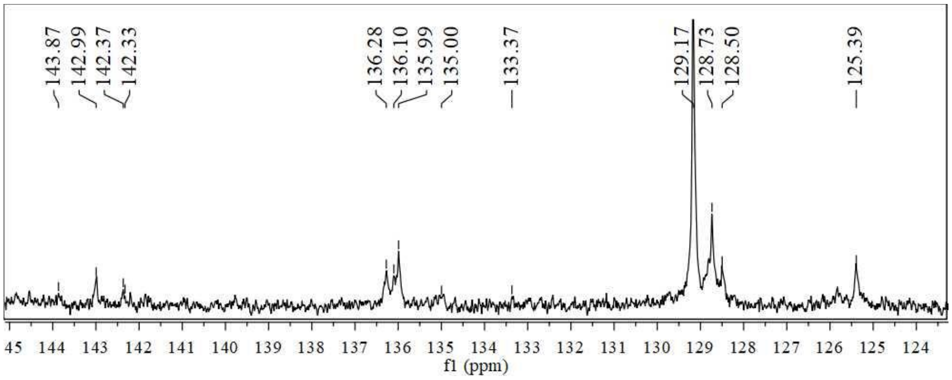

Expanded ^{13}C NMR spectrum of 2,4,6-tris((1-(4-(azidomethyl)benzyl)-1H-1,2,3-triazol-4-yl)methoxy)-3,5-difluoropyridine **5c**

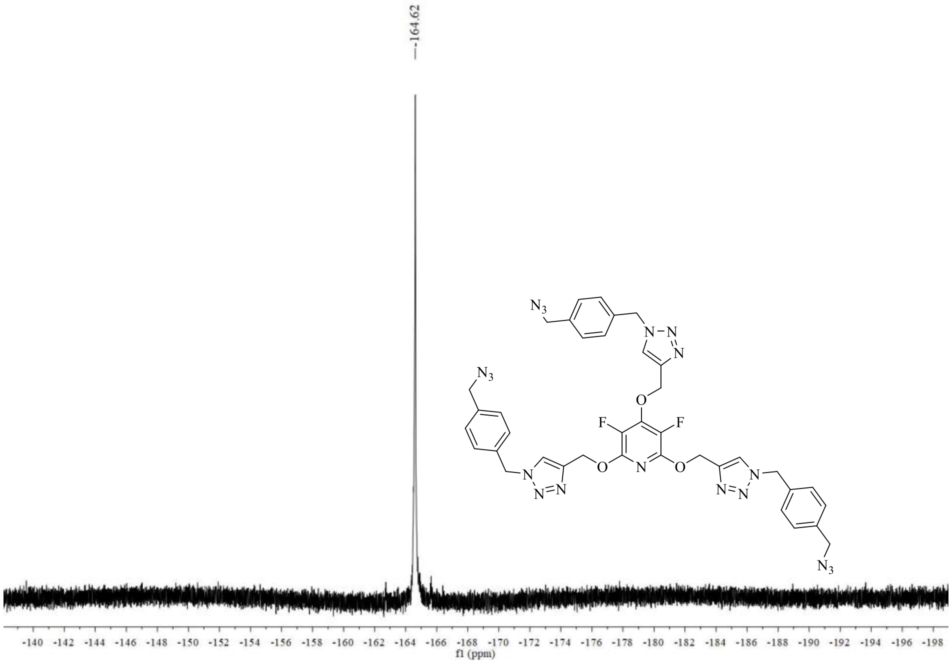

^{19}F NMR spectrum of 2,4,6-tris((1-(4-(azidomethyl)benzyl)-1H-1,2,3-triazol-4-yl)methoxy)-3,5-difluoropyridine **5c**

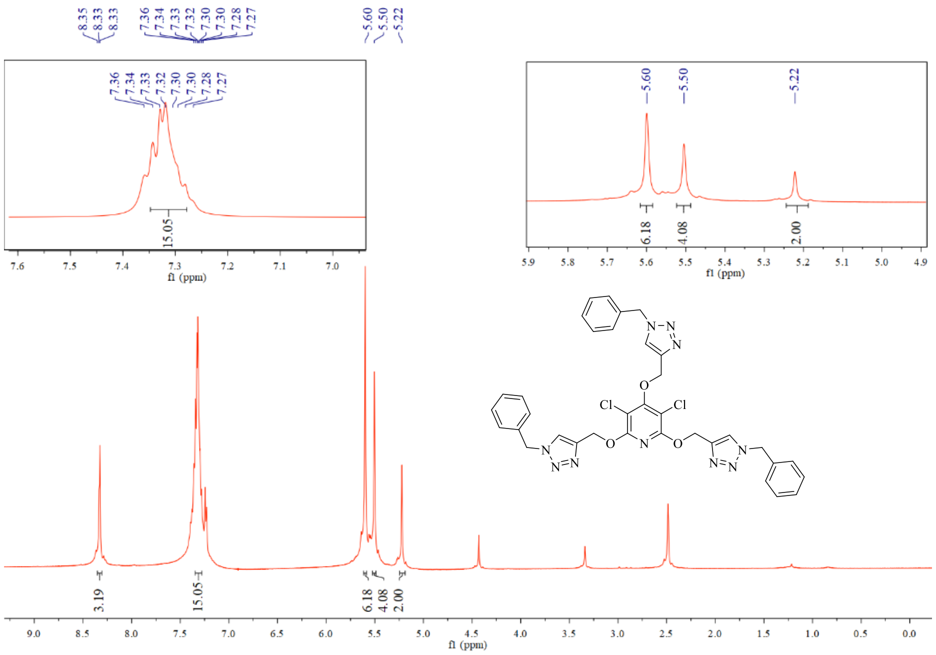

^{1}H NMR spectrum of 2,4,6-tris((1-benzyl-1H-1,2,3-triazol-4-yl)methoxy)-3,5-dichloropyridine **5d**

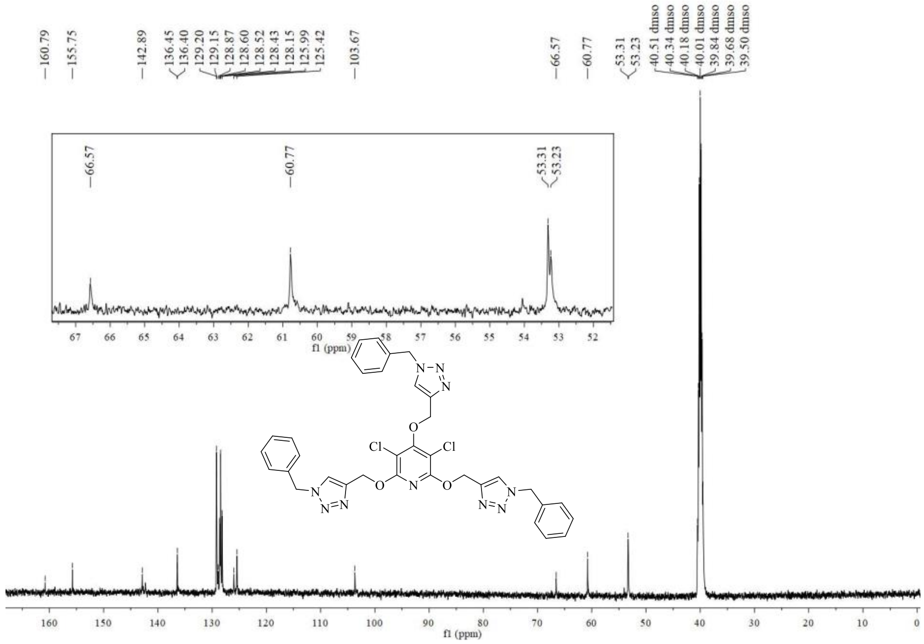

^{13}C NMR spectrum of 2,4,6-tris((1-benzyl-1H-1,2,3-triazol-4-yl)methoxy)-3,5-dichloropyridine **5d**

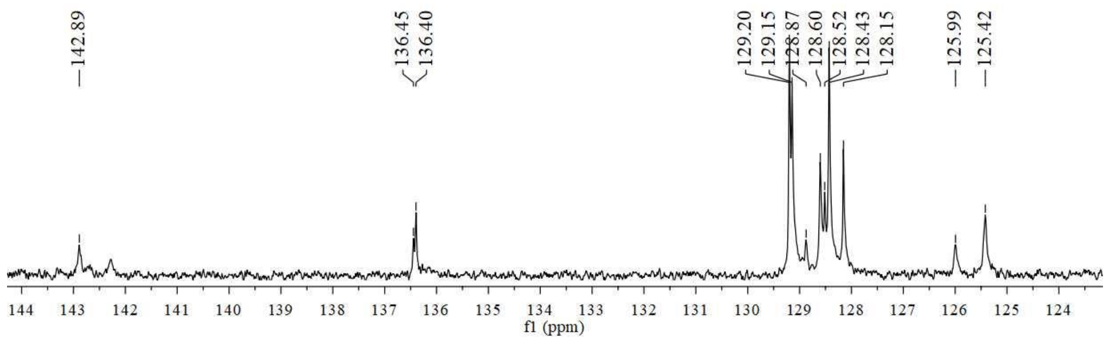

Expanded ^{13}C NMR spectrum of 2,4,6-tris((1-benzyl-1H-1,2,3-triazol-4-yl)methoxy)-3,5-dichloropyridine **5d**

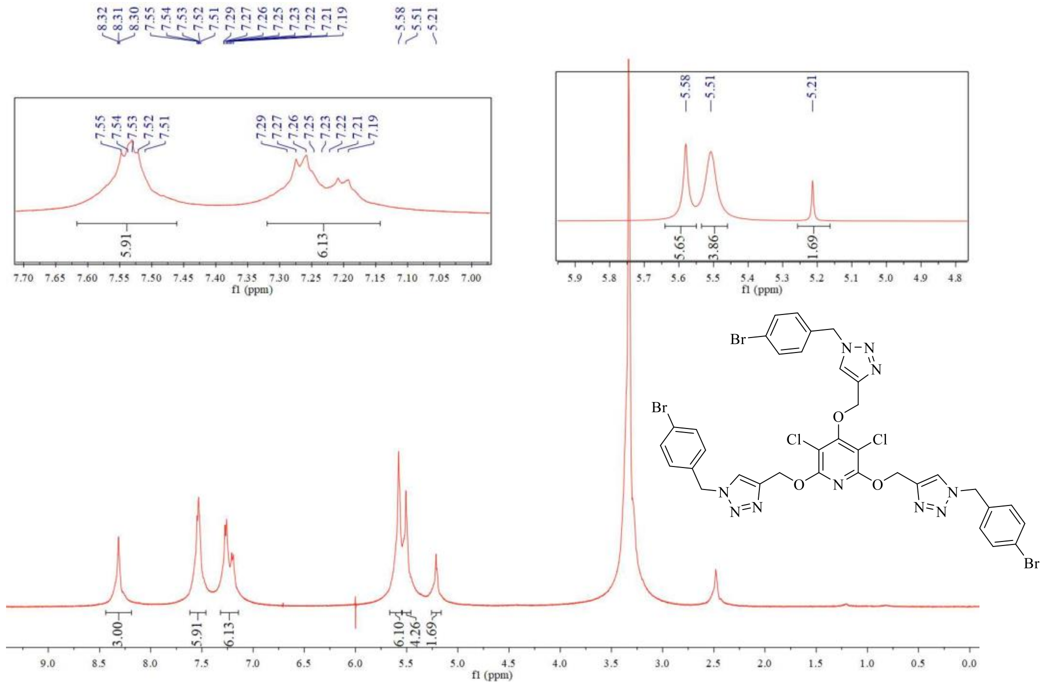

^{1}H NMR spectrum of 2,4,6-tris((1-(4-bromobenzyl)-1H-1,2,3-triazol-4-yl)methoxy)-3,5-dichloropyridine **5e**

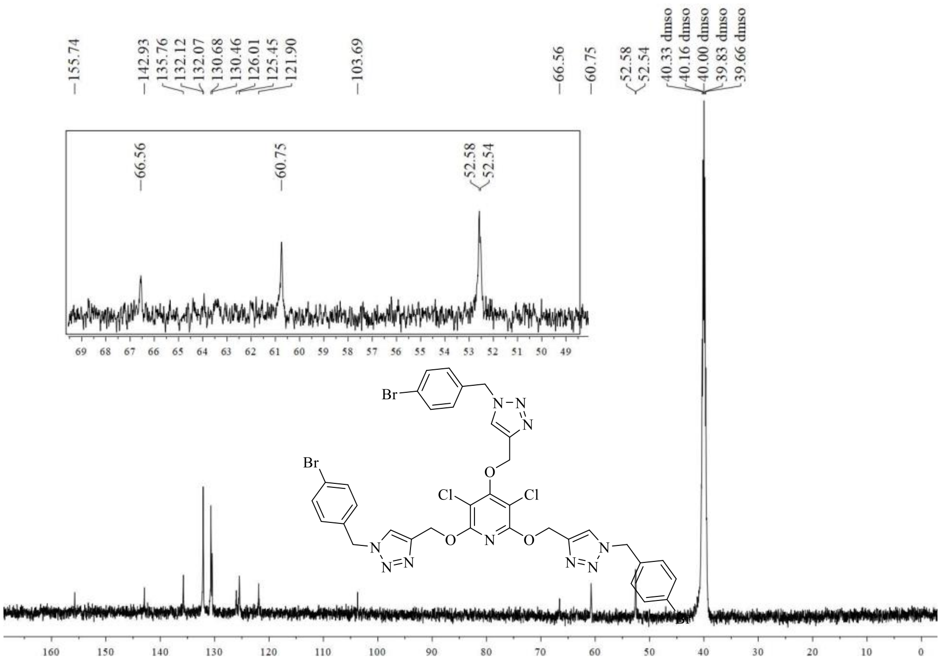

^{13}C NMR spectrum of 2,4,6-tris((1-(4-bromobenzyl)-1H-1,2,3-triazol-4-yl)methoxy)-3,5-dichloropyridine **5e**

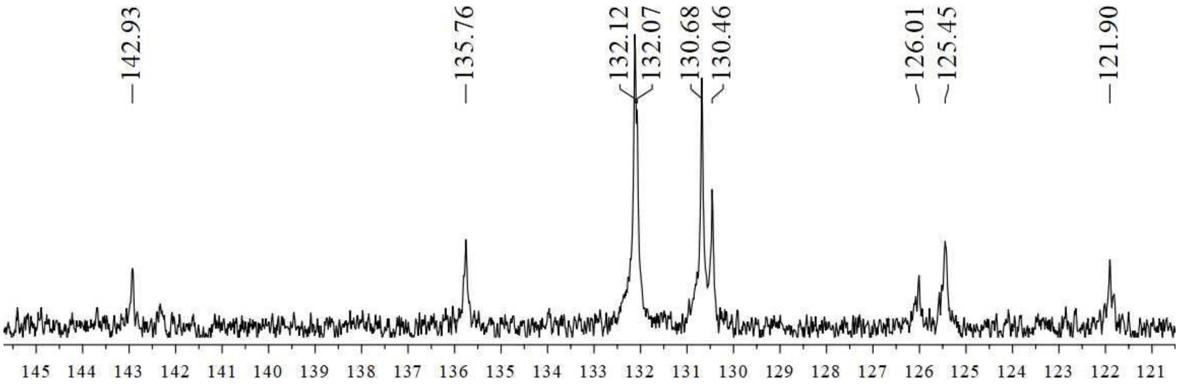

Expanded ^{13}C NMR spectrum of 2,4,6-tris((1-(4-bromobenzyl)-1H-1,2,3-triazol-4-yl)methoxy)-3,5-dichloropyridine **5e**

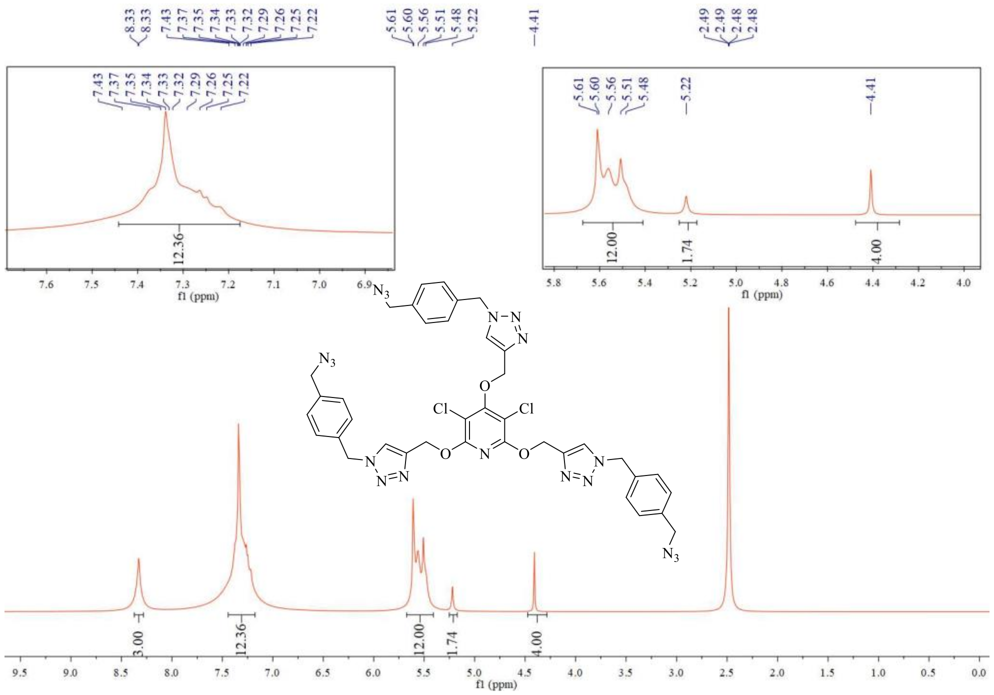

^{1}H NMR spectrum of 2,4,6-tris((1-(4-(azidomethyl)benzyl)-1H-1,2,3-triazol-4-yl)methoxy)-3,5-dichloropyridine **5f**

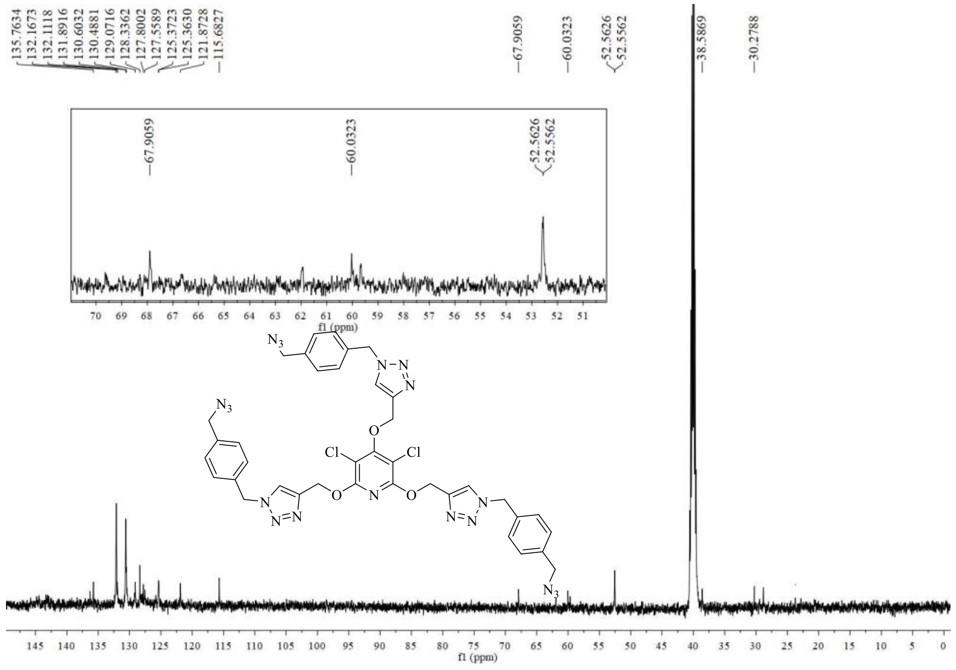

^{13}C NMR spectrum of 2,4,6-tris((1-(4-(azidomethyl)benzyl)-1H-1,2,3-triazol-4-yl)methoxy)-3,5-dichloropyridine **5f**

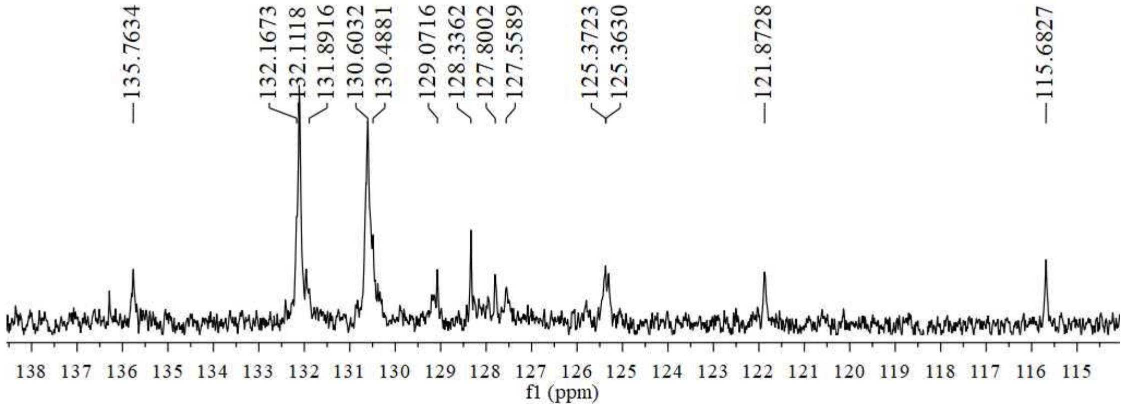

Expanded ^{13}C NMR spectrum of 2,4,6-tris((1-(4-(azidomethyl)benzyl)-1H-1,2,3-triazol-4-yl)methoxy)-3,5-dichloropyridine **5f**

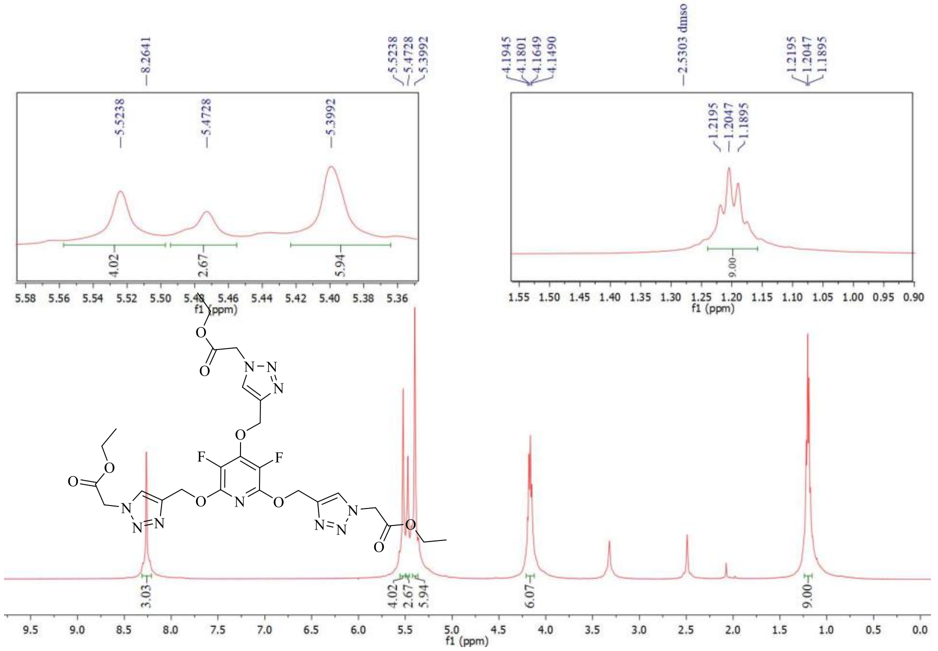

^{1}H NMR spectrum of triethyl 2,2',2''-((((3,5-difluoropyridine-2,4,6-triyl)tris(oxy))tris(methylene))tris(1H-1,2,3-triazole-4,1-diyl))triacetate **7a**

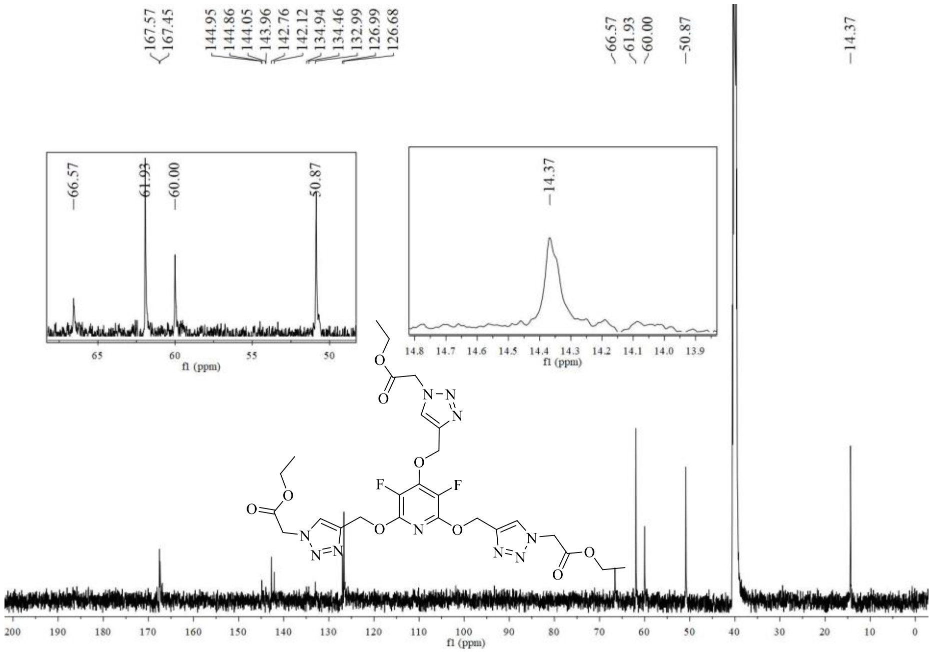

^{13}C NMR spectrum of triethyl 2,2',2''-((((3,5-difluoropyridine-2,4,6-triyl)tris(oxy))tris(methylene))tris(1H-1,2,3-triazole-4,1-diyl))triacetate **7a**

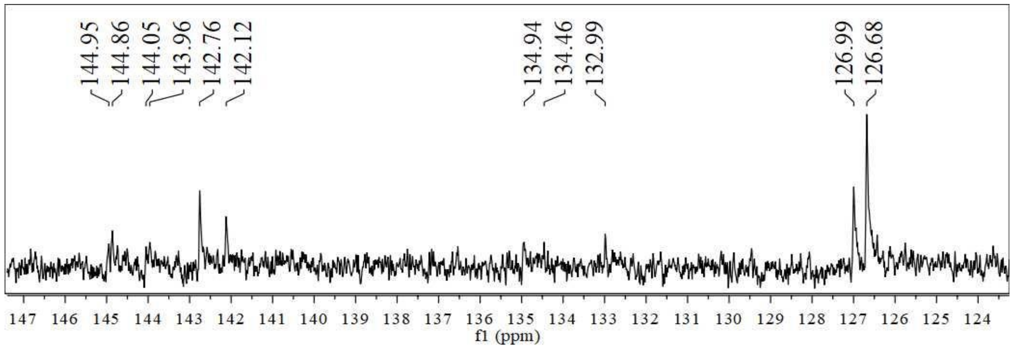

Expanded ^{13}C NMR spectrum of triethyl 2,2',2''-((((3,5-difluoropyridine-2,4,6-triyl)tris(oxy))tris(methylene))tris(1H-1,2,3-triazole-4,1-diyl))triacetate **7a**

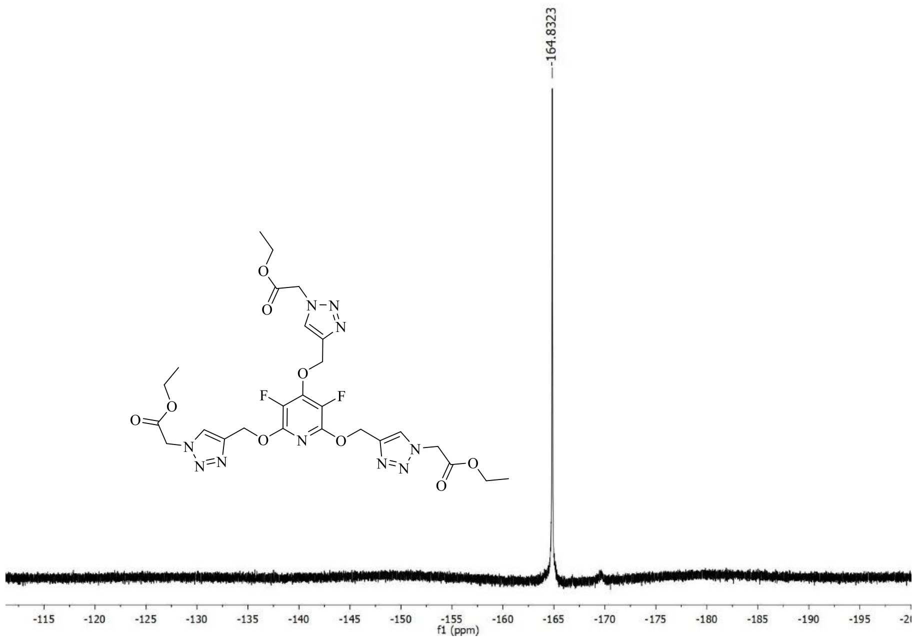

^{19}F NMR spectrum of triethyl 2,2',2''-((((3,5-difluoropyridine-2,4,6-triyl)tris(oxy))tris(methylene))tris(1H-1,2,3-triazole-4,1-diyl))triacetate **7a**

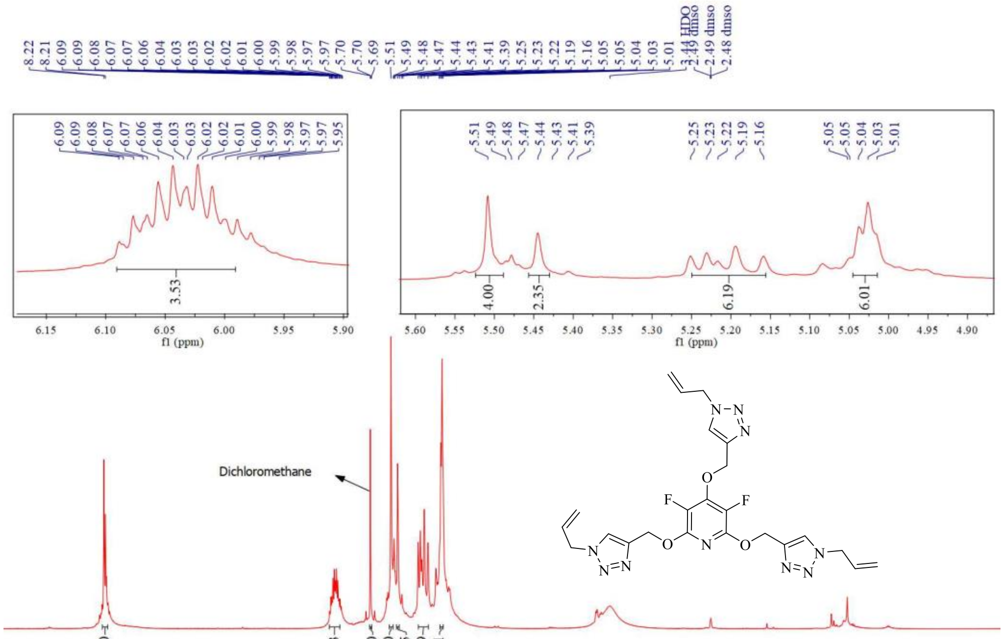

^{1}H NMR spectrum of 2,4,6-tris((1-allyl-1H-1,2,3-triazol-4-yl)methoxy)-3,5-difluoropyridine **7b**

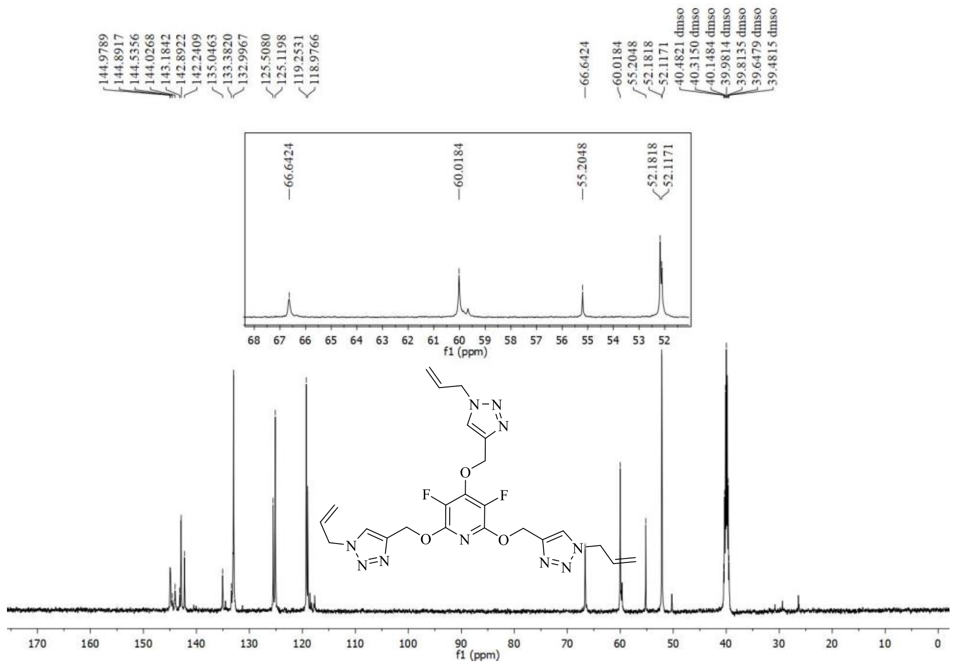

^{13}C NMR spectrum of 2,4,6-tris((1-allyl-1H-1,2,3-triazol-4-yl)methoxy)-3,5-difluoropyridine **7b**

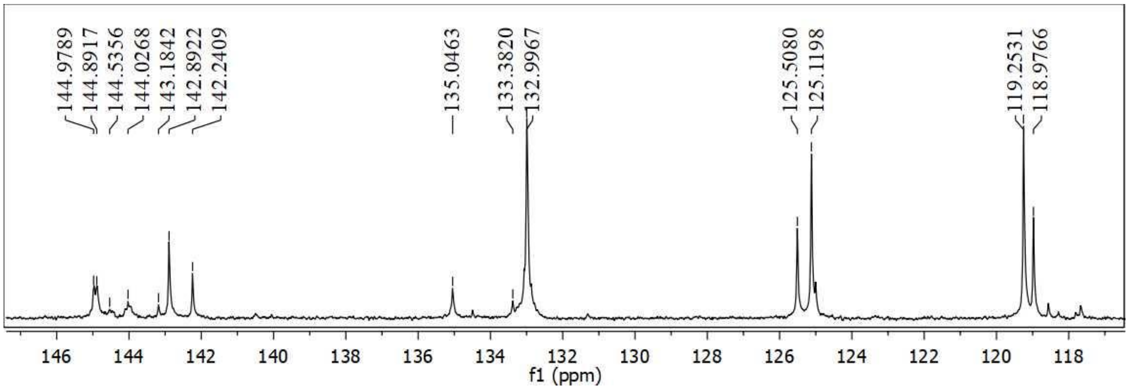

Expanded ^{13}C NMR spectrum of 2,4,6-tris((1-allyl-1H-1,2,3-triazol-4-yl)methoxy)-3,5-difluoropyridine **7b**

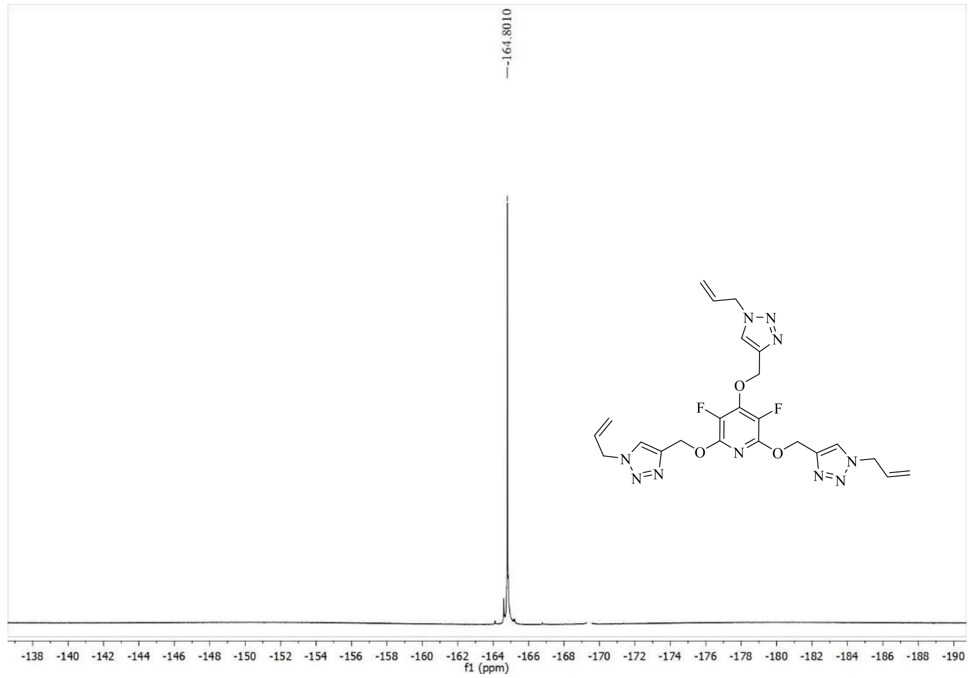

^{19}F NMR spectrum of 2,4,6-tris((1-allyl-1H-1,2,3-triazol-4-yl)methoxy)-3,5-difluoropyridine **7b**

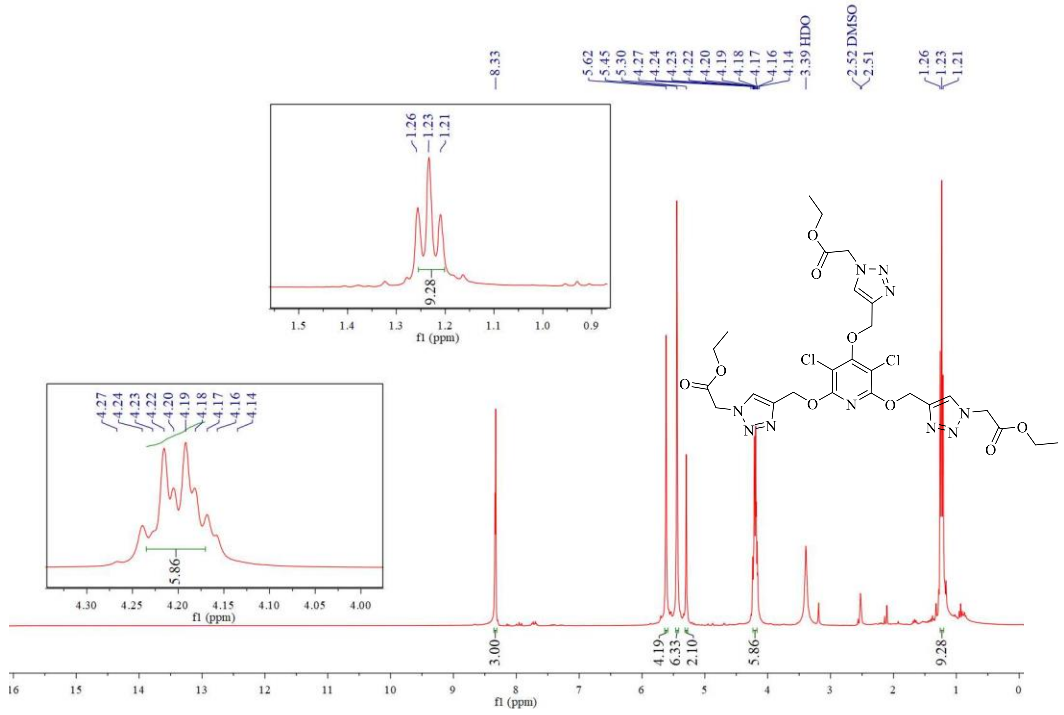

^{1}H NMR spectrum of triethyl 2,2',2''-((((3,5-dichloropyridine-2,4,6-triyl)tris(oxy))tris(methylene))tris(1H-1,2,3-triazole-4,1-diyl))triacetate **7c**

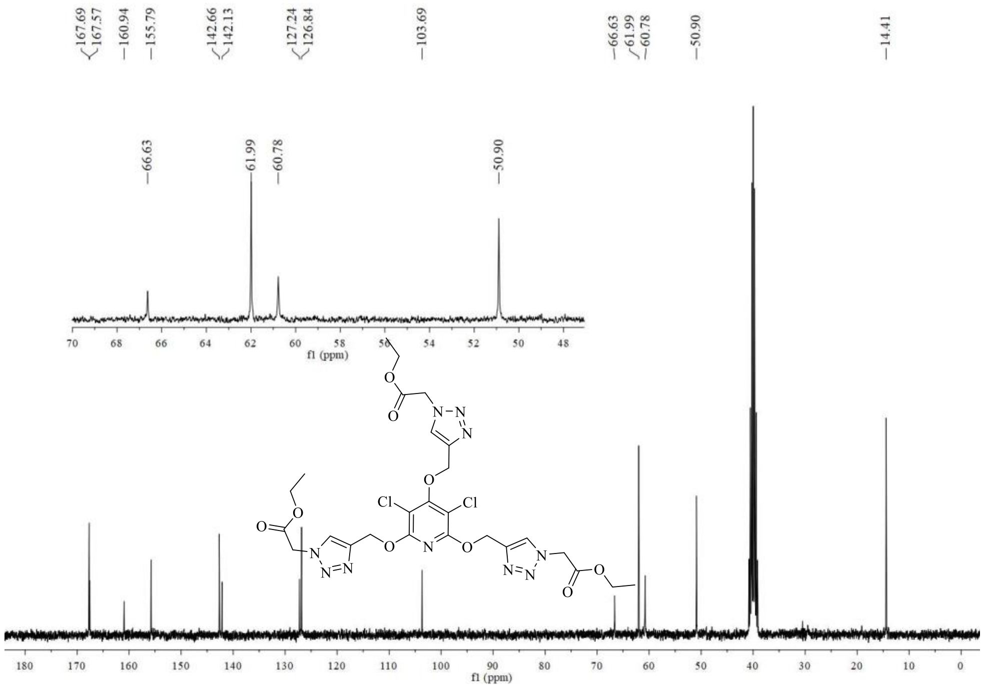

^{13}C NMR spectrum of triethyl 2,2',2''-((((3,5-dichloropyridine-2,4,6-triyl)tris(oxy))tris(methylene))tris(1H-1,2,3-triazole-4,1-diyl))triacetate **7c**

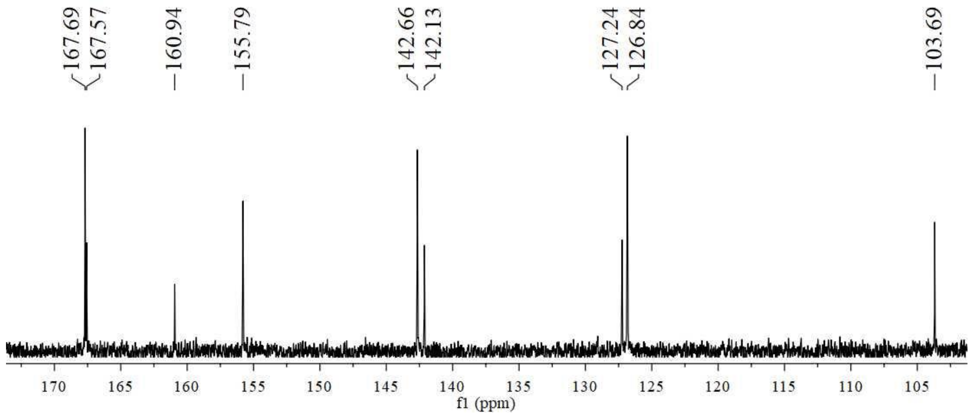

Expanded ^{13}C NMR spectrum of triethyl 2,2',2''-((((3,5-dichloropyridine-2,4,6-triyl)tris(oxy))tris(methylene))tris(1H-1,2,3-triazole-4,1-diyl))triacetate **7c**

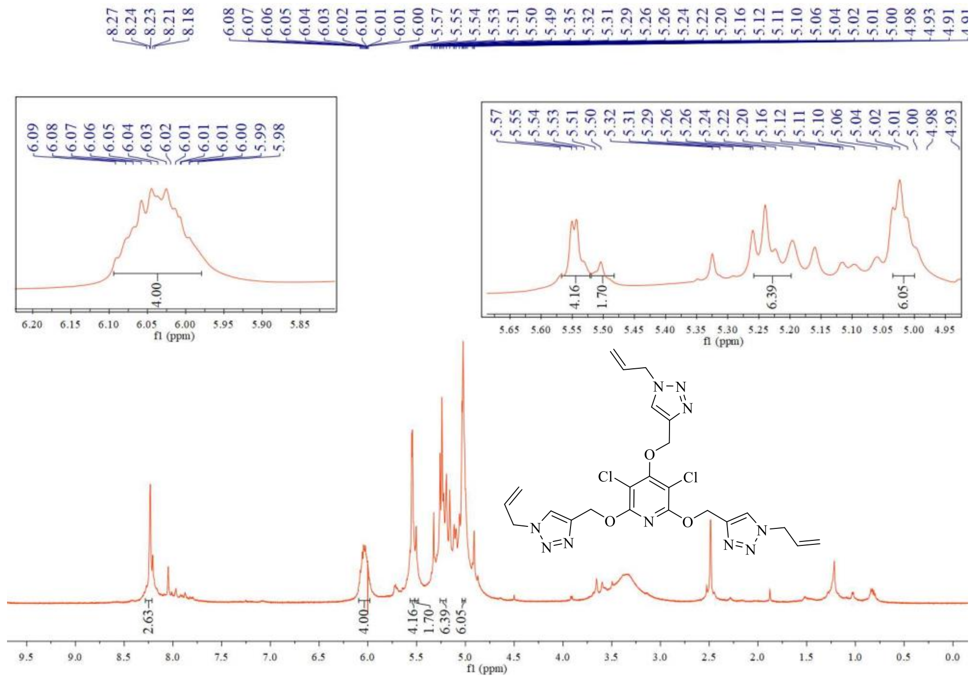

^{1}H NMR spectrum of 2,4,6-tris((1-allyl-1H-1,2,3-triazol-4-yl)methoxy)-3,5-difluoropyridine **7d**

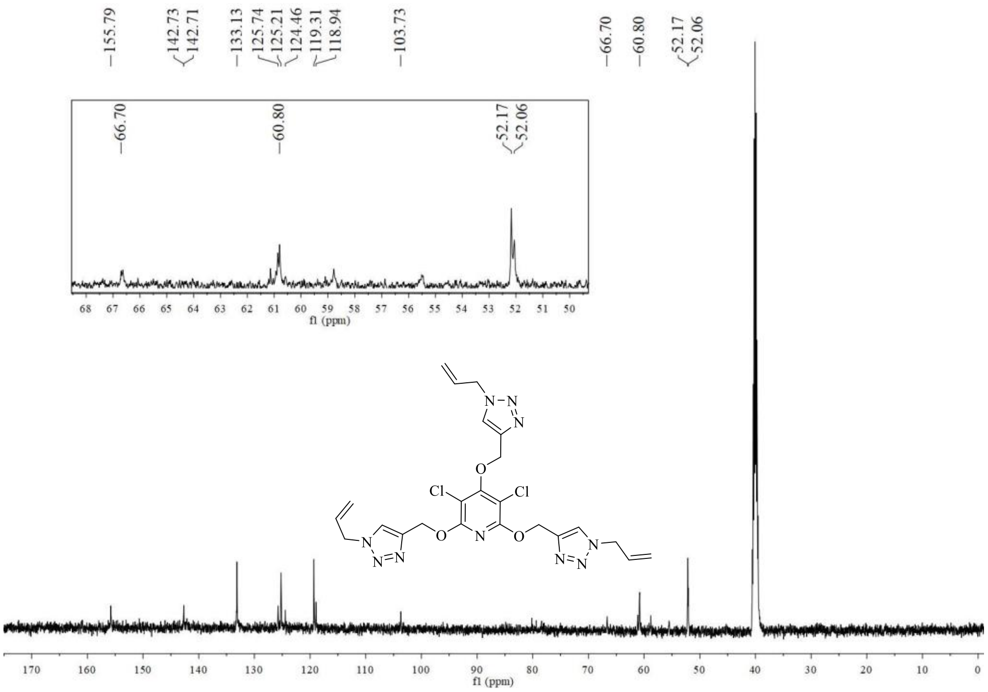

^{13}C NMR spectrum of 2,4,6-tris((1-allyl-1H-1,2,3-triazol-4-yl)methoxy)-3,5-difluoropyridine **7d**

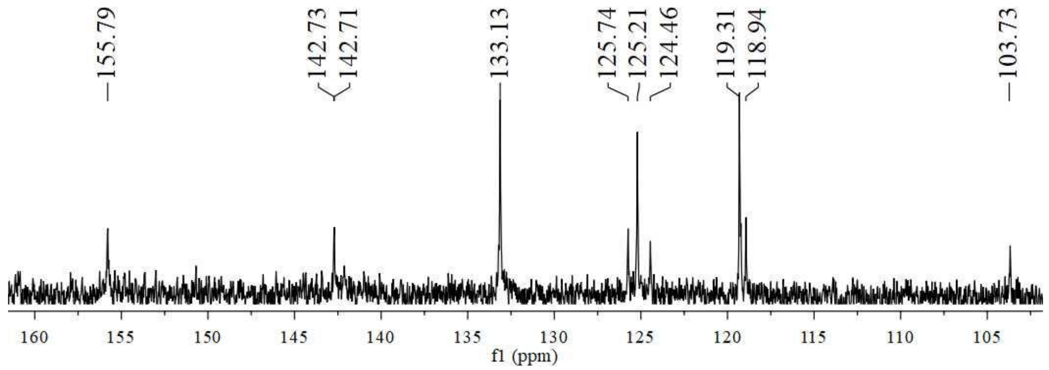

Expanded ^{13}C NMR spectrum of 2,4,6-tris((1-allyl-1H-1,2,3-triazol-4-yl)methoxy)-3,5-difluoropyridine **7d**

7. NMR spectra of 2-((1-benzyl-1H-1,2,3-triazol-4-yl)methoxy)-3,4,5,6-tetrachloropyridine derivatives 8a-c

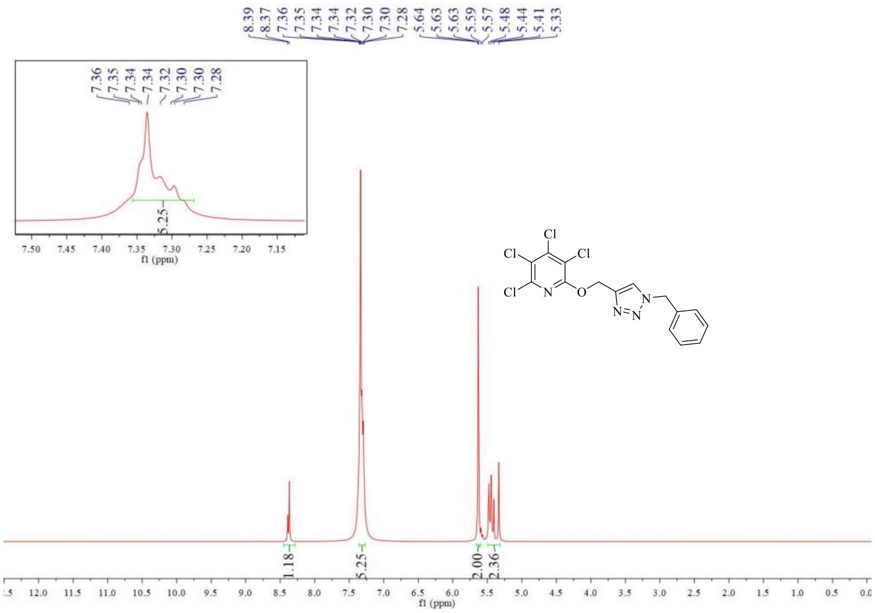

^{1}H NMR spectrum of 2-((1-benzyl-1H-1,2,3-triazol-4-yl)methoxy)-3,4,5,6-tetrachloropyridine **8a**

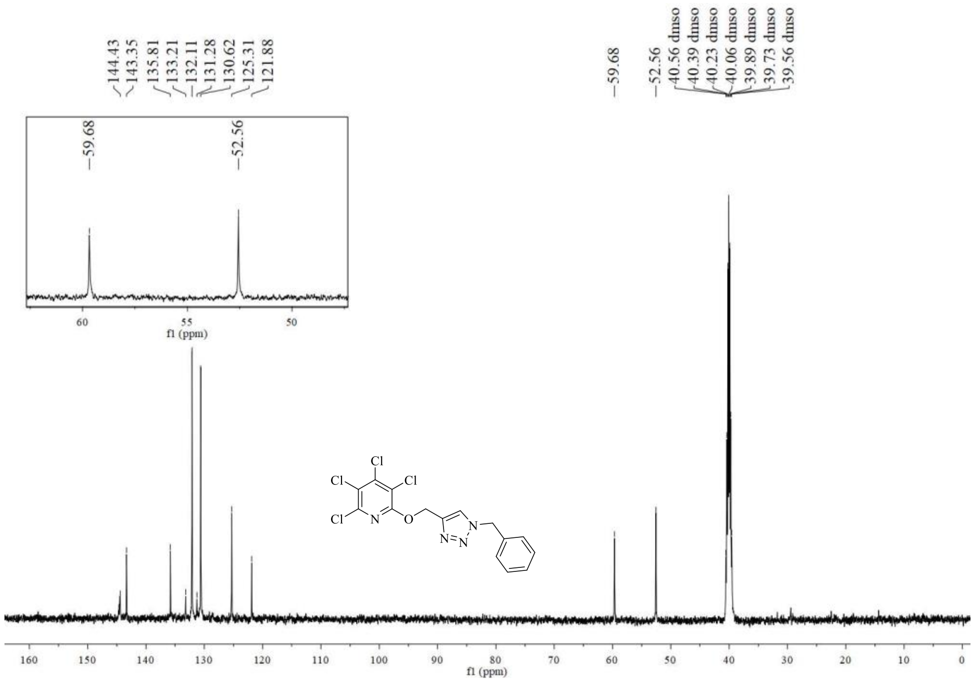

^{13}C NMR spectrum of 2-((1-benzyl-1H-1,2,3-triazol-4-yl)methoxy)-3,4,5,6-tetrachloropyridine **8a**

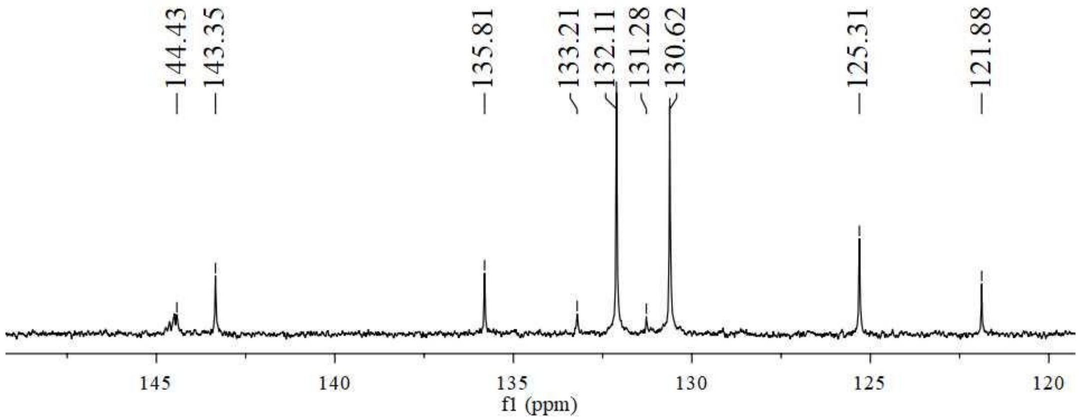

Expanded ^{13}C NMR spectrum of 2-((1-benzyl-1H-1,2,3-triazol-4-yl)methoxy)-3,4,5,6-tetrachloropyridine **8a**

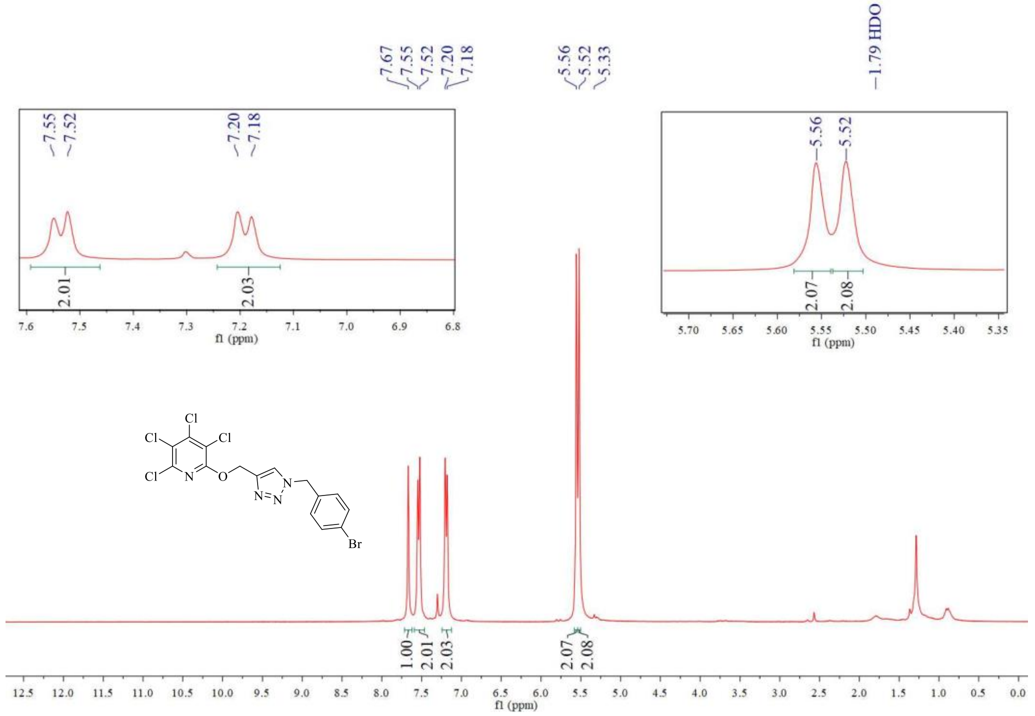

^{1}H NMR spectrum of 2-((1-(4-bromobenzyl)-1H-1,2,3-triazol-4-yl)methoxy)-3,4,5,6-tetrachloropyridine **8b**

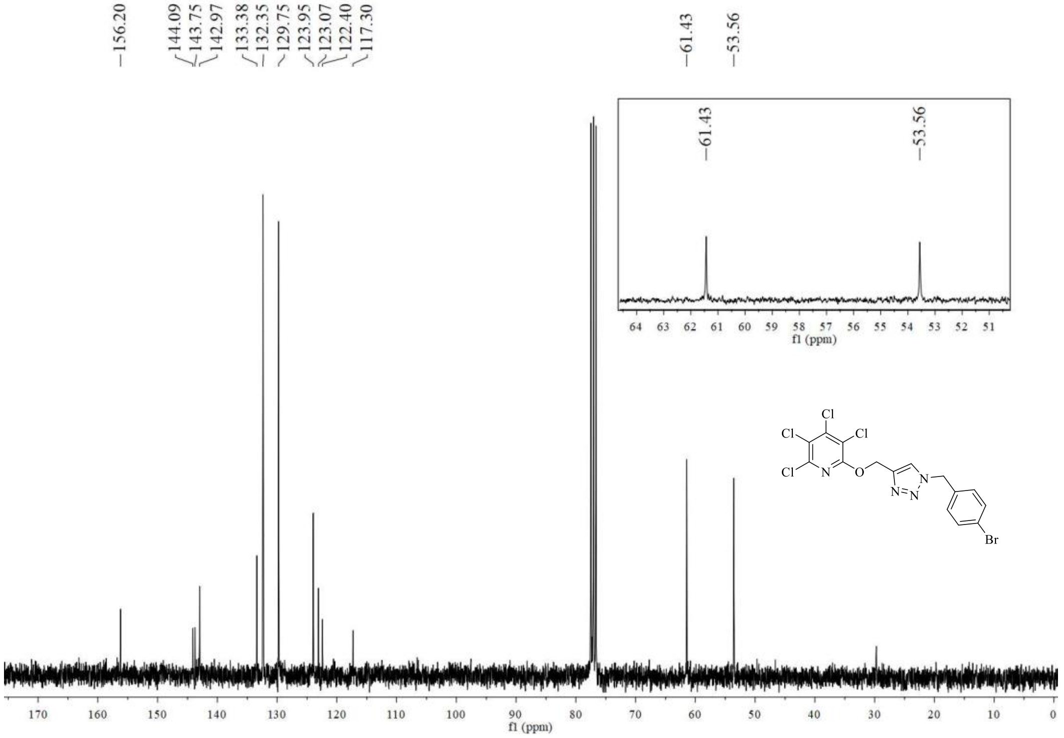

^{13}C NMR spectrum of 2-((1-(4-bromobenzyl)-1H-1,2,3-triazol-4-yl)methoxy)-3,4,5,6-tetrachloropyridine **8b**

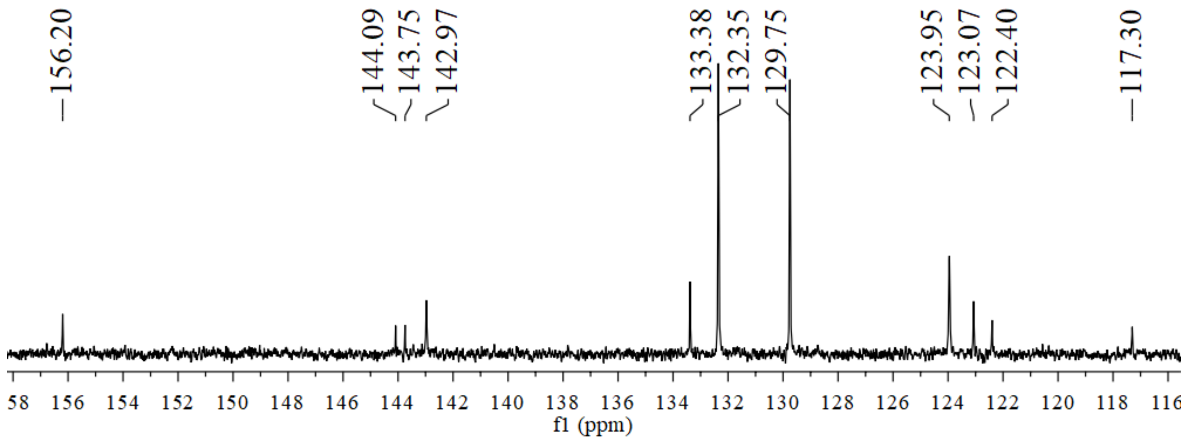

Expanded ^{13}C NMR spectrum of 2-((1-(4-bromobenzyl)-1H-1,2,3-triazol-4-yl)methoxy)-3,4,5,6-tetrachloropyridine **8b**

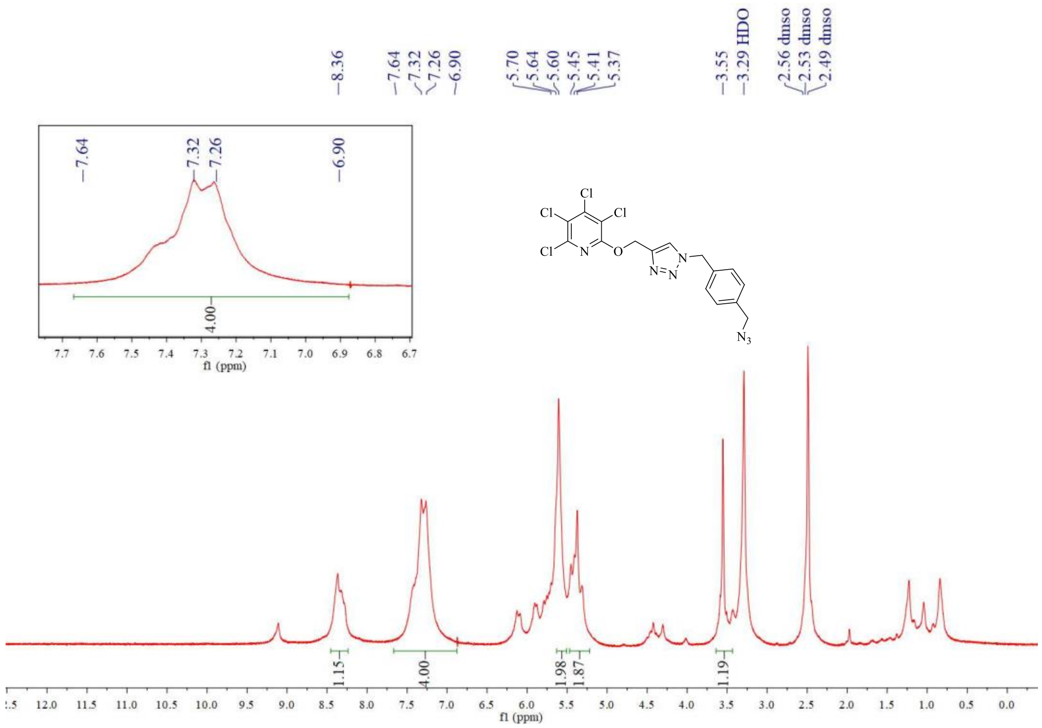

^{1}H NMR spectrum of 2-((1-(4-bromobenzyl)-1H-1,2,3-triazol-4-yl)methoxy)-3,4,5,6-tetrachloropyridine **8c**

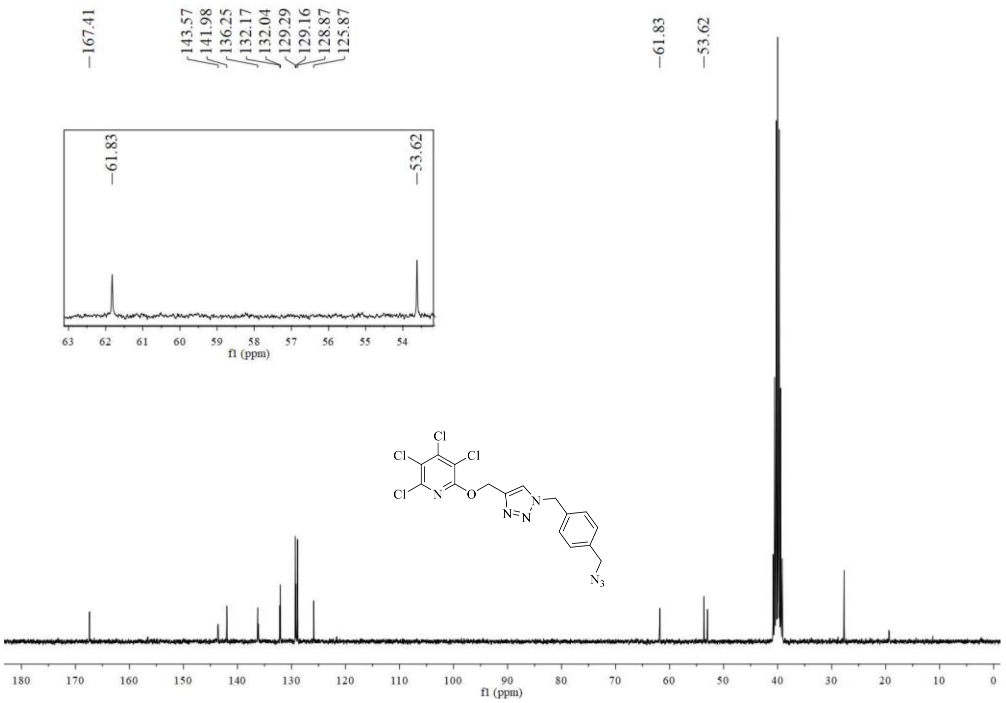

^{13}C NMR spectrum of 2-((1-(4-bromobenzyl)-1H-1,2,3-triazol-4-yl)methoxy)-3,4,5,6-tetrachloropyridine **8c**

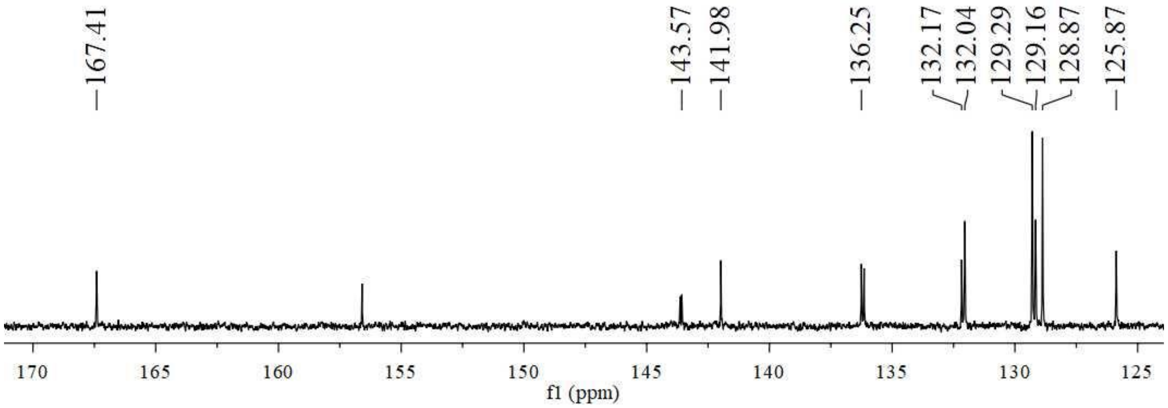

Expanded ^{13}C NMR spectrum of 2-((1-(4-bromobenzyl)-1H-1,2,3-triazol-4-yl)methoxy)-3,4,5,6-tetrachloropyridine **8c**

8. NMR spectra of Suzuki-Miyaura cross-coupling products 10a-f

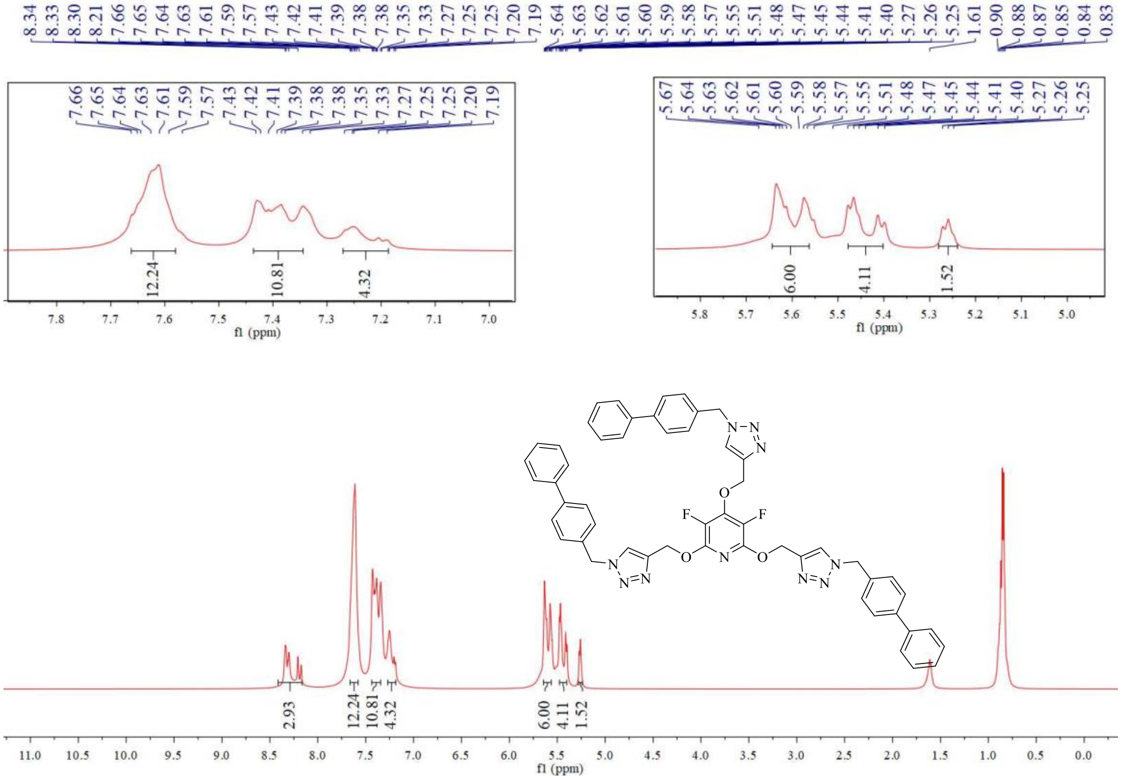

^{1}H NMR spectrum of 2,4,6-tris((1-([1,1'-biphenyl]-4-ylmethyl)-1H-1,2,3-triazol-4-yl)methoxy)-3,5-difluoropyridine **10a**

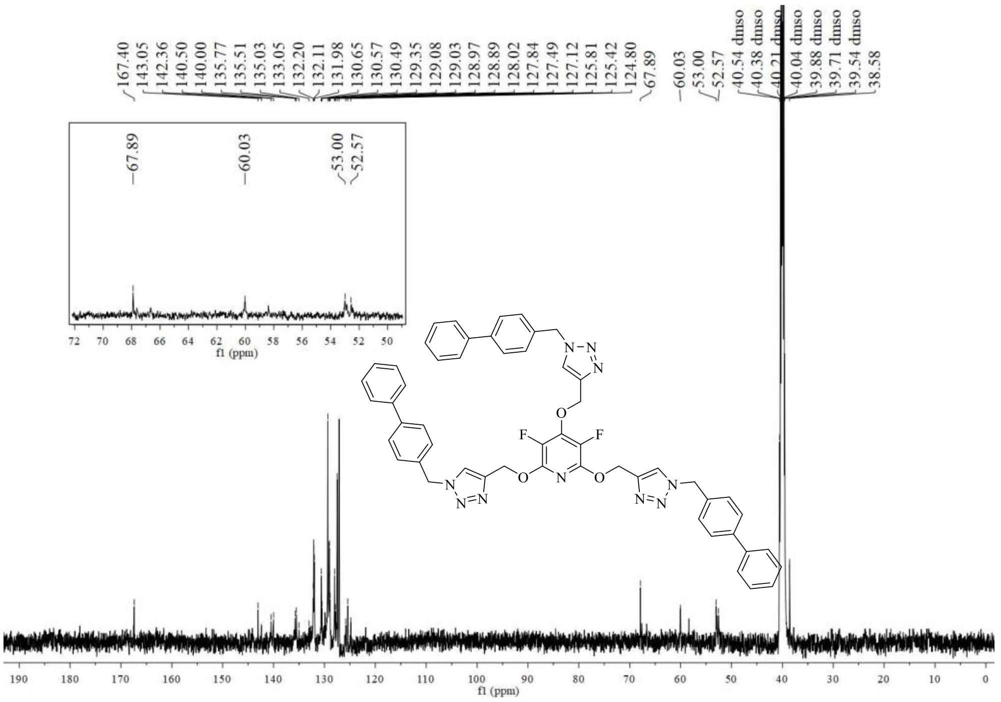

^{13}C NMR spectrum of 2,4,6-tris((1-([1,1'-biphenyl]-4-ylmethyl)-1H-1,2,3-triazol-4-yl)methoxy)-3,5-difluoropyridine **10a**

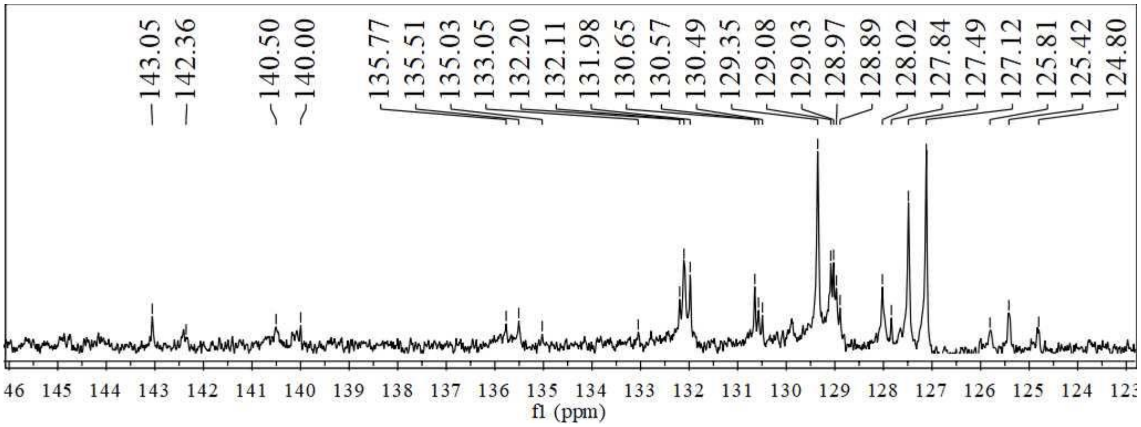

Expanded ^{13}C NMR spectrum of 2,4,6-tris((1-([1,1'-biphenyl]-4-ylmethyl)-1H-1,2,3-triazol-4-yl)methoxy)-3,5-difluoropyridine **10a**

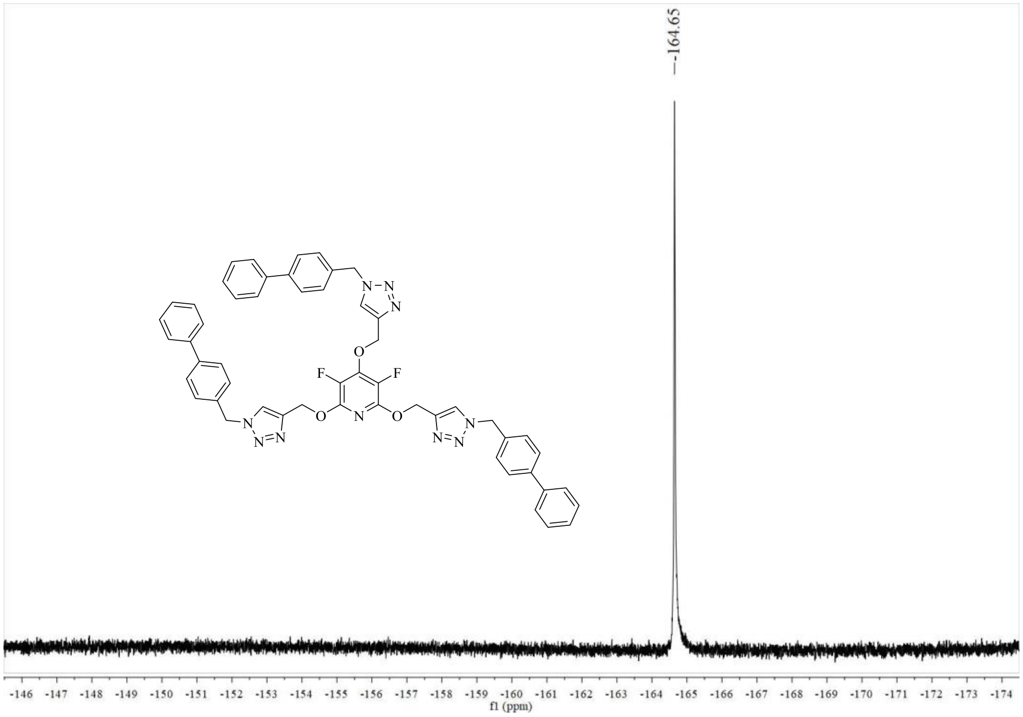

^{19}F NMR spectrum of 2,4,6-tris((1-([1,1'-biphenyl]-4-ylmethyl)-1H-1,2,3-triazol-4-yl)methoxy)-3,5-difluoropyridine **10a**

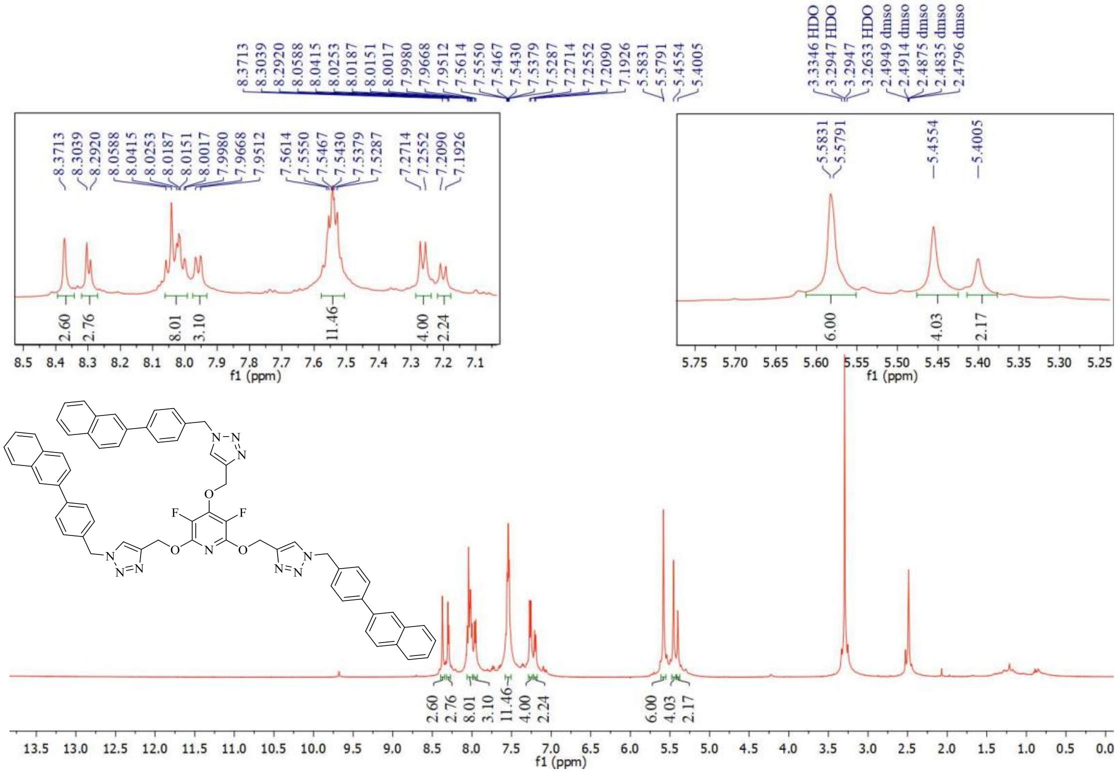

^{1}H NMR spectrum of 3,5-difluoro-2,4,6-tris((1-(4-(naphthalen-2-yl)benzyl)-1H-1,2,3-triazol-4-yl)methoxy)pyridine **10b**

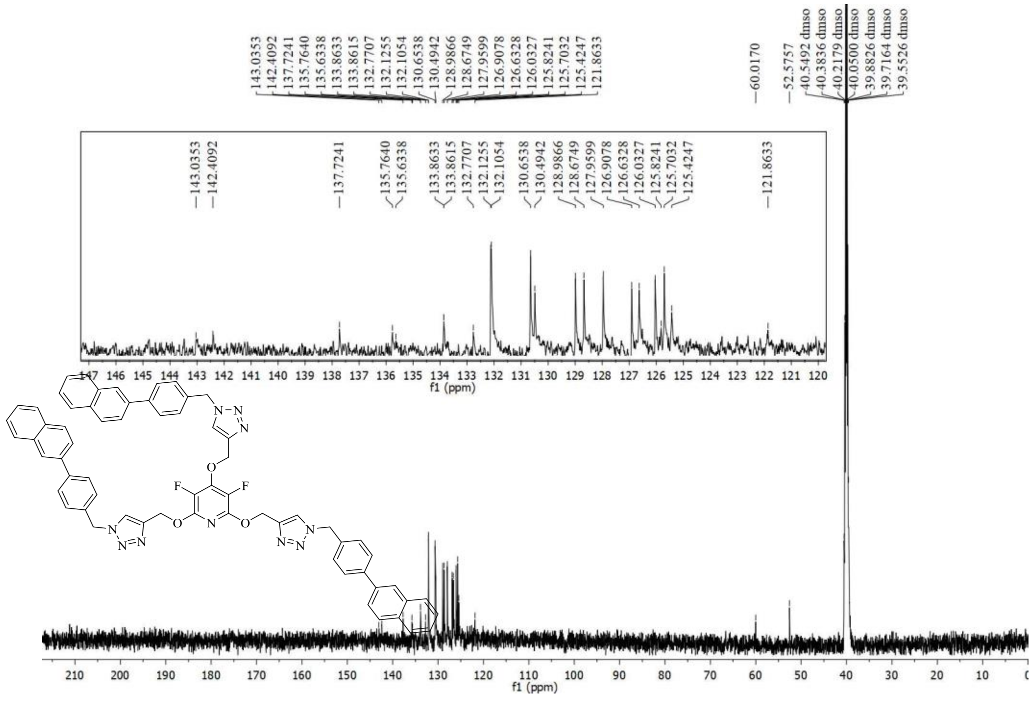

^{13}C NMR spectrum of 3,5-difluoro-2,4,6-tris((1-(4-(naphthalen-2-yl)benzyl)-1H-1,2,3-triazol-4-yl)methoxy)pyridine **10b**

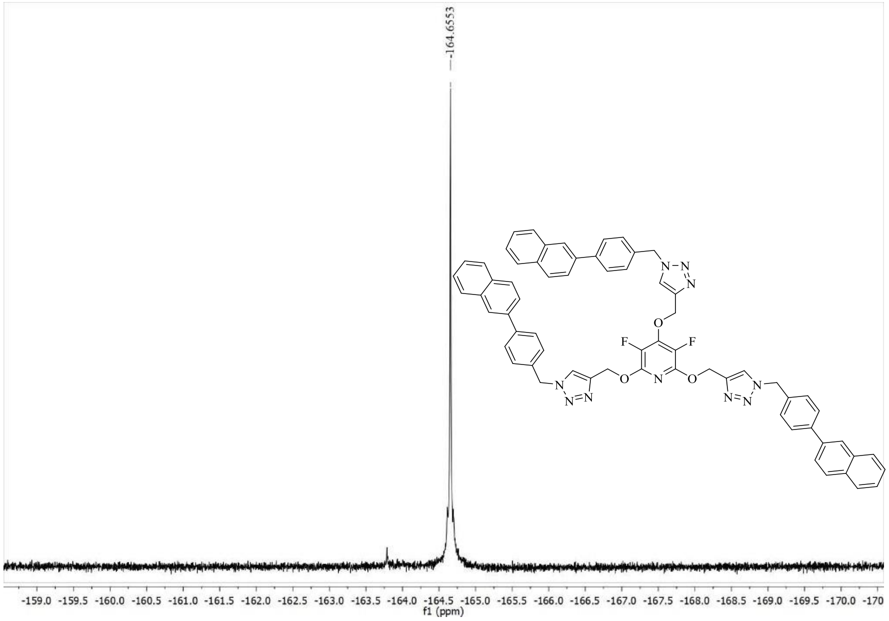

^{13}C NMR spectrum of 3,5-difluoro-2,4,6-tris((1-(4-(naphthalen-2-yl)benzyl)-1H-1,2,3-triazol-4-yl)methoxy)pyridine **10b**

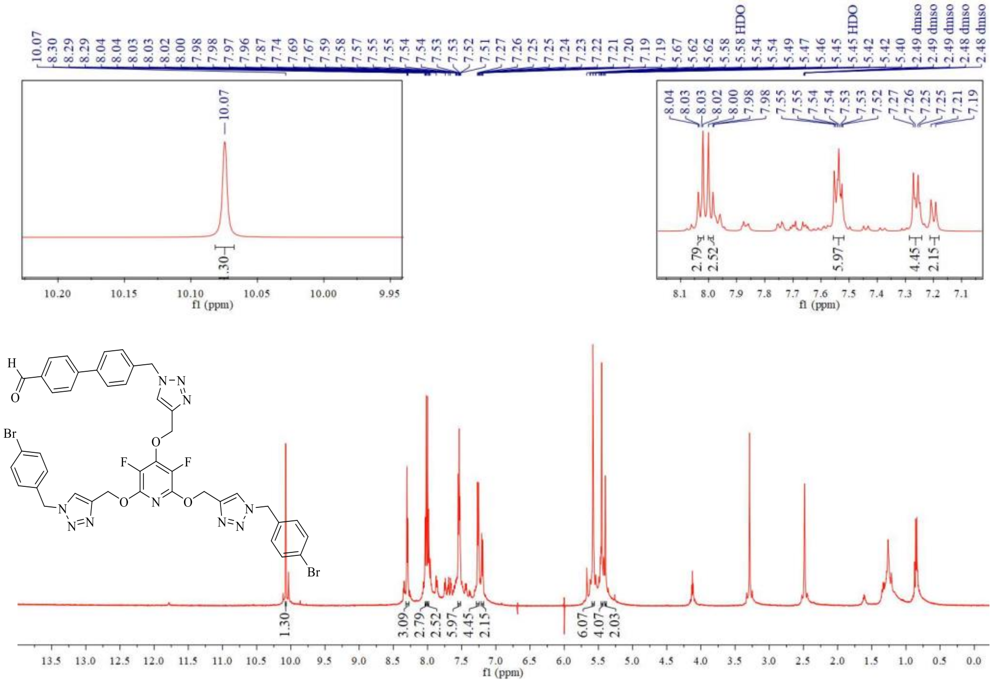

^{1}H NMR spectrum of 4'-((4-(((2,6-bis((1-(4-bromobenzyl)-1H-1,2,3-triazol-4-yl)methoxy)-3,5-difluoropyridin-4-yl)oxy)methyl)-1H-1,2,3-triazol-1-yl)methyl)-[1,1'-biphenyl]-4-carbaldehyde) **10c**

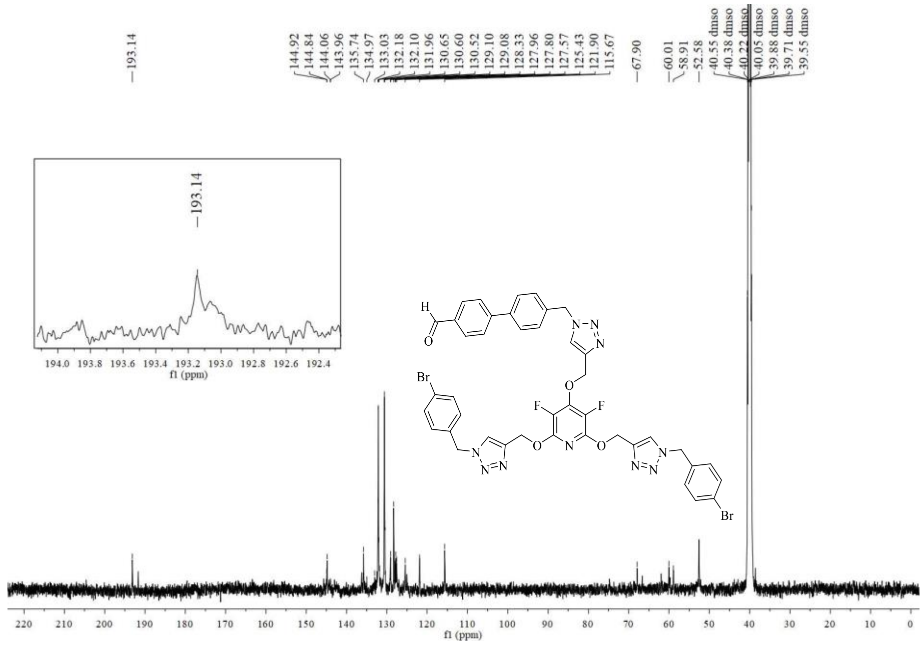

^{13}C NMR spectrum of 4'-((4-(((2,6-bis((1-(4-bromobenzyl)-1H-1,2,3-triazol-4-yl)methoxy)-3,5-difluoropyridin-4-yl)oxy)methyl)-1H-1,2,3-triazol-1-yl)methyl)-[1,1'-biphenyl]-4-carbaldehyde) **10c**

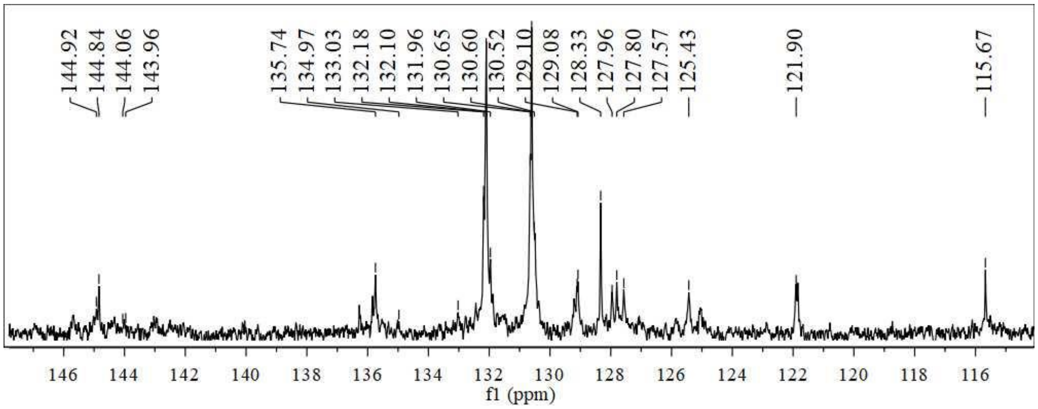

Expanded ^{13}C NMR spectrum of 4'-((4-(((2,6-bis((1-(4-bromobenzyl)-1H-1,2,3-triazol-4-yl)methoxy)-3,5-difluoropyridin-4-yl)oxy)methyl)-1H-1,2,3-triazol-1-yl)methyl)-[1,1'-biphenyl]-4-carbaldehyde) **10c**

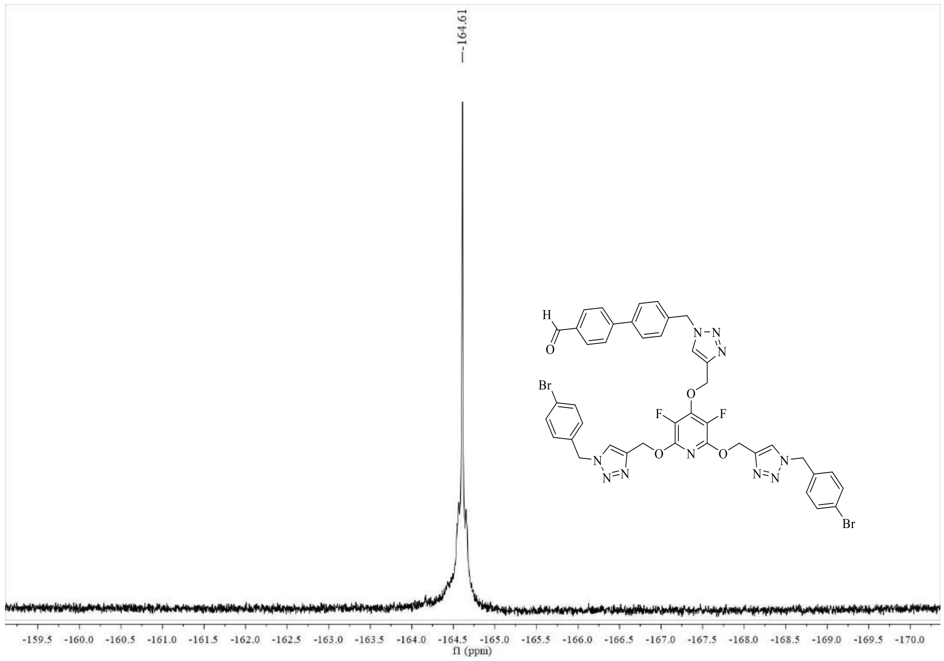

^{19}F NMR spectrum of 4'-((4-(((2,6-bis((1-(4-bromobenzyl)-1H-1,2,3-triazol-4-yl)methoxy)-3,5-difluoropyridin-4-yl)oxy)methyl)-1H-1,2,3-triazol-1-yl)methyl)-[1,1'-biphenyl]-4-carbaldehyde) **10c**

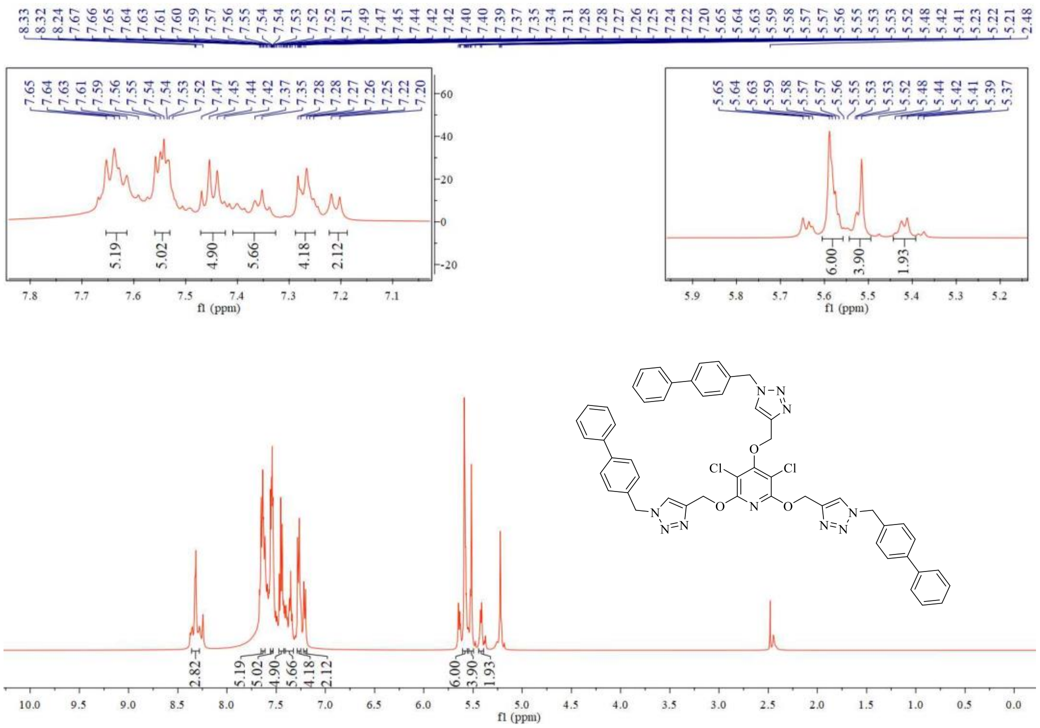

^{1}H NMR spectrum of 2,4,6-tris((1-([1,1'-biphenyl]-4-ylmethyl)-1H-1,2,3-triazol-4-yl)methoxy)-3,5-dichloropyridine **10d**

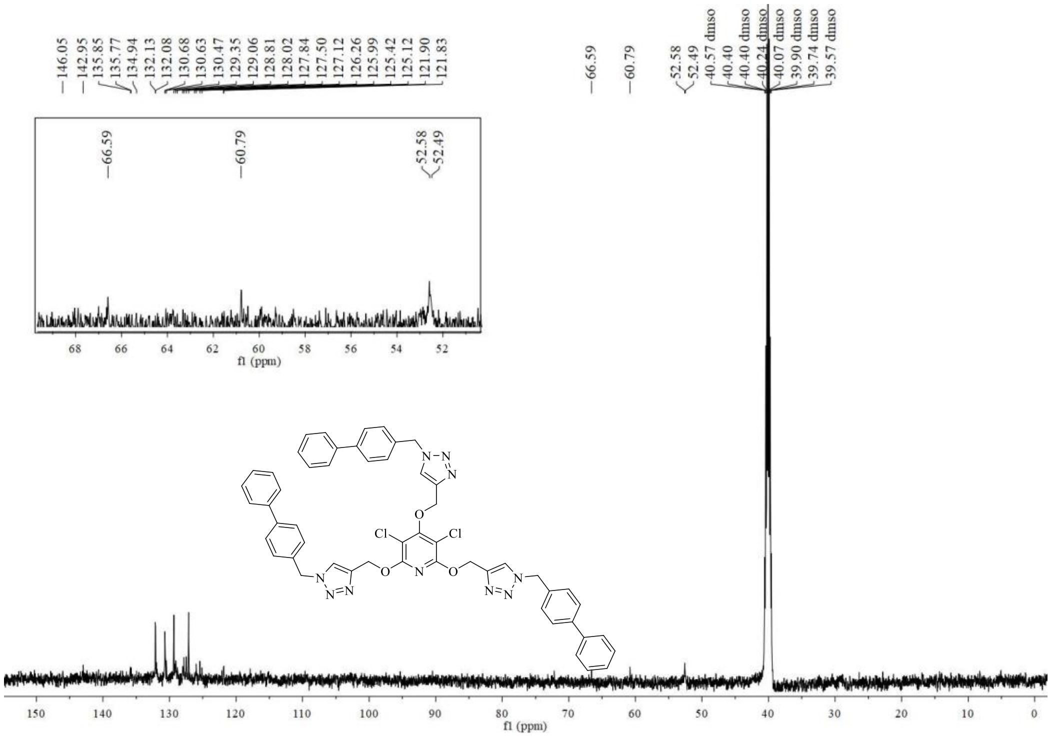

^{13}C NMR spectrum of 2,4,6-tris((1-([1,1'-biphenyl]-4-ylmethyl)-1H-1,2,3-triazol-4-yl)methoxy)-3,5-dichloropyridine **10d**

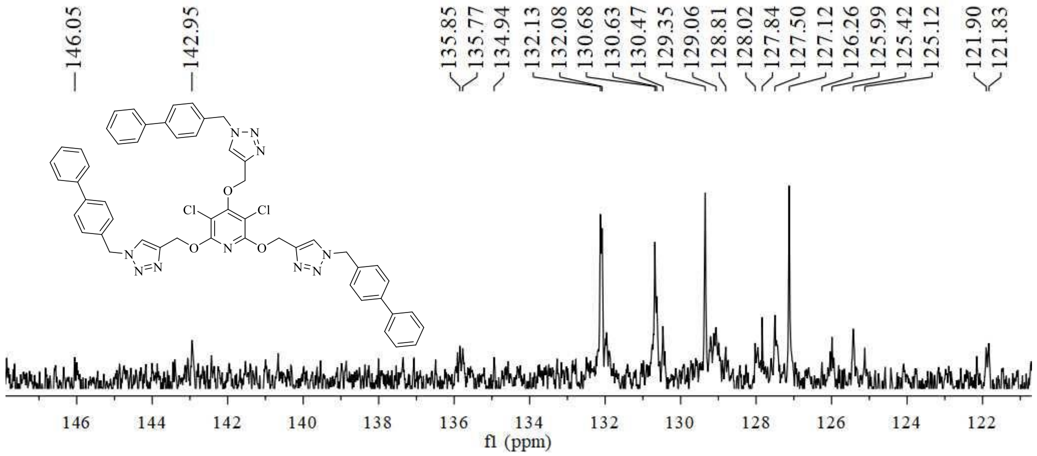

Expanded ^{13}C NMR spectrum of 2,4,6-tris((1-([1,1'-biphenyl]-4-ylmethyl)-1H-1,2,3-triazol-4-yl)methoxy)-3,5-dichloropyridine **10d**

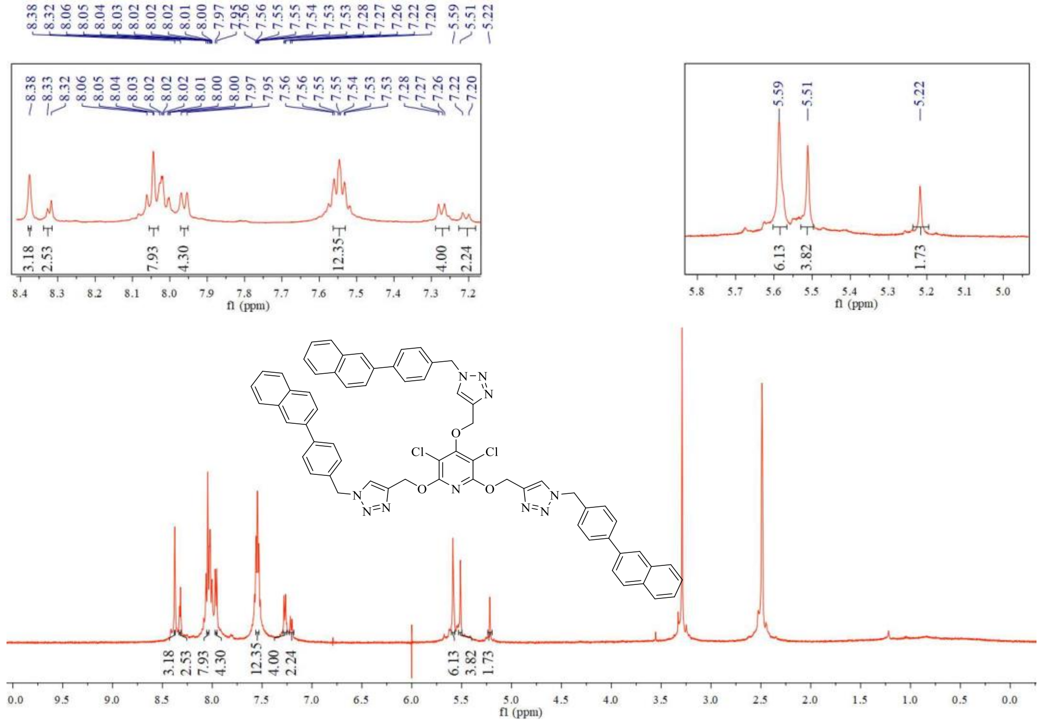

^{1}H NMR spectrum of 3,5-dichloro-2,4,6-tris((1-(4-(naphthalen-2-yl)benzyl)-1H-1,2,3-triazol-4-yl)methoxy)pyridine **10e**

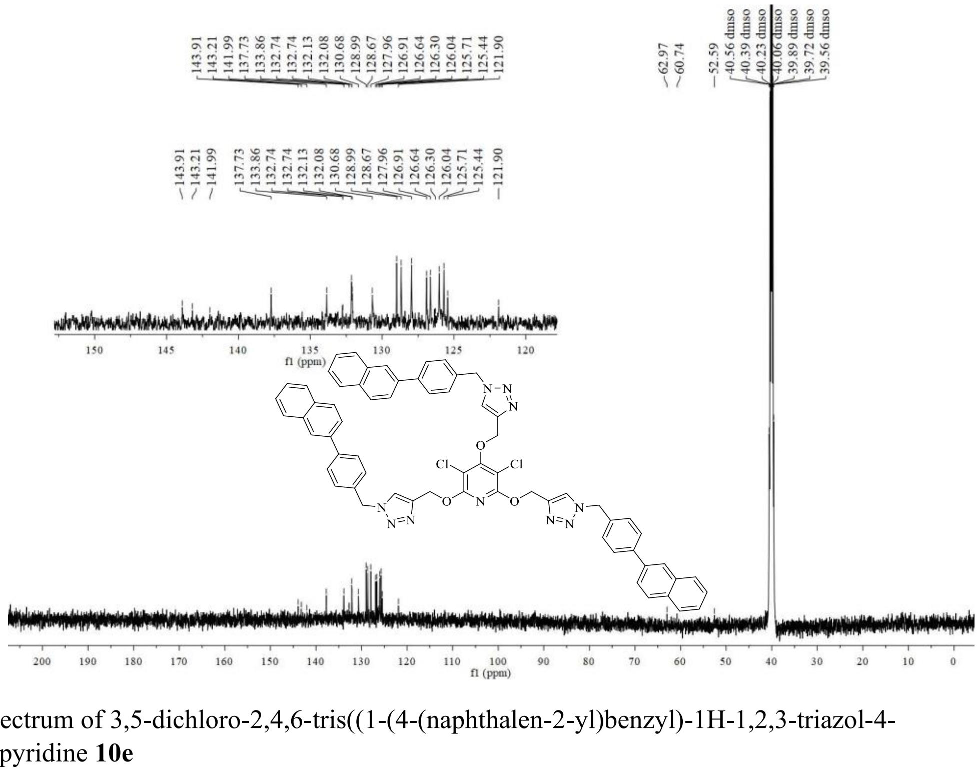

^{13}C NMR spectrum of 3,5-dichloro-2,4,6-tris((1-(4-(naphthalen-2-yl)benzyl)-1H-1,2,3-triazol-4-yl)methoxy)pyridine **10e**

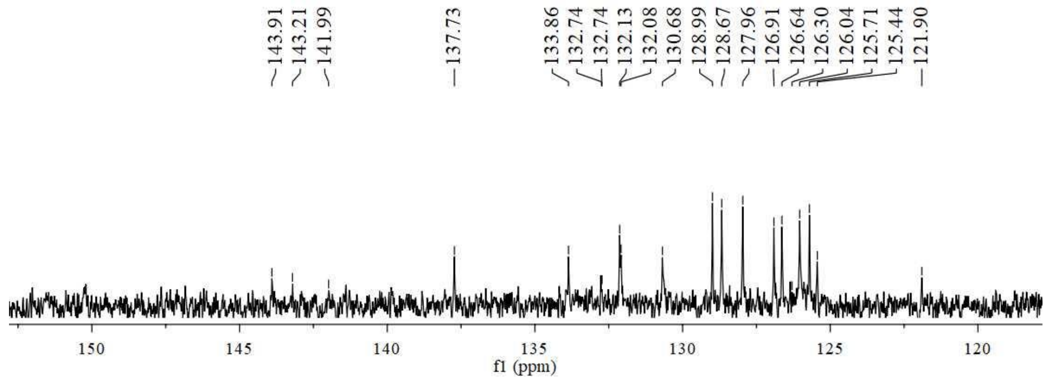

Expanded ^{13}C NMR spectrum of 3,5-dichloro-2,4,6-tris((1-(4-(naphthalen-2-yl)benzyl)-1H-1,2,3-triazol-4-yl)methoxy)pyridine **10e**

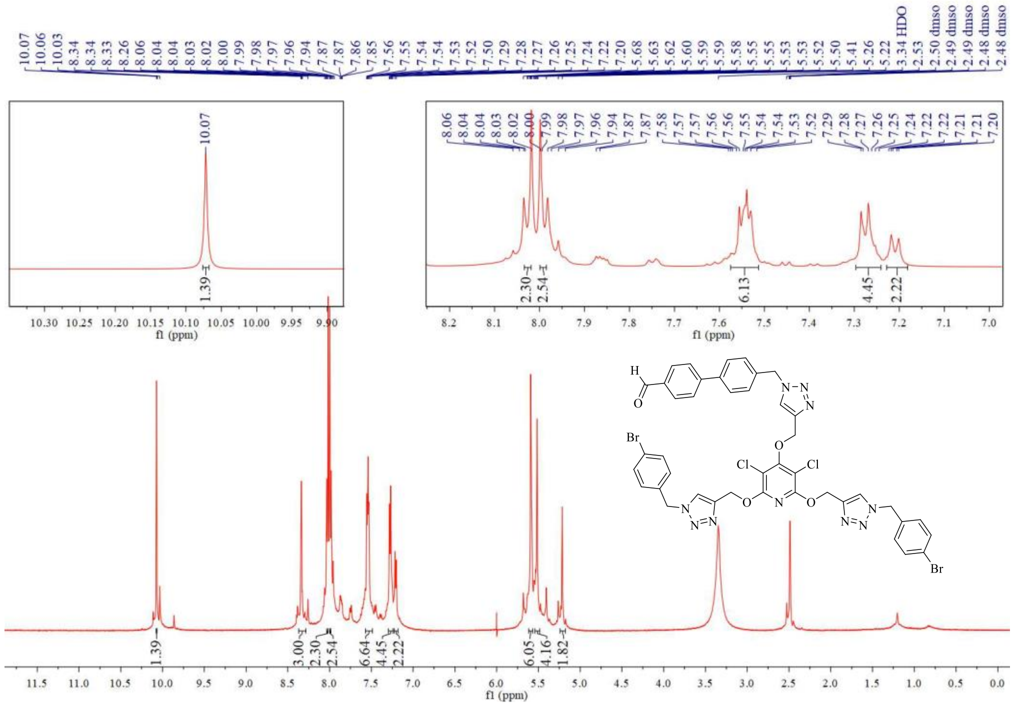

^{1}H NMR spectrum of 4'-((4-(((2,6-bis((1-(4-bromobenzyl)-1H-1,2,3-triazol-4-yl)methoxy)-3,5-dichloropyridin-4-yl)oxy)methyl)-1H-1,2,3-triazol-1-yl)methyl)-[1,1'-biphenyl]-4-carbaldehyde **10f**

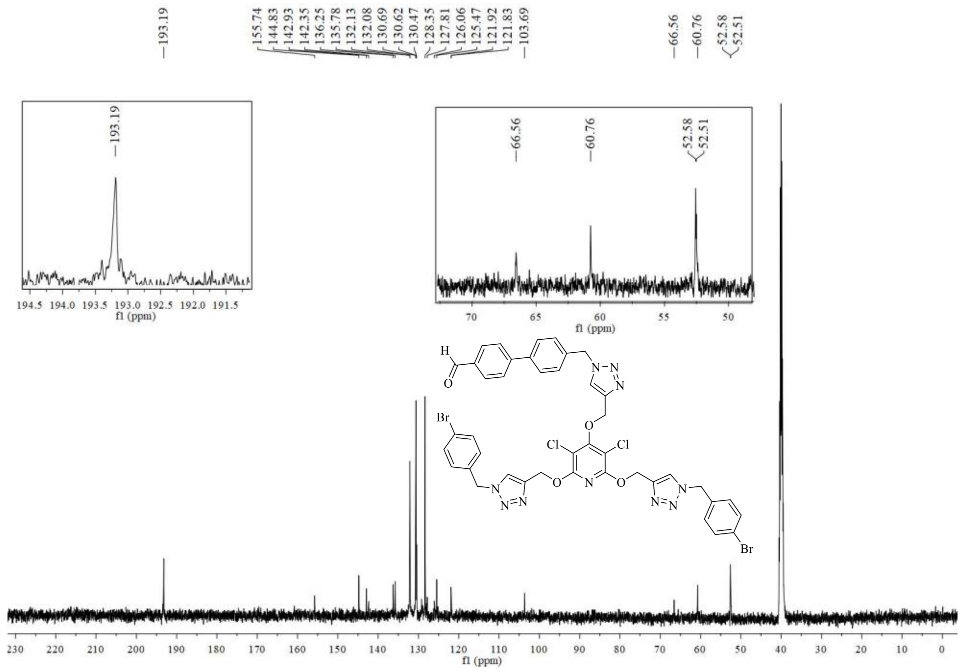

^{13}C NMR spectrum of 4'-((4-(((2,6-bis((1-(4-bromobenzyl)-1H-1,2,3-triazol-4-yl)methoxy)-3,5-dichloropyridin-4-yl)oxy)methyl)-1H-1,2,3-triazol-1-yl)methyl)-[1,1'-biphenyl]-4-carbaldehyde **10f**

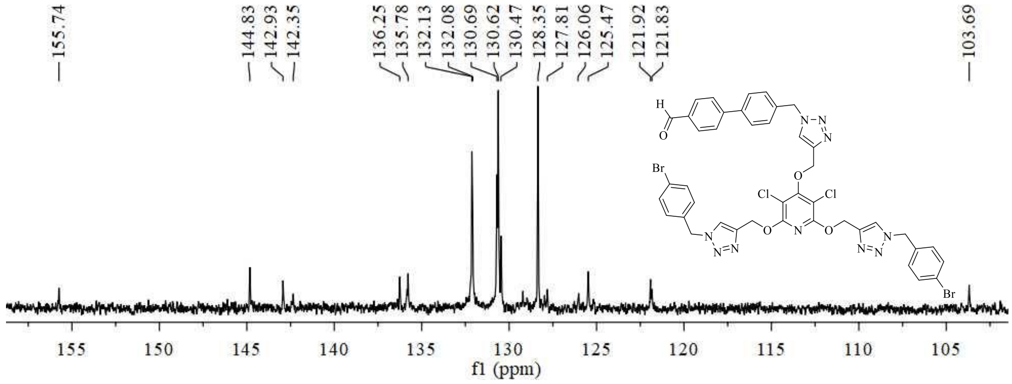

Expanded ^{13}C NMR spectrum of 4'-((4-(((2,6-bis((1-(4-bromobenzyl)-1H-1,2,3-triazol-4-yl)methoxy)-3,5-dichloropyridin-4-yl)oxy)methyl)-1H-1,2,3-triazol-1-yl)methyl)-[1,1'-biphenyl]-4-carbaldehyde **10f**

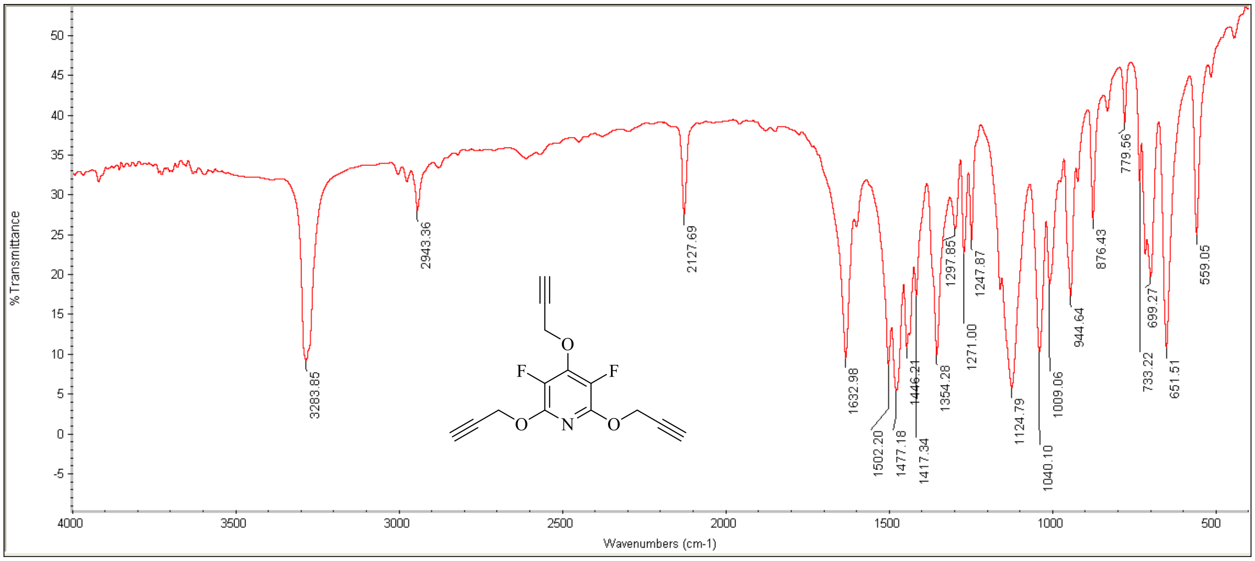

FT-IR spectrum of **3a**

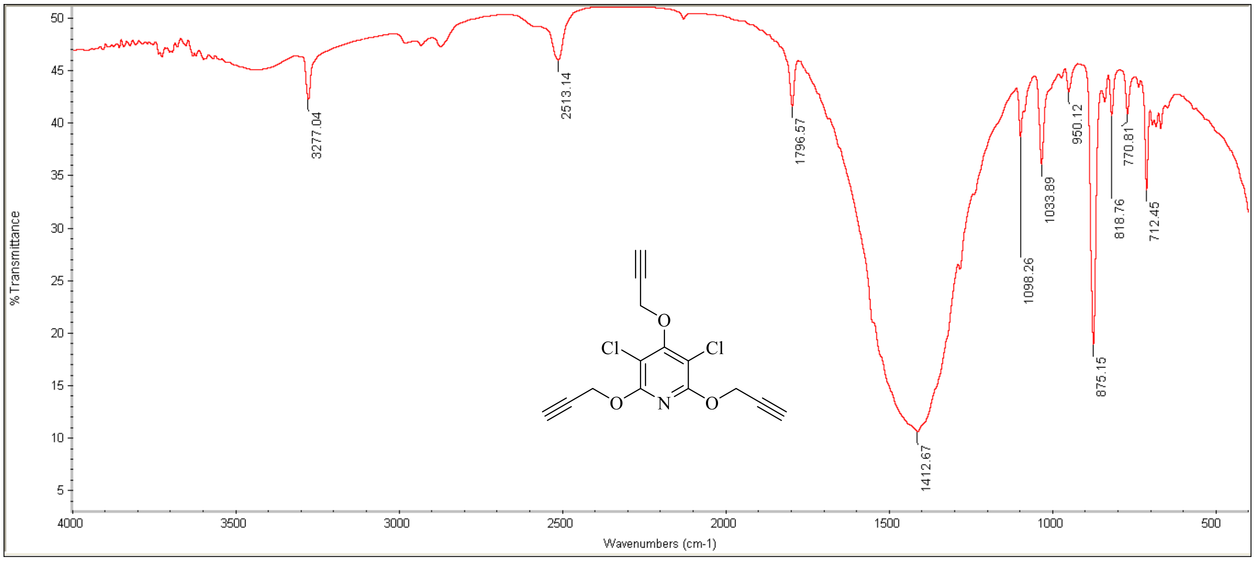

FT-IR spectrum of **3b**

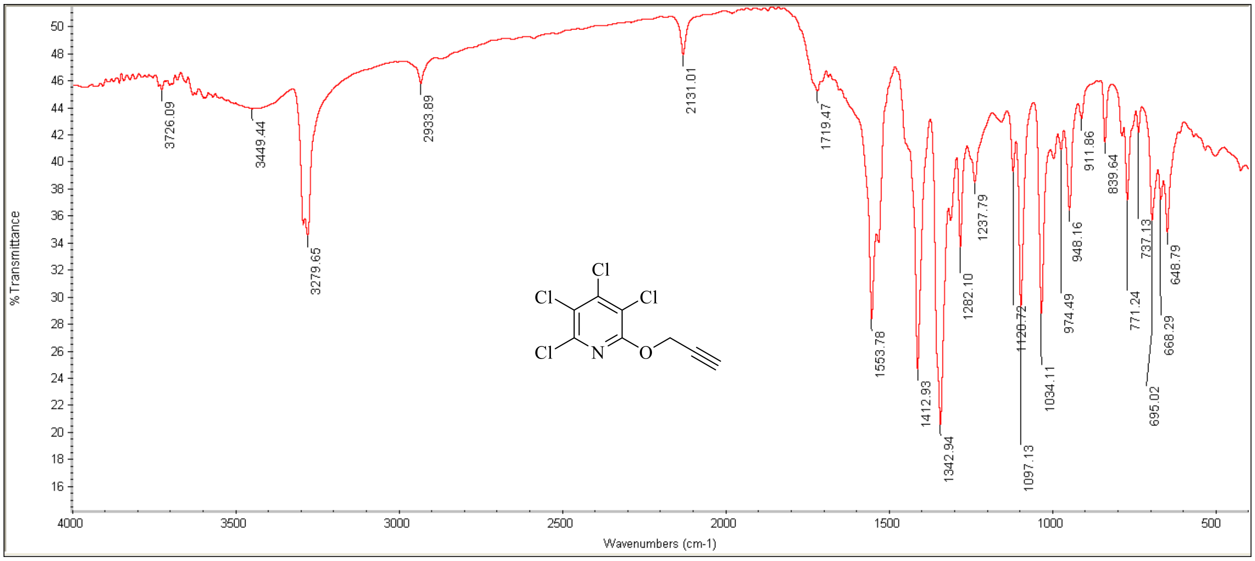

FT-IR spectrum of **3c**

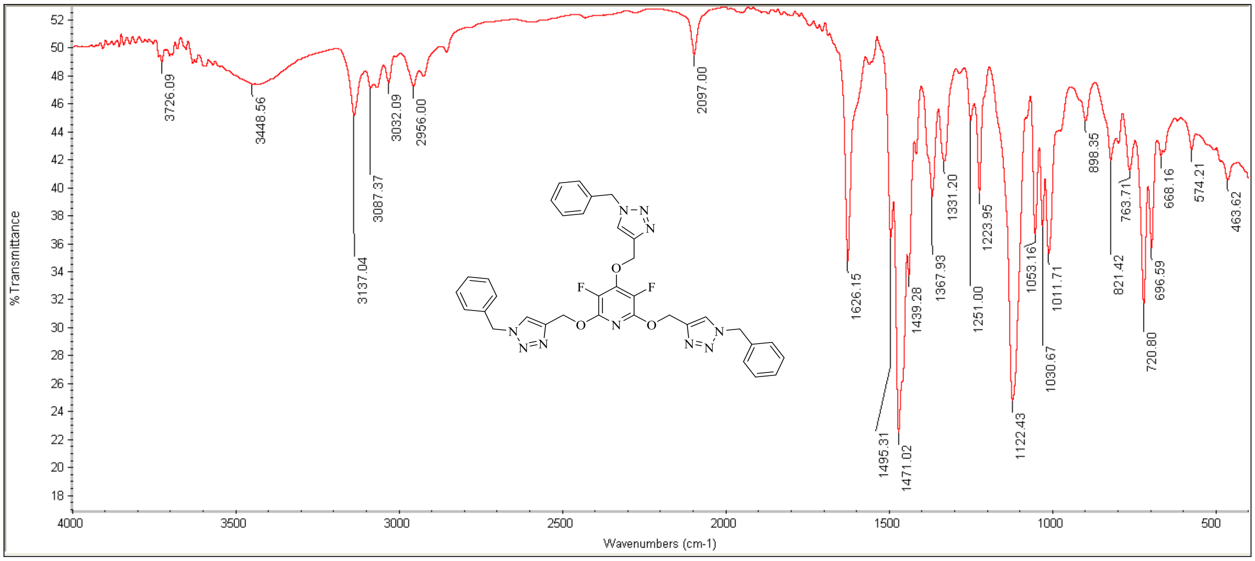

FT-IR spectrum of **5a**

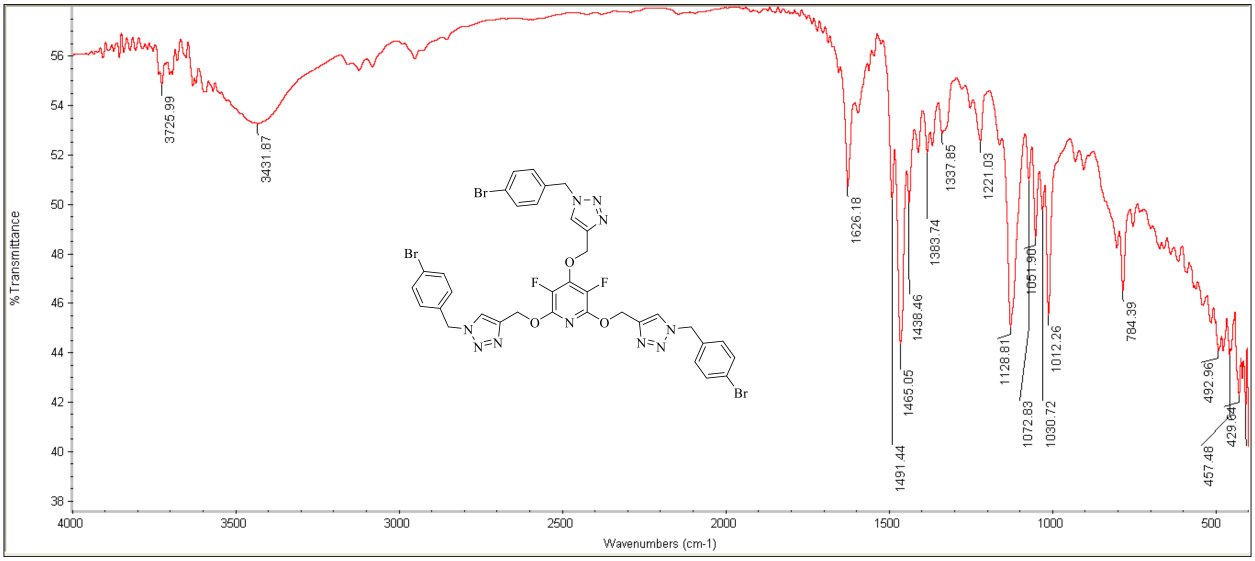

FT-IR spectrum of **5b**

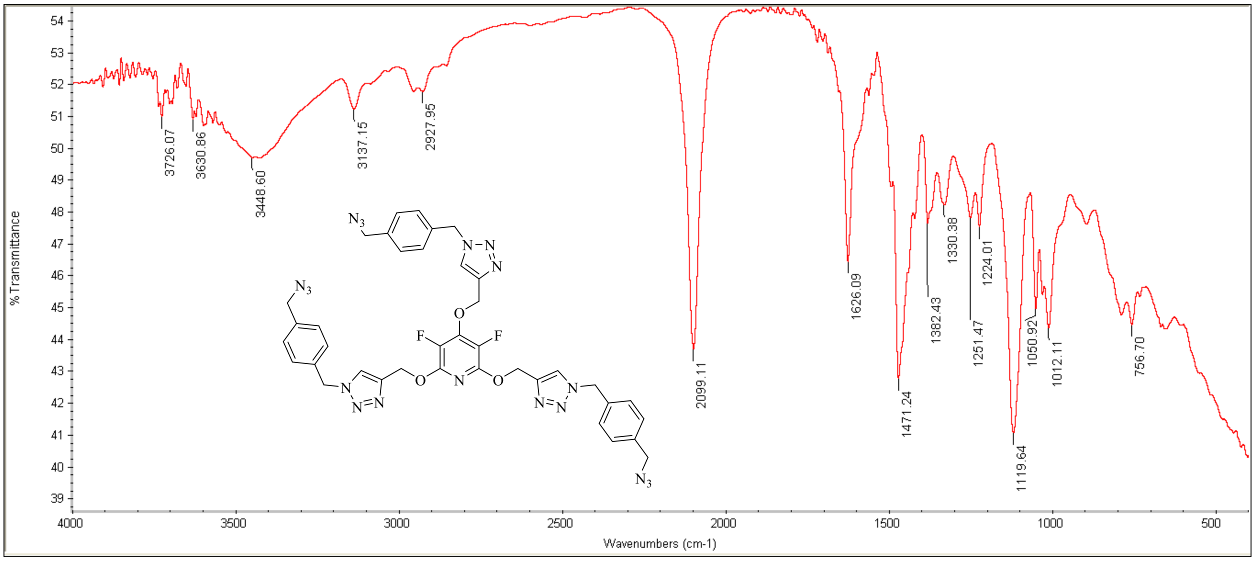

FT-IR spectrum of **5c**

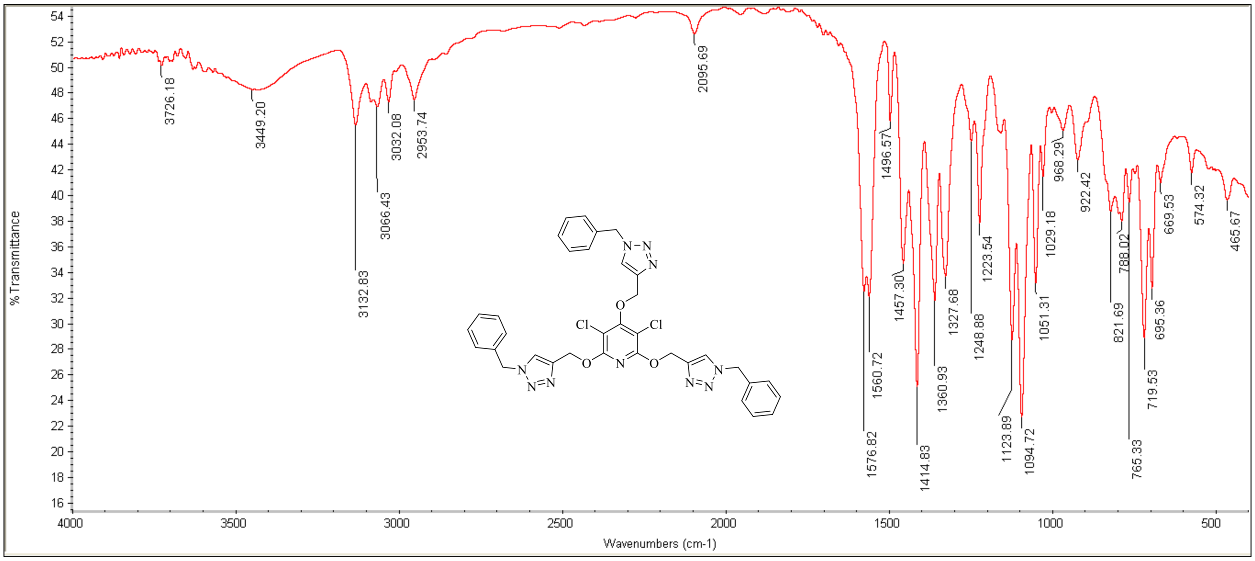

FT-IR spectrum of **5d**

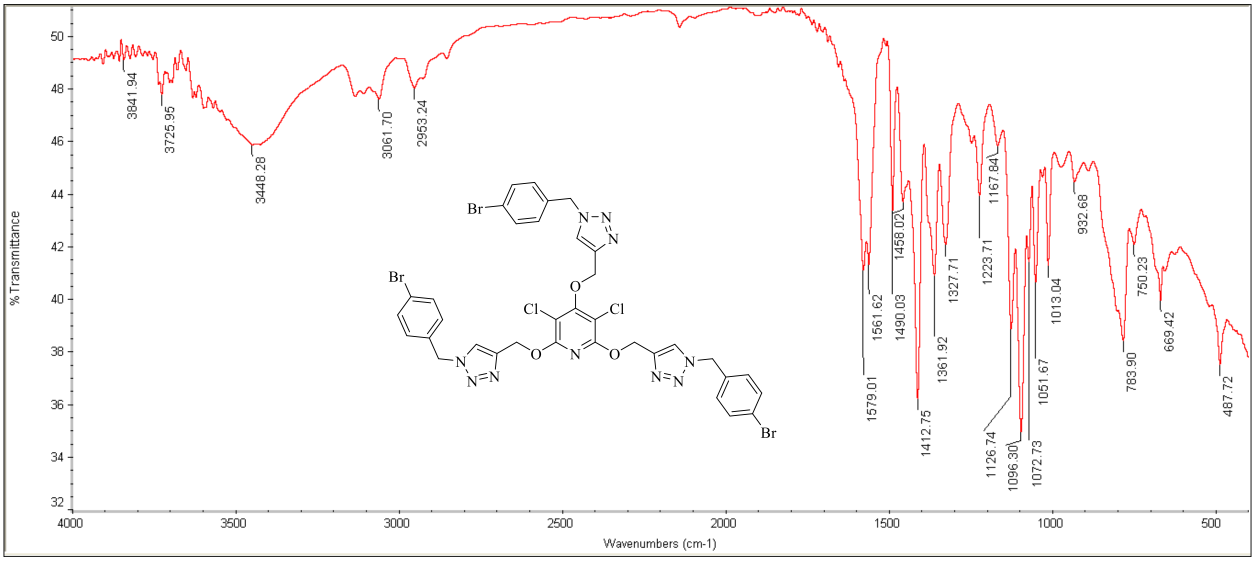

FT-IR spectrum of **5e**

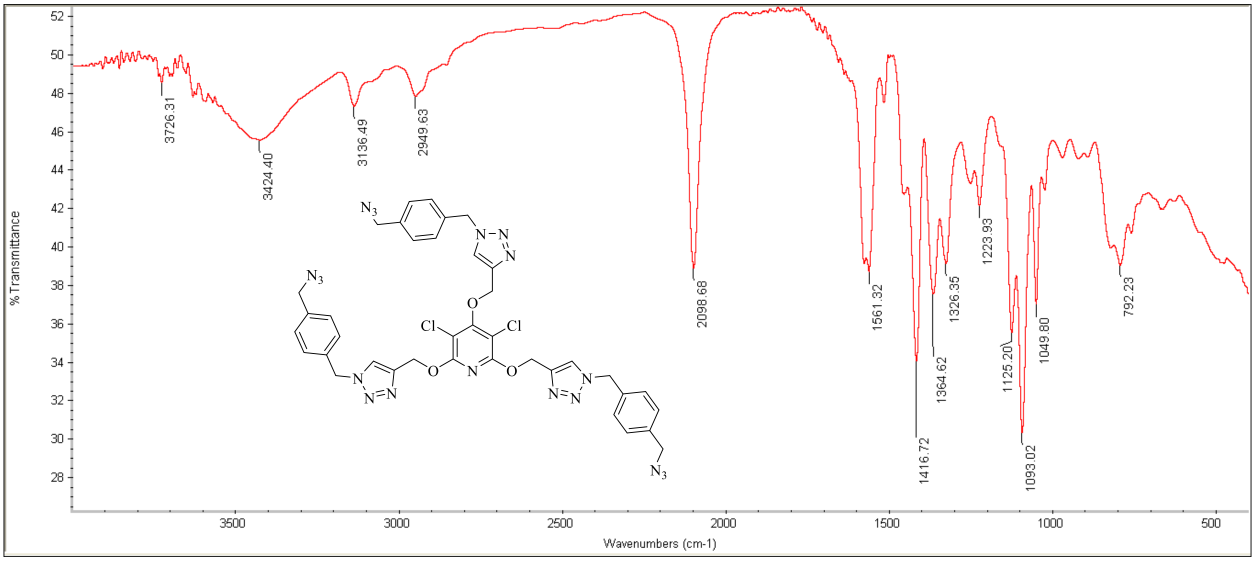

FT-IR spectrum of **5f**

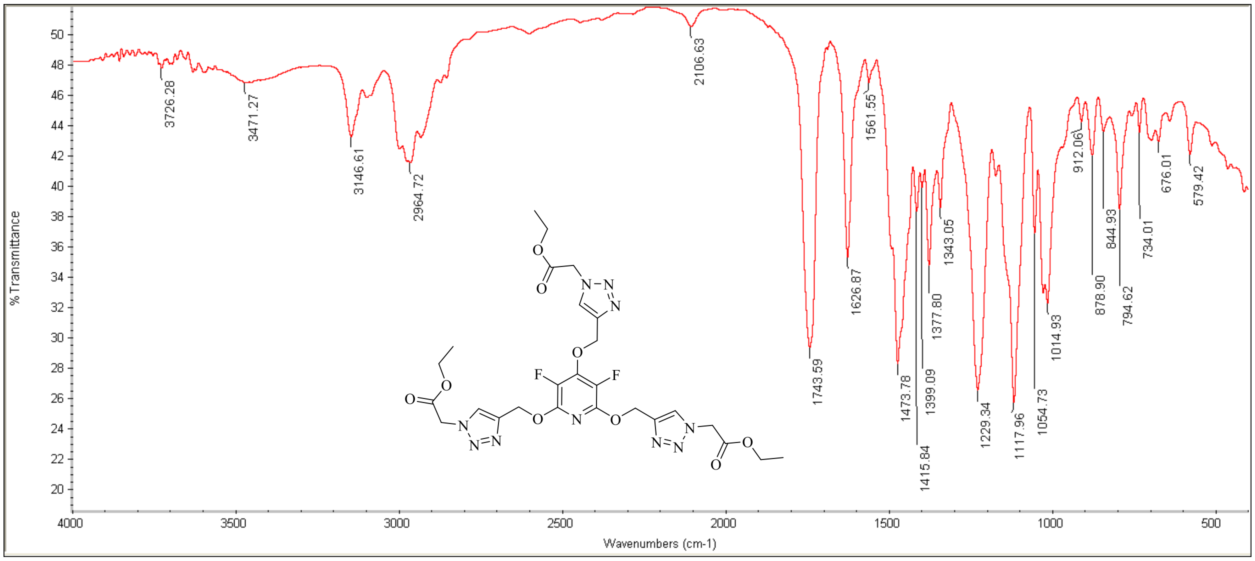

FT-IR spectrum of **7a**

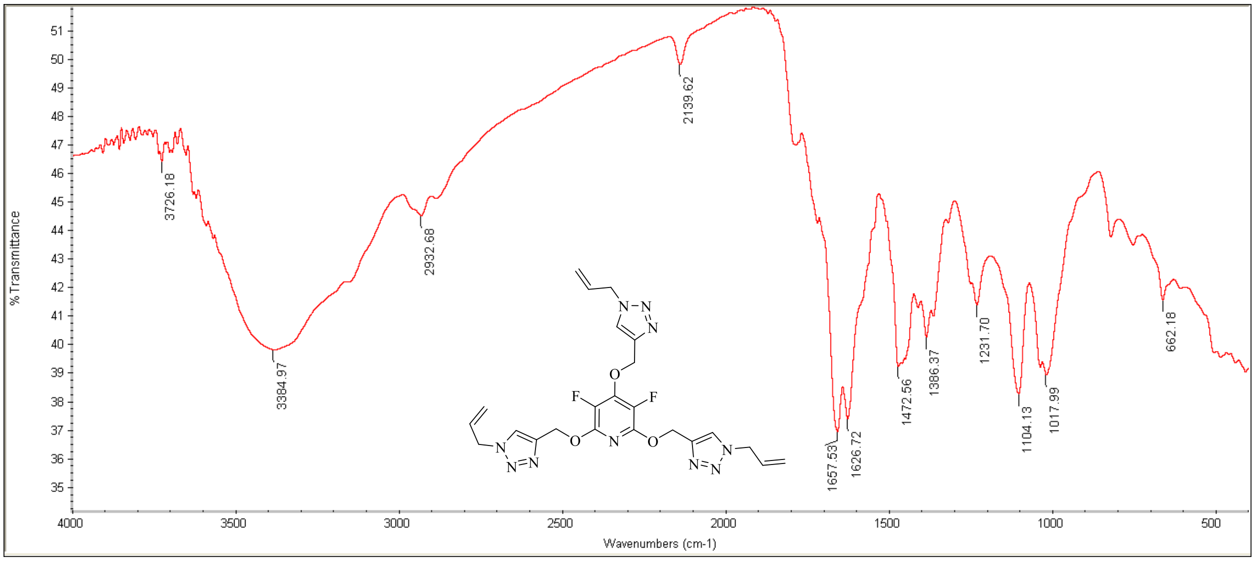

FT-IR spectrum of **7b**

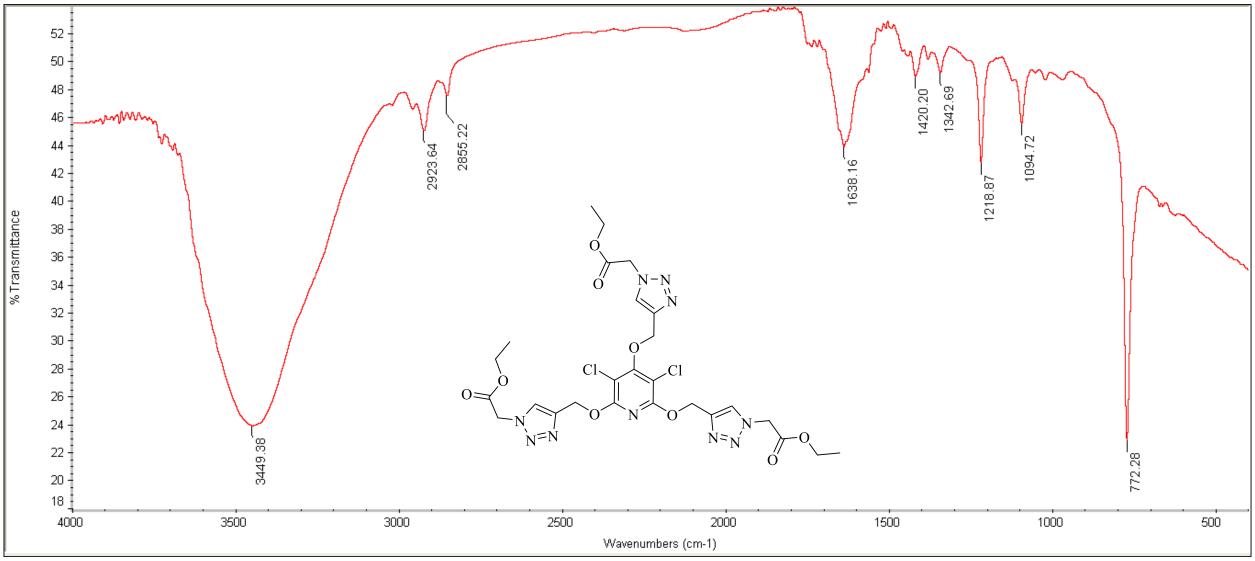

FT-IR spectrum of **7c**

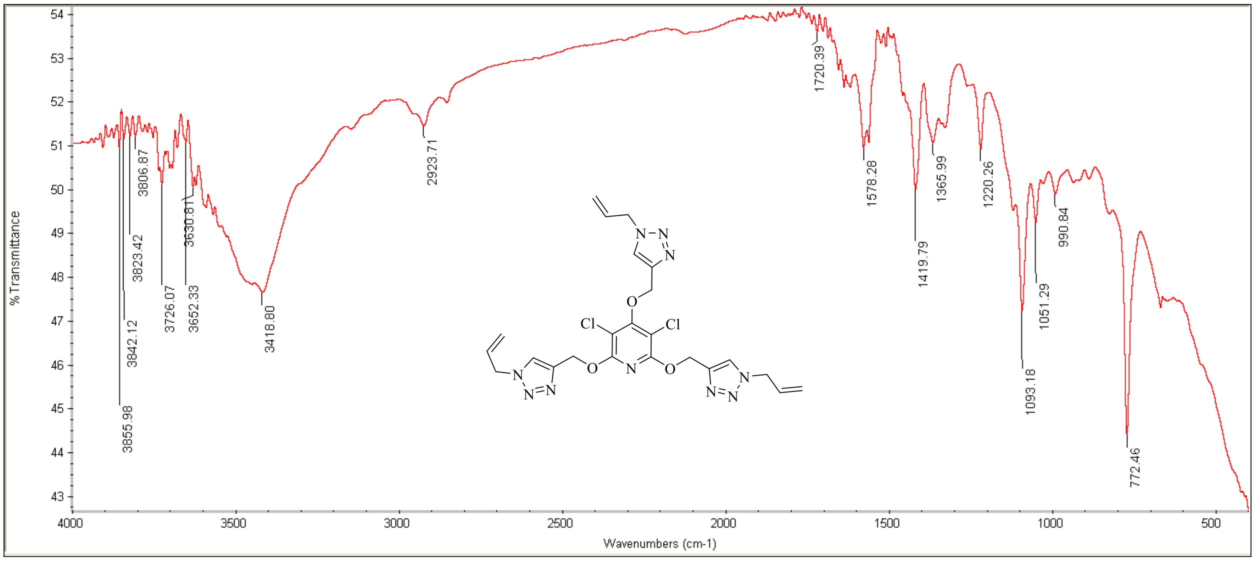

FT-IR spectrum of **7d**

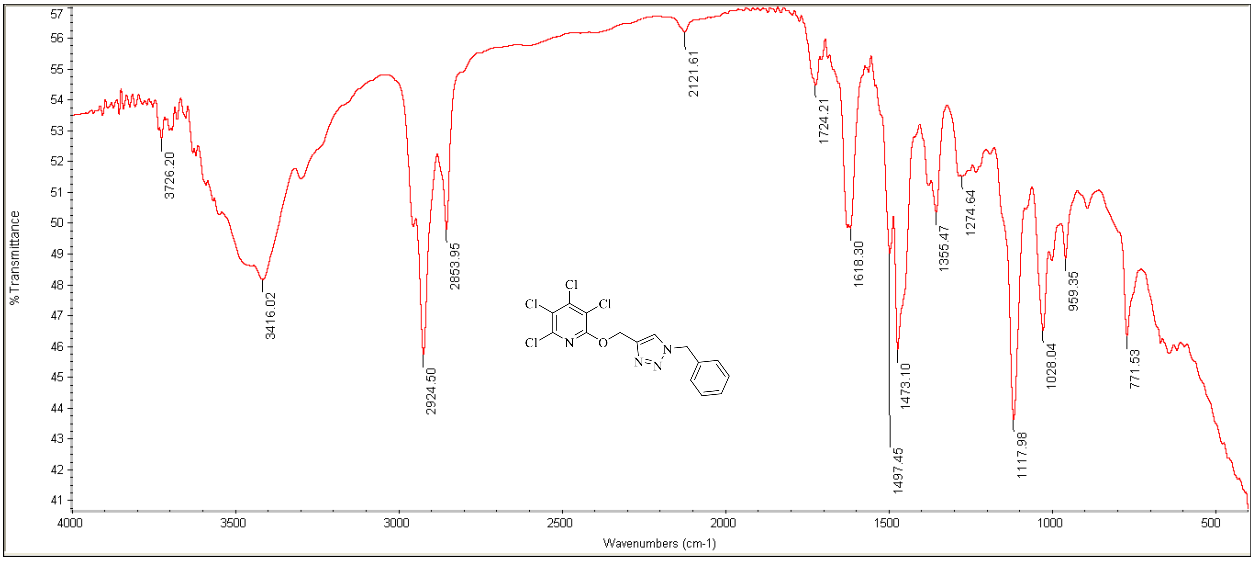

FT-IR spectrum of **8a**

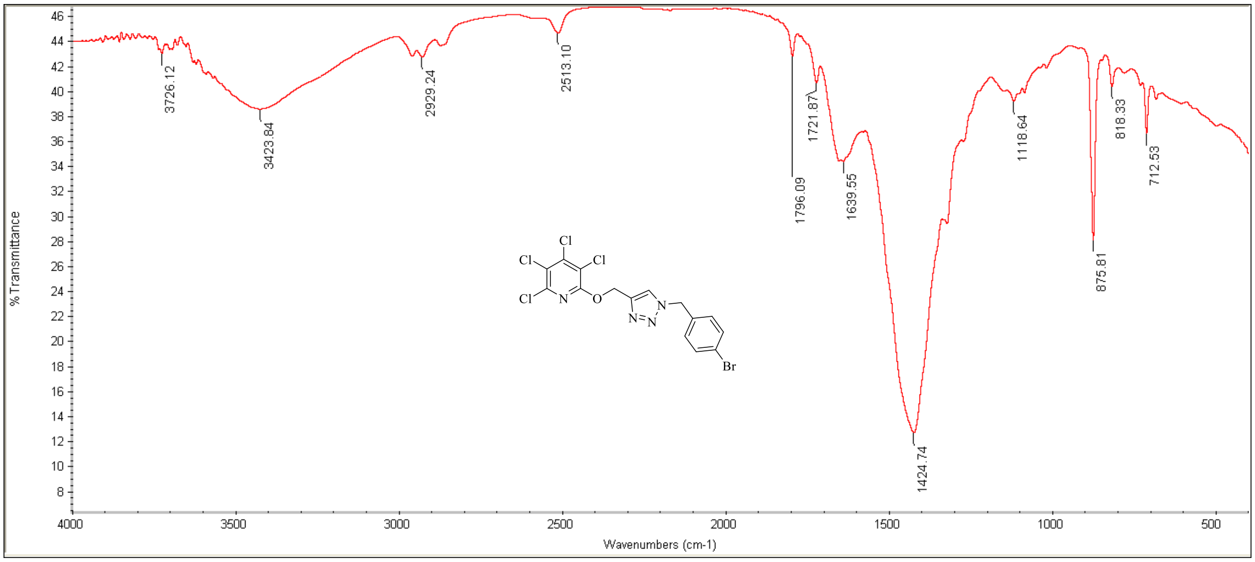

FT-IR spectrum of **8b**

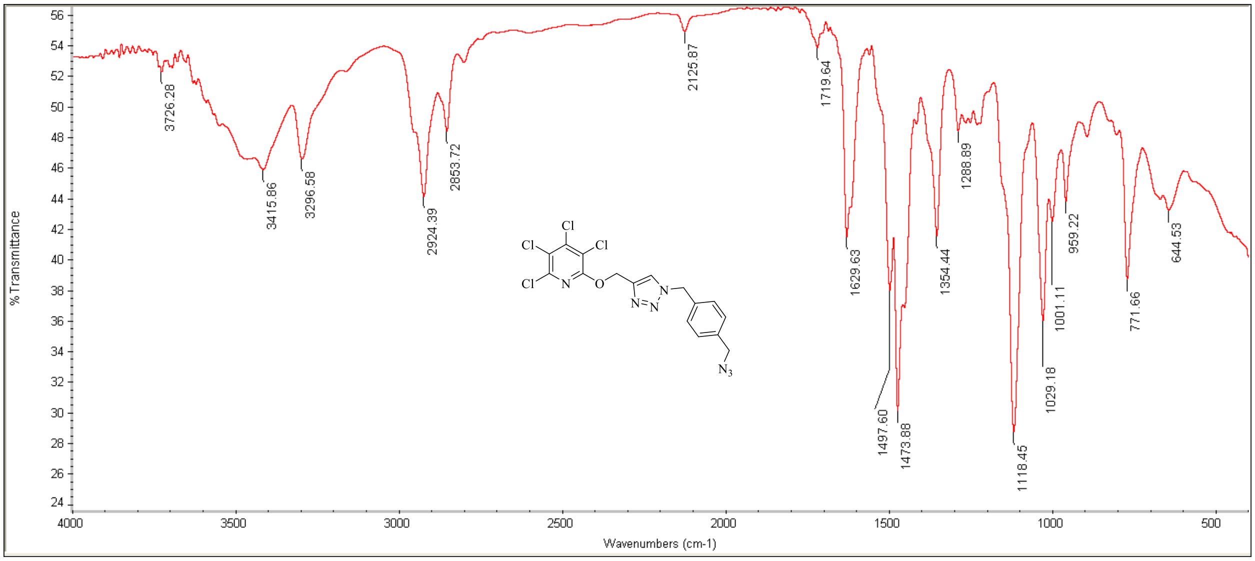

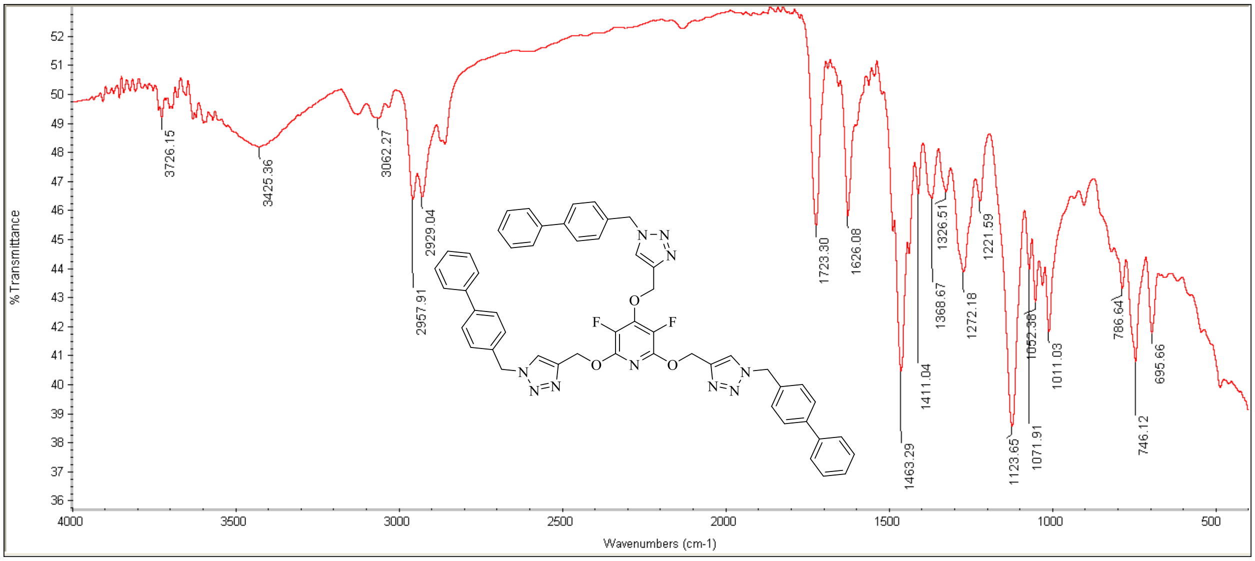

FT-IR spectrum of **10a**

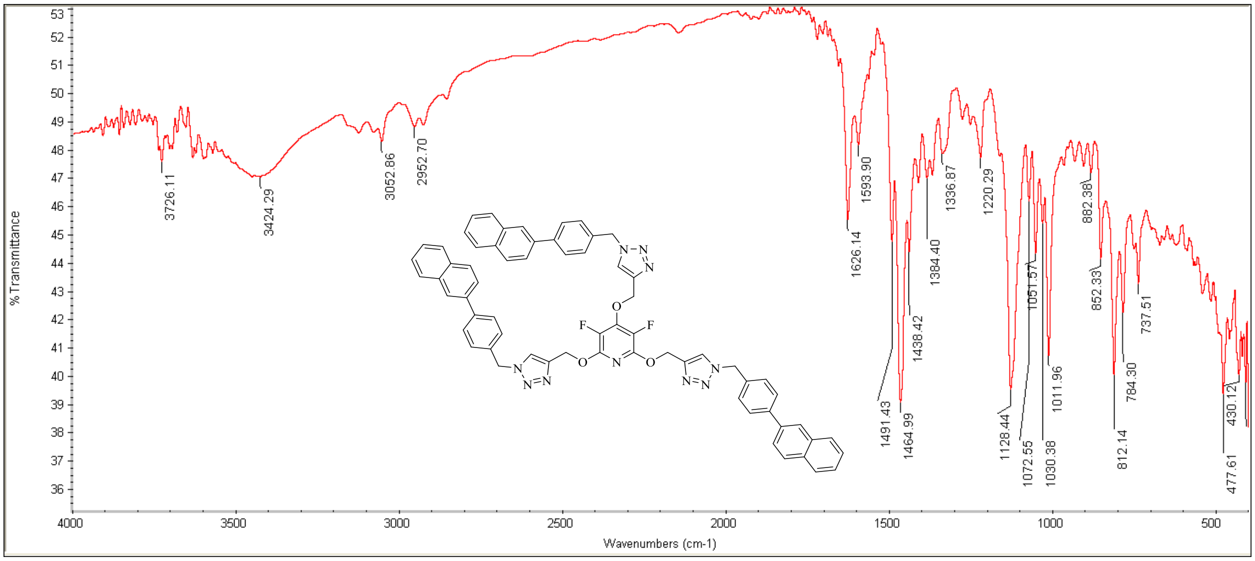

FT-IR spectrum of **10b**

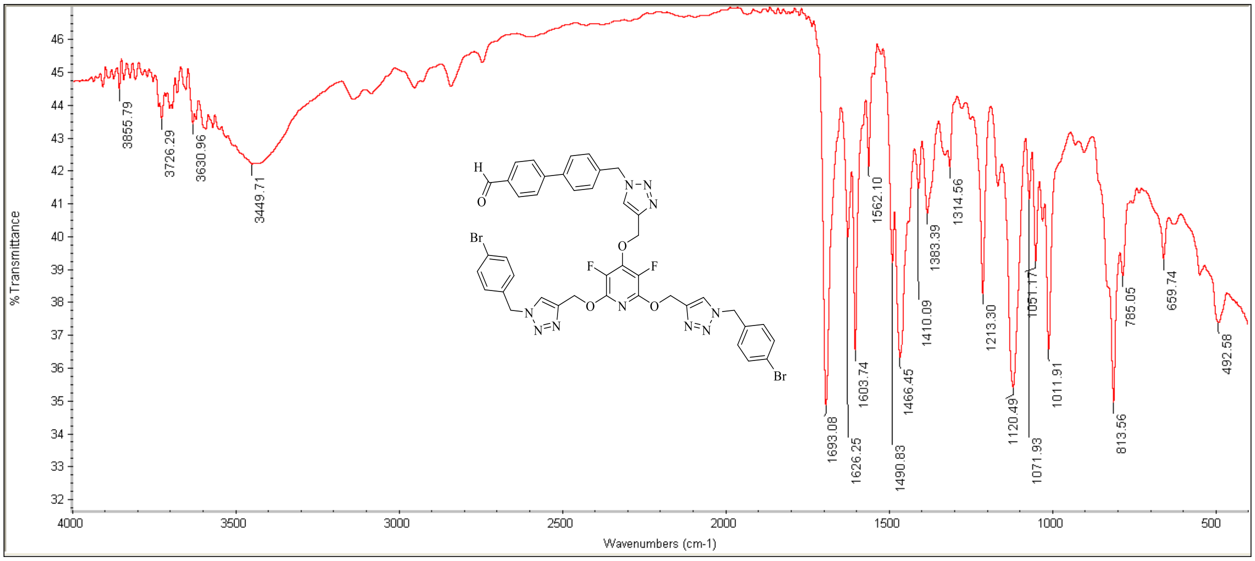

FT-IR spectrum of **10c**

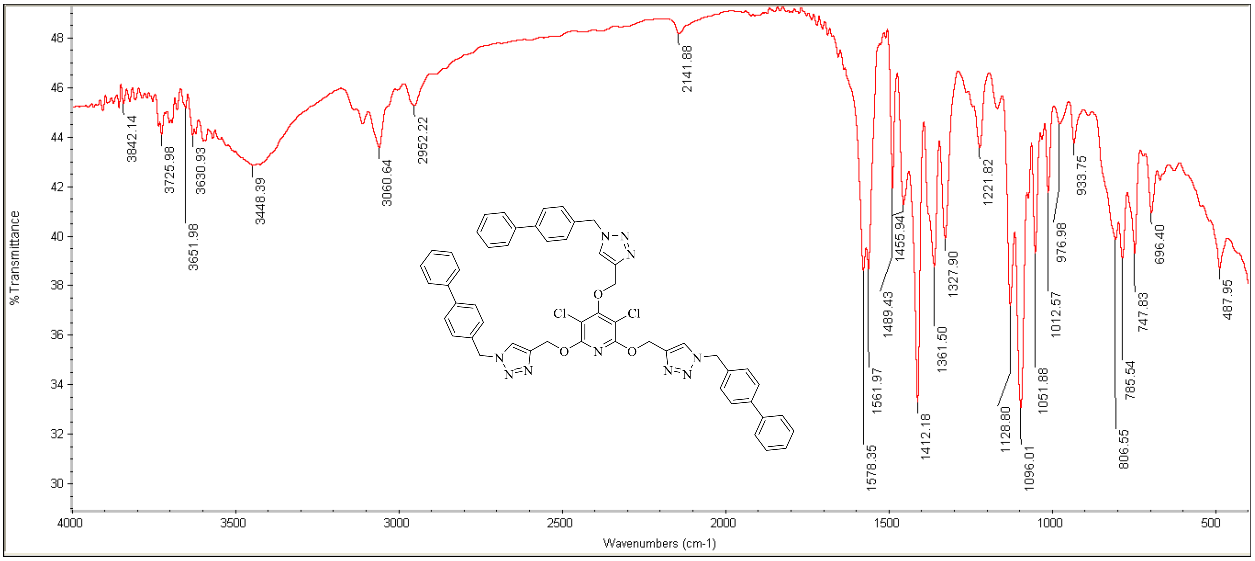

FT-IR spectrum of **10d**

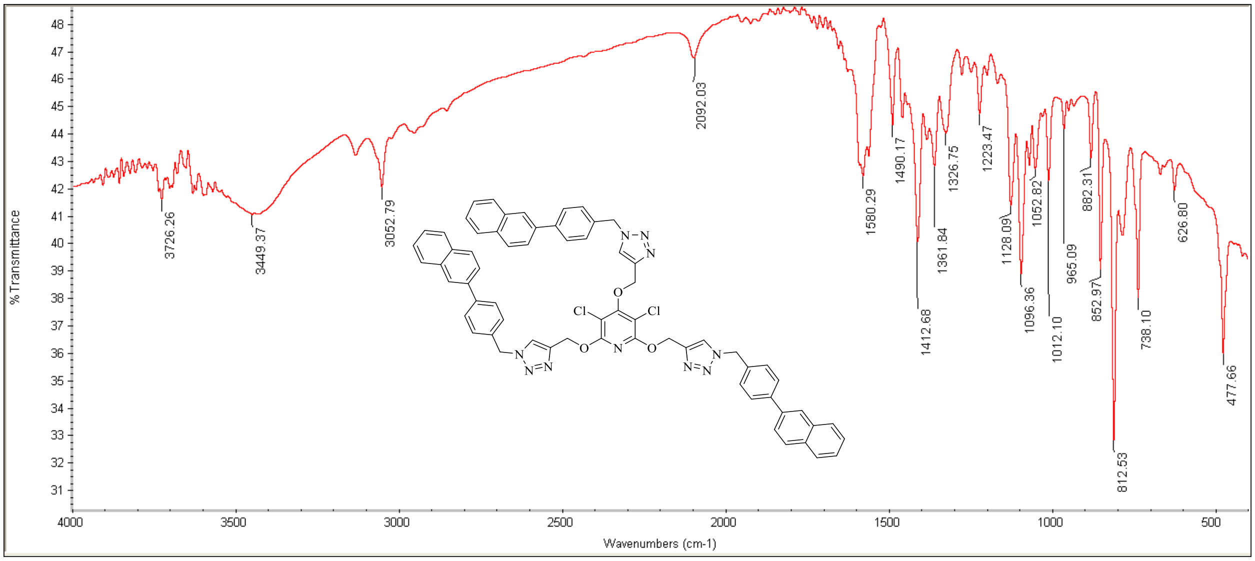

FT-IR spectrum of **10e**

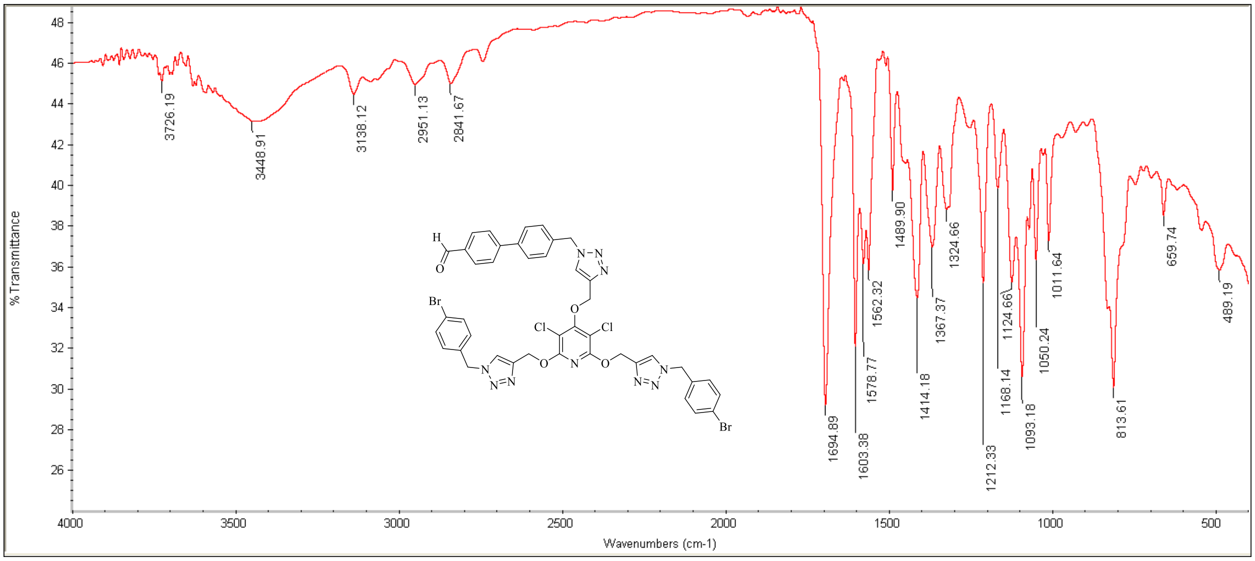

FT-IR spectrum of **10f**
